# A Mild and Highly Diastereoselective Preparation of *N*-Alkenyl-2-Pyridones via 2-Halopyridinium Salts and Aldehydes

**DOI:** 10.1021/acs.joc.1c01566

**Published:** 2021-08-31

**Authors:** Grant N. Shivers, F. Christopher Pigge

**Affiliations:** Department of Chemistry, University of Iowa, Iowa City, Iowa 52242, United States

## Abstract

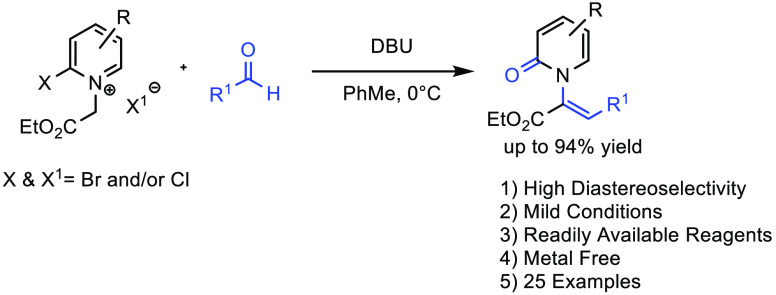

An experimentally simple one-pot preparation of *N*-alkenyl-2-pyridones is reported. The reaction features mild conditions using readily available 2-halopyridinium salts and aldehydes. *N*-Alkenyl-2-pyridone formation proceeds with high diastereoselectivity, and a wide range of aldehyde reaction partners is tolerated. Pyridone products are also amenable to further manipulation, including conversion to *N*-alkyl pyridones and polycyclic ring systems.

*N*-Alkyl-2-pyridones are important heterocycles encountered in various active pharmaceutical ingredients, bioactive compounds, and natural products (Figure 1).^1−7^ Accordingly, a diverse set of methods to prepare these valuable compounds is highly desired.^4,5,8−27^ While a straightforward means of obtaining *N*-alkyl-2-pyridones is through direct *N*-alkylation of 2-hydroxypyridine (2-pyridone), this method often suffers from competitive *O*-alkylation.^9,28−32^ Methods to isomerize 2-alkoxypyridines to *N*-alkyl-2-pyridones (*O*- to *N*- alkyl migration) using transition metal catalysts or Brønsted/Lewis acid promoters have also been described.^14,17,19−23,25−27^

**Figure 1:**
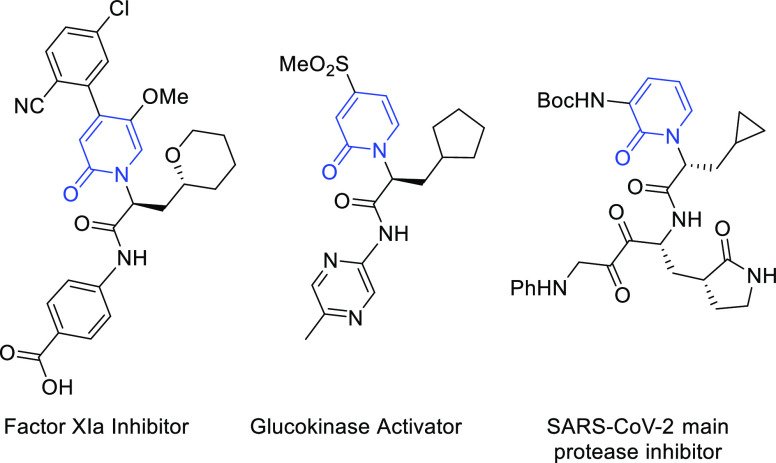
Examples of biologically active 2-pyridones.

Related *N*-alkenyl-2-pyridones have been reported as key prochiral intermediates in the preparation of biologically relevant compounds.^5^ The synthesis of *N*-alkenyl-2-pyridones, however, presents several unique challenges, which are summarized in Scheme 1. Direct coupling of 2-pyridone with alkenyl derivatives has proven to be of limited scope (Scheme 1a). While Chan-Lam couplings are typically robust, the required vinyl boronic acids have limited commercial availability, and the reactions require stoichiometric copper. Ullman-Buchwald and addition–elimination type reactions between 2-pyridones and alkenyl halides are effective, but the required vinyl halides also suffer from lack of widespread commercial availability and thus require additional synthetic operations for their preparation.^33,34^ Silica/LiI promoted rearrangement of *O*-propargylated 2-pyridines affords *N*-alkenyl pyridone products, but reaction scope is limited and yields are modest (Scheme 1b).^14^ Recently an Ir-catalyzed allylic substitution-isomerization reaction was disclosed in which the authors were able to prepare a variety of axially chiral *N*-alkenyl pyridones (Scheme 1c).^35^ Aldol-type condensations between preformed *N*-alkylpyridones and aldehydes is of potentially wide scope and delivers more complex olefin substitution patterns (Scheme 1d).^4,5,9^ However, this tactic requires the use of strong base and preparation of the *N*-alkylpyridone reactants often suffer from competitive *O*-alkylations to provide product mixtures that require careful purification.^9,28,29^ We envisioned a new strategy to access *N*-alkenyl-2-pyridones that relies on aldol like condensations between *N*-alkyl-2-halopyridinium salts and aldehydes in order to circumvent shortcomings of the aforementioned methods (Scheme 1e). Specifically, using 2-halopyridinium salts as precursors to 2-pyridones obviates issues related to *N*- versus *O*-pyridone alkylation while simultaneously activating the 2-halo substituent toward substitution with an O nucleophile (*vide infra*). We report the successful implementation of this approach to construct functionalized *N*-alkenyl-2-pyridones in high yield and high diastereoselectivity.

**Scheme 1:**
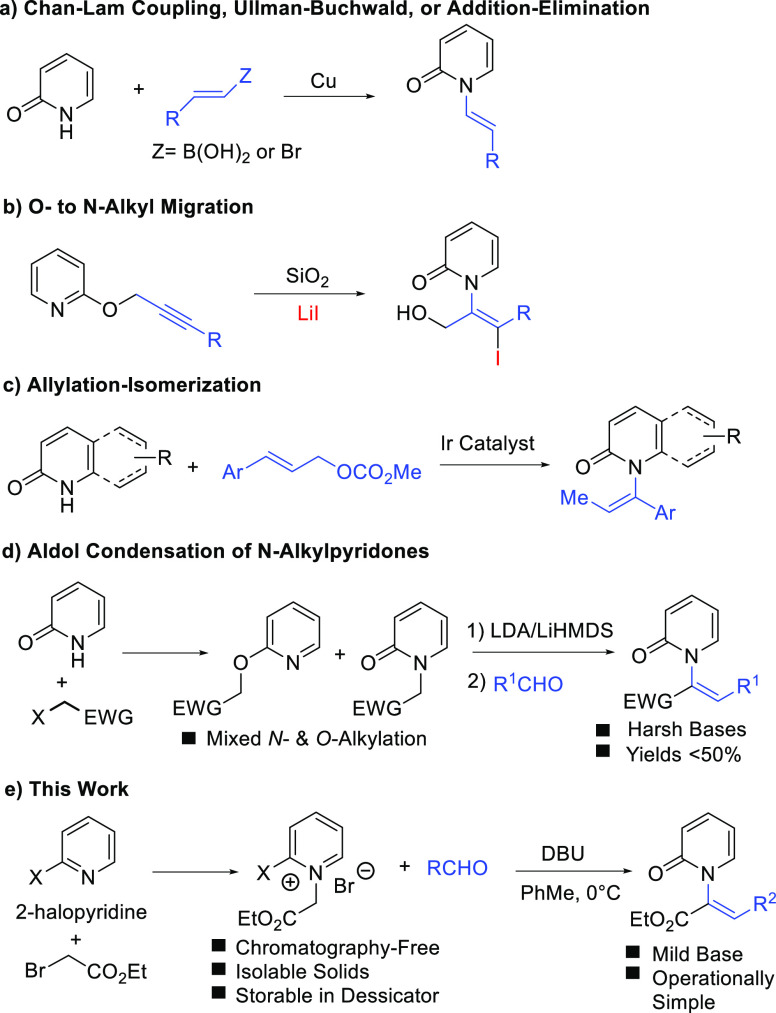
Synthetic Strategies toward *N*-Alkenyl-2-Pyridones

Selected results of initial experiments involving the reaction between 2-bromopyridinium salt **1** (prepared in 81% yield by reaction of 2-bromopyridine and ethyl bromoacetate) and 4-nitrobenzaldehyde (**2a**) leading to *N*-alkenyl-2-pyridone **3a** are outlined in Table 1. Solvents in which **1** was completely soluble (DMF, DMSO) were initially examined along with excess DBU as base owing to the reported p*K*_a_ values of pyridinium salts.^36^ Gratifyingly, the first set of conditions tested gave the desired product in moderate yield (Table 1, entry 1). Performing the reaction in DMSO, however, gave **3a** in a significantly decreased yield (entry 2). Lowering the reaction temperature resulted in increased product yield (entry 3), as did switching to more nonpolar solvents (particularly toluene) despite the heterogeneity of the reaction mixtures. The amount of DBU and aldehyde reactant also could be reduced to 2.1 and 1.0 equiv, respectively, without adversely affecting the isolated yield of **3a**. Inorganic bases yielded no product under the reported conditions (entries 7 and 8), and Et_3_N gave the desired product in a slightly lower yield than DBU (entry 9). The reaction conditions shown in entry 6 were selected for subsequent experiments.

**Table 1:**
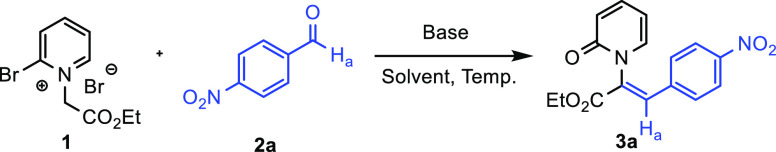
Optimization of Conditions

entry	solvent	base (equiv)	**2a** (equiv)	temp (°C)	isolated yield (%)	*Z*/*E*
1	DMF	DBU (4)	2	RT	59	>20:1
2	DMSO	DBU (4)	2	RT	10	>20:1
3	DMF	DBU (4)	2	0	79	>20:1
4	DCM	DBU (4)	2	0	79	>20:1
5	PhMe	DBU (4)	2	0	92	>20:1
6	PhMe	DBU (2.1)	1	0	90	**>**20:1
7	PhMe	KO^t^Bu (2.1)	1	0	NR	NA
8	PhMe	K_2_CO_3_ (2.1)	1	0	NR	NA
9	PhMe	Et_3_N (2.1)	1	0	85	>20/1

High *Z*/*E* diastereomeric ratios were observed for **3a** under all reaction conditions. The major Z stereoisomer was assigned on the basis of ^1^H NMR spectroscopy and by analogy to previously reported compounds.^9^ Specifically, the vinylic hydrogen H_a_ appears significantly downfield (∼7.9 ppm) in the Z isomer (shown) compared to the E diastereomer (H_a_ ∼ 7.0 ppm). This structural assignment was later confirmed through X-ray crystallography (*vide infra*).

The compatibility of this transformation with different aldehyde reactants was next examined. Benzaldehydes substituted with various electron-withdrawing functional groups (EWG’s) as well as weak electron-donating groups (EDG’s) at the para position gave the desired reaction in reasonable isolated yields (Table 2, **3a**–**3e**), including the preparation of **3a** on a 1.5 and 6.15 mmol (2 g) scale). A strong *p*-EDG resulted in significantly reduced yield under standard reaction conditions (Table 2, 3f). Performing the reaction on a 1.5 mmol scale in the presence of excess *p*-anisaldehyde returned **3f** in a much improved 64% isolated yield. The reaction tolerated both EDG’s and EWG’s at the meta position of the aromatic ring (**3g**–**3l**). Heteroaromatic aldehydes performed very well, delivering the corresponding *N*-alkenyl-2-pyridones **3m**–**3r** in good to excellent yields. *Ortho*-substituted benzaldehydes also participated in the reaction to give the expected pyridones in good yield (**3s**–**3t**). In contrast to aromatic aldehydes, aliphatic aldehydes were found to react sluggishly to afford pyridone products in moderate isolated yields (**3u**–**3v**). Notably, the reaction is amenable to a larger scale (1.5 mmol) as indicated for **3f**, **3l**, **3p**, and **3u**. Finally, attempts to prepare *N*-alkenyl pyridones using cinnamaldehyde, piperonal, and pivaldehyde were unsuccessful.

**Table 2:**
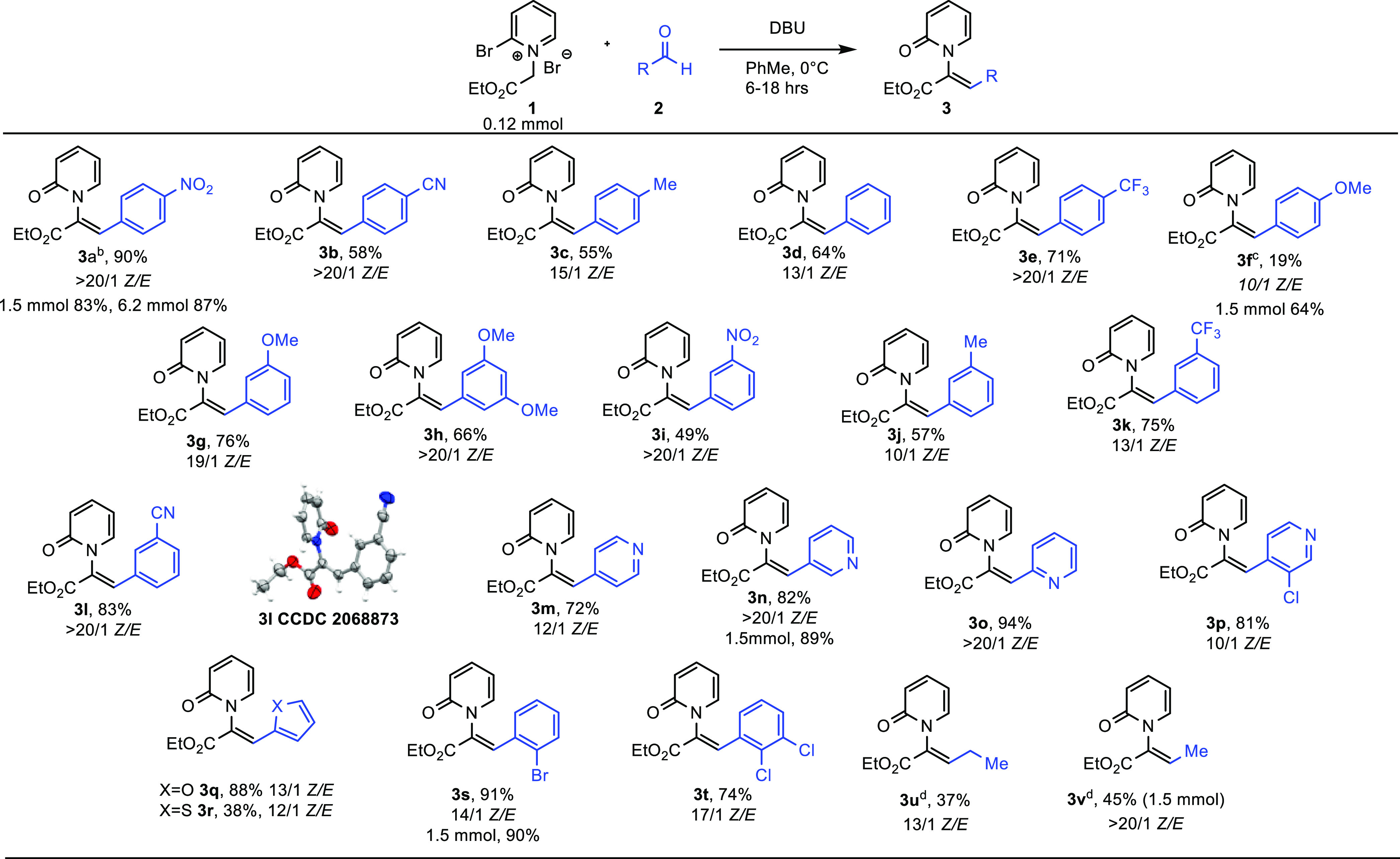
Aldehyde Scope^a^

a Reaction conditions: reactions were run on a 0.12 mmol scale with respect to **1** unless otherwise noted. Isolated yields.

b 30 mg scale.

c 1.5 mmol reaction run with 3 equiv of aldehyde.

d 2 equiv of aldehyde used.

A crystal structure of **3l** was obtained, which confirmed the *Z* configuration of the *N*-alkenyl group. Crystallographic data also revealed that the pyridone and olefin π-system lie nearly orthogonal to each other (Θ∼ 78°), likely to alleviate allylic strain between the pyridone and *N*-alkene substituents. This geometric constraint also impedes delocalization of the nitrogen lone pair through the *N*-olefin π-system, providing a rationale as to why the vinylic hydrogen is significantly downfield in all isolated products. The pyridone nitrogen can only withdraw electron density from the olefin via inductive effects, contributing to the additive deshielding effects of the ester and phenyl substituents. Vinylic hydrogens of the *Z*-isomers appear further downfield than the respective *E*-isomers due to closer proximity to the deshielding region of the ester group.

Several 2-halopyridines with additional pyridine substituents were examined for reactivity as well (Scheme 2). Both 2-chloro-5-methylpyridine and 2-chloro-3-methylpyridine reacted smoothly with ethyl bromoacetate to afford the corresponding pyridinium salts **4a**,**b**.^37−40^ Subsequent condensation with **2a** under our standard reaction conditions gave the expected *N*-alkenyl-2-pyridones **5a** and **5b** in a serviceable yield. Likewise, 2-bromo-5-methoxypyridine was converted to pyridone **5c** in a good overall yield, including on a 1.5 mmol scale. Other 2-halopyridine derivatives examined gave either complex reaction mixtures upon attempted *N*-alkenyl pyridone formation (2-chloro-4-methylpyridine, 2-chloro-3-methoxypyridine) or, in the case of 2-halopyridines with additional electron-withdrawing substituents (halogen, CN, CF_3_), failed to react with ethyl bromoacetate.

**Scheme 2:**
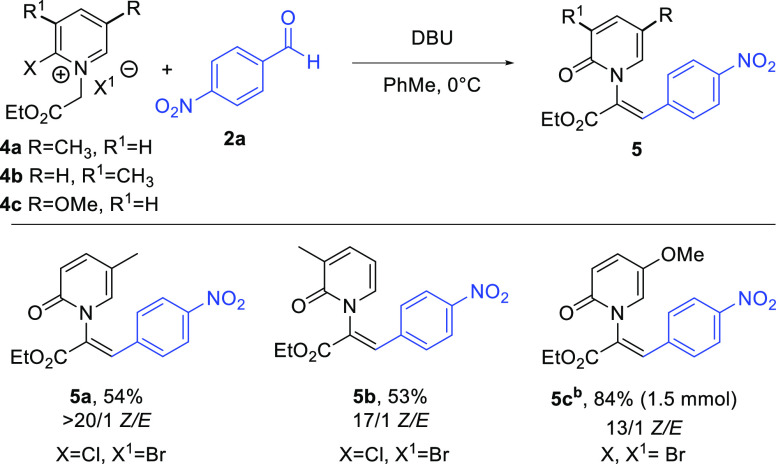
Pyridine Scope Reaction conditions: reactions were run on a 40 mg scale with respect to pyridinium unless otherwise noted. Isolated yields. 1.5 mmol reaction run with a 3 equiv of aldehyde.

Two potential reaction mechanisms are proposed to account for these transformations (Scheme 3). Both entail the initial conversion of the pyridinium salt **1** to the ylide via deprotonation by DBU. The reaction of the ylide and aldehyde partner then gives intermediate **M1**. In pathway 1, an intramolecular S_N_Ar of the alcohol to the 2-position of the activated pyridinium ring generates bicyclic pyridinium **M2**. Ring-opening elimination in the presence of DBU then affords product **3** with the observed Z alkene stereochemistry arising from the preferential formation of trans-substituted intermediate **M2**. Alternatively, pathway 2 features the conversion of **M1** to dehydrated aldol product **M3**.^5,9^ Water generated in this step then participates in an intermolecular S_N_Ar to give **3**. To test the role H_2_O may play in the reaction, **1** and **2a** were combined under the optimized conditions from Table 1 in the presence of 4 Å molecular sieves (Scheme 4a) The expected product **3a**, however, was isolated in <5% yield, implicating an important role for H_2_O consistent with pathway 2. Additionally, exposure of **1** to DBU and excess water in the absence of aldehyde gave only trace amounts of pyridone **6** (Scheme 4b), indicating that the pyridinium ylide intermediate is slow to react with water and pyridone formation is suppressed until intermediate **M3** is formed. The addition of excess water from the onset of the reaction was detrimental to product formation, indicating that control of water concentration is necessary (Scheme 4c).

**Scheme 3:**
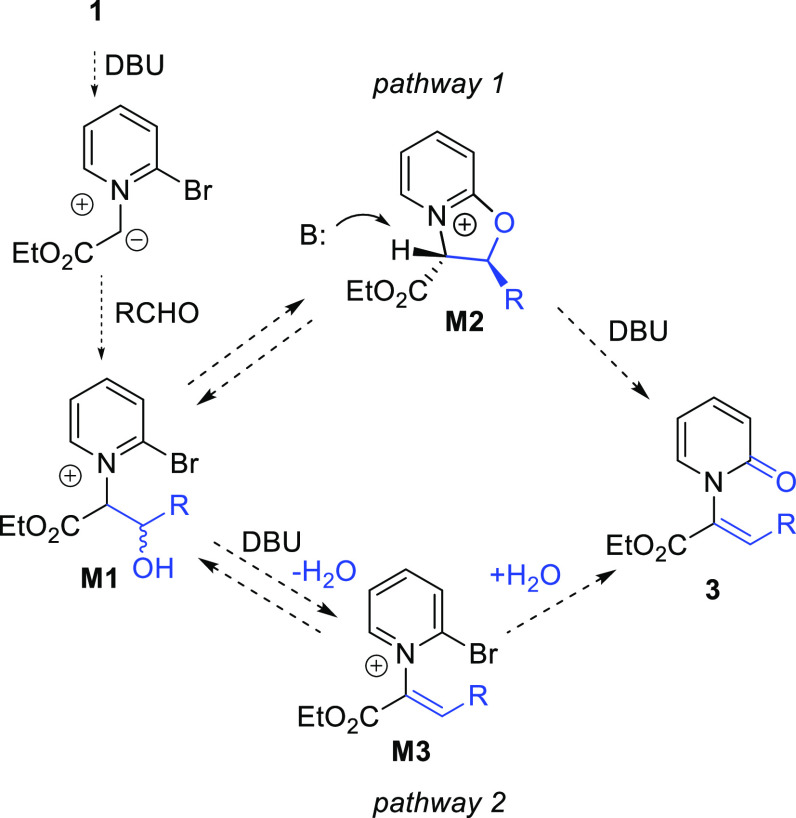
Proposed Mechanisms

**Scheme 4:**
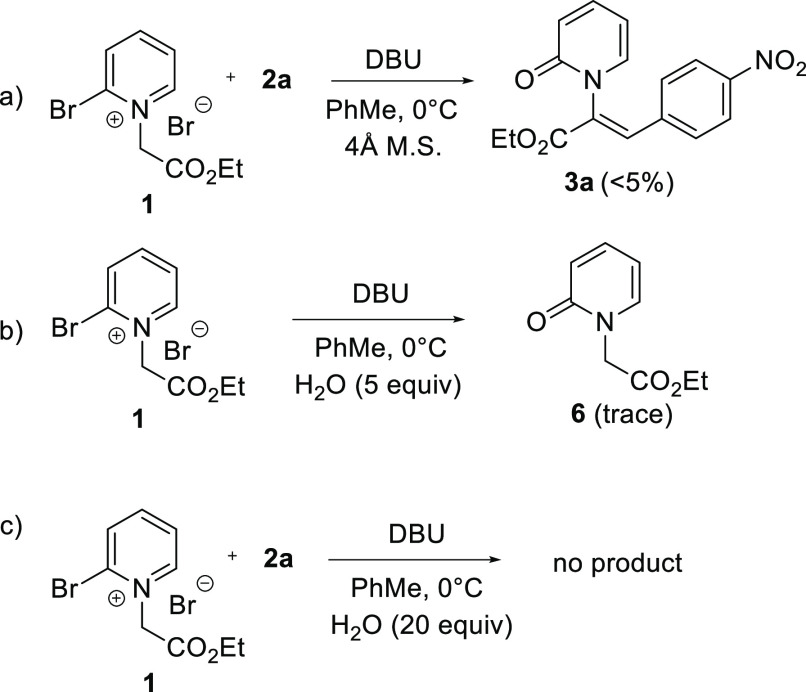
Mechanistic Probes

Pyridone products are amenable to further synthetic manipulations as shown in Scheme 5. The nitro group of **3a** was successfully reduced to aniline **7**, installing an EDG that would be challenging to incorporate directly via this methodology and providing a handle for further elaboration. Selective reduction of the *N*-vinyl alkene in **3f** was achieved using (BDP)CuH, a catalytic source of Stryker’s reagent, affording **8** in a high isolated yield.^41^ Finally, **3s** was subjected to an intramolecular Heck reaction, affording tricyclic product **9**. The ^1^H NMR spectrum of **9** showed that the vinylic hydrogen H_a_ is shifted upfield relative to **3s**, as the pyridone and olefin π-systems are now fully conjugated, allowing nitrogen lone pair donation into the exocyclic olefin. This further supports the claim that the orthogonal relationship between the pyridone and olefin π-systems causes the observed downfield shift of the vinylic hydrogen in *Z*-*N*-alkenyl-2-pyridones **3**.

**Scheme 5:**
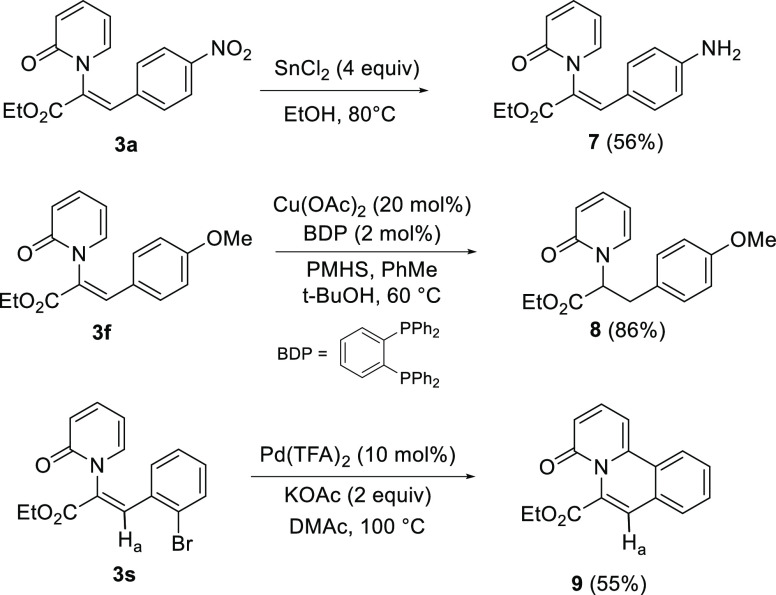
Synthetic Manipulation of *N*-Alkenyl Pyridones

In conclusion, a mild and operationally simple route to *N*-vinyl-2-pyridones has been developed that proceeds in high yield and high diastereoselectivity. The reaction tolerates a wide range of aldehydes, with electron-deficient aldehydes performing most effectively, and delivers functionalized *N*-alkenyl-2-pyridones capable of undergoing additional synthetic elaboration. The convenience and simplicity of this method nicely complement established routes to valuable pyridone derivatives.

## Experimental Section

### General Considerations

Unless otherwise noted, reactions were run under an argon atmosphere in oven-dried glassware. Reactions requiring heat were performed in two-neck flasks equipped with a reflux condenser in a mineral oil bath heated with a Staco-Energy Variac Model 3PN1010B. When necessary, solvents were dried and purified prior to experiments using a PureSolv MD5 solvent purification system. Reactions run at 0 °C were chilled in a water bath using a Thermo Scientific immersion cooler (HAAKE Pheonix II). Rotary evaporation was performed using a Model 2027 Welch Pump with a water bath preheated to 40 °C. Commercial reagents were purchased from Acros Organics, Alfa Aesar, Sigma-Aldrich Chemical Co., TCI Chemical, or Oakwood Chemical and used without further purification unless otherwise noted.

Analytical thin-layer chromatography (TLC) was performed on Sorbtech 200 μm silica gel UV254 plates and visualized with UV light and/or KMnO_4_ staining. Chromatographic purification was performed using the indicated solvent system on Silicycle SilicaFlash F60 silica gel (230–400 mesh). ^1^H NMR spectra were recorded on a Bruker Avance, Bruker Ascend, or Bruker DRX-400 MHz spectrometer (CDCl_3_ = 7.26 ppm or TMS= 0.00 ppm). Data are reported as follows: chemical shift in delta units (δ), multiplicity (s = singlet, d = doublet, t = triplet, q = quartet, p = pentet, dt = doublet of triplets, td = triplet of doublets, m = multiplet), coupling constants (reported in Hz), and integration value. Decoupled ^13^C NMR spectra were recorded at 100 MHz with deuterated chloroform as a standard (CDCl_3_ = 77.16 ppm). ^19^F NMR spectra were recorded at 376 MHz, using monofluorobenzene (δ_F_ = −113.5 ppm) as an internal standard. High-resolution mass spectra (HRMS) were obtained using a Waters Q-ToF Premier mass spectrometer using positive ion electrospray ionization (ESI). Melting points were recorded using a capillary melting point apparatus and are uncorrected.

#### Procedure for the Synthesis of 2-Halopyridinium Salts

2-Halopyridine (1 equiv) and ethyl bromoacetate (5 equiv) were added to a two-neck flask charged with a stir bar equipped with a reflux condenser under argon. Reactions were then heated in an 85 °C oil bath for 24 h while stirring. Reactions were allowed to cool to room temperature (rt), diluted with ether (10 mL), and stirred for an additional 10 min. The desired pyridinium salt was collected by vacuum filtration, rinsed with ether (2 × 5 mL), and dried first in air and then under a vacuum. Materials were used in subsequent reactions without further purification. Note that the yields indicated for **4a** and **4b** were calculated assuming no halogen exchange occurs with ethyl bromoacetate. When electron-withdrawing groups were attached to the pyridine ring (CN, CF_3_, Br), no products could be isolated under these conditions.

##### Pyridinium Salt **1**

2-Bromopyridine (0.50 mL, 5.2 mmol) and ethyl bromoacetate (2.9 mL, 26.2 mmol) gave **1** as faint yellow needles (1.39 g, 81%). Mp: 173–178 °C (dec).^42^

##### Pyridinium Salt **4a**

2-Chloro-5-methylpyridne (0.50 g, 2.91 mmol) and ethyl bromoacetate (1.6 mL, 14.5 mmol) gave **4a** (0.75 g, 76%) as a tan powder. Mp: 144–145 °C (dec).

##### Pyridinium Salt **4b**

2-Chloro-3-methylpyridine (0.50 mL, 4.6 mmol) and ethyl bromoacetate (2.50 mL, 22.9 mmol) gave **4b** (1.23 g, 91%) as a white powder. Mp: 170–171 °C (dec).

##### Pyridinium Salt **4c**

2-Bromo-5-methoxypyridine (0.50 g, 2.7 mmol) and ethyl bromoacetate (1.5 mL, 13.3 mmol) gave **4c** (1.04 g, 77%) as a yellow powder. Mp: 141–146 °C (dec).

### Condensation-S_N_Ar Optimization Conditions (Table 1)

Compound **1** (30 mg, 0.092 mmol) and the indicated quantity of **2a** were added to a 25 mL two-neck flask charged with a stir bar under argon. The solids were dissolved/suspended in solvent (1 mL), followed in some cases by cooling to ∼0 °C in an ice water bath for 20–30 min. A base was added, and the reaction was stirred and maintained at the indicated temperature. Upon completion of the reaction (determined by the consumption of aldehyde by TLC or overnight if all of the aldehyde is not consumed), the reaction mixture was concentrated via rotary evaporation, and the residual material was purified via flash column chromatography (3:2 EtOAc/Hex to 2:1 EtOAc/Hex). *Z*/*E* ratios of isolated products were determined by ^1^H NMR.

#### General Procedure for Preparation of *N*-Alkenyl-2-Pyridones

2-Halopyridinium salt (40 mg unless otherwise noted) and aldehyde (1 equiv unless otherwise noted) were combined in an oven-dried two-neck flask charged with a stir bar under argon. Toluene (1.2 mL) was added, and the heterogeneous mixture was cooled to ∼0 °C in an ice water bath for 20–30 min. DBU was then added via syringe, and the reaction was maintained at 0 °C. Upon completion of the reaction (determined by the consumption of aldehyde (TLC) or run overnight if aldehyde was not completely consumed), the reaction mixture was concentrated via rotary evaporation, and the residual material was purified via flash column chromatography. *Z*/*E* ratios were determined by ^1^H NMR.

##### *N*-Alkenyl-2-pyridone **3a**

The compound was prepared using the general procedure starting with 30 mg of **1** and obtained as a yellow solid (26 mg, 90% yield): mp = 132–134 °C. The reaction performed using 1.5 mmol **1** in 15 mL of toluene afforded 0.393 g (83%) of **3a**: chromatography conditions 3:2 EtOAc/Hex to 2:1 EtOAc/Hex; ^1^H NMR (400 MHz, CDCl_3_) δ 8.18 (m, 2H), 7.87 (s, 1H), 7.47 (ddd, *J* = 9.4, 6.6, 2.1, 1H), 7.39 (m, 2H), 6.92 (ddd, *J* = 6.9, 2.1, 1.0 Hz, 1H), 6.69 (dt, *J* = 9.4, 1.0 Hz, 1H), 6.22 (td, *J* = 6.7, 1.2 Hz, 1H), 4.37 (q, *J* = 7.1 Hz, 2H), 1.35 (t, *J* = 7.1 Hz, 3H); ^13^C{^1^H} NMR (100 MHz CDCl_3_) δ 162.8, 162.4, 148.4, 141.2, 138.0, 137.1, 134.9, 133.5, 130.5, 124.2, 122.1, 107.3, 62.6, 14.2; HRMS (ESI) calcd for C_16_H_15_N_2_O_5_ [M + H]^+^ 315.0975, found 315.0972.

##### Gram Scale Preparation of **3a**

Pyridinium salt **1** (2.00 g, 6.15 mmol) and *p*-nitrobenzaldehyde (0.93 g, 6.15 mmol) were added to an oven-dried 250 mL two-neck flask charged with a stir bar while under argon. The solids were then dissolved/suspended in toluene (60 mL) and cooled to 0 °C in an ice bath for 30 min. DBU (2.0 mL, 13 mmol) was then added via syringe. The reaction was allowed to stir for 16 h at 0 °C. Reaction contents were then transferred to a 250 mL RBF, rinsed with DCM, and concentrated via rotary evaporation. The residue was dissolved in EtOAc (50 mL) and washed with 50 mL of 1 M HCl. The aqueous phase was then extracted with additional EtOAc (2 × 50 mL), and the combined organic layer was dried over anhydrous Na_2_SO_4_. Filtration and removal of the solvent gave an orange solid that was purified by recrystallization from DCM to afford **3a** (1.65 g, 86% yield) as a yellow solid.

##### *N*-Alkenyl-2-pyridone **3b**

The compound was prepared using the general procedure and obtained as a yellow oil (21 mg, 58% yield): chromatography conditions 2:1 EtOAc/Hex; ^1^H NMR (400 MHz, CDCl_3_) δ 7.82 (s, 1H), 7.60 (d, *J* = 8.4 Hz, 2H), 7.47 (ddd, *J* = 9.4, 6.6, 2.0 Hz, 1H), 7.31 (d, *J* = 8.2 Hz, 2H), 6.92 (ddd, *J* = 6.8, 2.0, 0.7 Hz, 1H), 6.69 (9.3, 0.9 Hz, 1H), 6.22 (td, *J* = 6.7, 1.2 Hz, 1H), 4.36 (q, *J* = 7.1 Hz, 2H), 1.35 (t, *J* = 7.1 Hz, 3H); ^13^C{^1^H} NMR (100 MHz CDCl_3_) δ 162.9, 162.4, 141.1, 137.2, 136.1, 135.4, 133.1, 132.7, 130.2, 122.1, 118.1, 113.8, 107.2, 62.6, 14.2; HRMS (ESI) calcd for C_17_H_15_N_2_O_3_ [M + H]^+^ 295.1077, found 295.1078.

##### *N*-Alkenyl-2-pyridone **3c**

The compound was prepared using the general procedure and obtained as an orange oil (19 mg, 55% yield): chromatography conditions 2:1 Hex/EtOAc to 1:1 Hex/EtOAc; ^1^H NMR (400 MHz, CDCl_3_) δ 7.81 (s, 1H), 7.50 (ddd, *J* = 9.3, 6.6, 2.1 Hz, 1H), 7.11 (d, *J* = 8.2 Hz, 2H), 7.07 (d, *J* = 8.4 Hz, 2H), 7.03 (ddd, *J* = 6.8, 2.0, 0.6 Hz), 1H), 6.80 (d, *J* = 9.3 Hz, 1H), 6.27 (td, *J* = 6.7, 1.2 Hz, 1H), 4.33 (q, *J* = 7.1 Hz, 2H), 2.32 (s, 3H), 1.33 (t, *J* = 7.1 Hz, 3H); ^13^C{^1^H} NMR (100 MHz CDCl_3_) δ 163.6, 162.5, 141.4, 141.2, 138.1, 137.8, 130.2, 129.9, 129.1, 128.7, 121.7, 107.5, 62.1, 21.6, 14.2; ^13^C{^1^H} NMR (100 MHz CDCl_3_) δ calcd for C_17_H_18_NO_3_ [M + H]^+^ 284.1281, found 284.1274.

##### *N*-Alkenyl-2-pyridone **3d**(9,43)

The compound was prepared using the general procedure and obtained as a tan solid, (21 mg, 64%): mp 99–102 °C; chromatography conditions 2:1 Hex/EtOAc to 3:2 EtOAc/Hex; ^1^H NMR (400 MHz, CDCl_3_) δ 7.83 (s, 1H), 7.46 (ddd, *J* = 9.3, 6.6, 2.1 Hz, 1H), 7.34 (m, 3H), 7.20 (m, 2H), 6.98 (ddd, *J* = 6.8, 2.0, 0.6 Hz, 1H), 6.70 (d, *J* = 9.3 Hz, 1H), 6.20 (td, *J* = 6.7, 1.2 Hz, 1H), 4.34 (q, *J* = 7.1 Hz, 2H), 1.34 (t, *J* = 7.1 Hz, 3H); ^13^C{^1^H} NMR (100 MHz CDCl_3_) δ 163.5, 162.5, 140.9, 137.9, 137.6, 131.6, 130.6, 130.3, 130.1, 129.1, 121.9 107.0, 62.1, 14.2. Matches previous characterization data.

##### *N*-Alkenyl-2-pyridone **3e**

The compound was prepared using the general procedure and obtained as a clear oil (30 mg, 71% yield); chromatography conditions 1:1 EtOAc/Hex; ^1^H NMR (400 MHz, CDCl_3_) δ 7.85 (s, 1H), 7.57 (d, *J* = 8.3 Hz, 2H), 7.47 (ddd, *J* = 9.4, 6.6, 2.1 Hz, 1H), 7.33 (d, *J* = 8.3 Hz, 2H), 6.94 (ddd, *J* = 6.9, 2, 0.7 Hz, 1H), 6.69 (dt, *J* = 9.3, 1 Hz, 1H), 6.21 (td, *J* = 6.7, 1.2 Hz, 1H), 4.36 (q, *J* = 7.1 Hz, 2H), 1.35 (t, *J* = 7.1 Hz, 3H); ^13^C{^1^H} NMR (100 MHz CDCl_3_) δ 163.1, 162.5, 141.0, 137.4, 135.9, 135.2, 132.4, 132.1 (q, *J* = 33 Hz), 130.1, 127.8 (q, *J* = 273 Hz), 126.0 (q, *J* = 4 Hz), 122.1, 107.1, 62.5, 14.2; ^19^F NMR (376 MHz, CDCl_3_) δ −63.1; HRMS (ESI) calcd for C_17_H_15_O_3_F_3_N [M + H]^+^ 338.0999, found 338.0995.

##### *N*-Alkenyl-2-pyridone **3f**

The compound was prepared using the general procedure and obtained as a tan oil (7 mg, 19% yield). The reaction performed using 1.5 mmol **1** and 3 equiv of **2f** in 15 mL of toluene afforded 0.287 g of **3f** (64% yield): chromatography conditions 2:1 EtOAc/Hex to 100% EtOAc; ^1^H NMR (400 MHz, CDCl_3_) δ); 7.78 (s, 1H), 7.47 (m, 1H), 7.14 (d, *J* = 8.8 Hz, 2H), 7.03 (dd, *J* = 6.8, 2.0 Hz, 1H), 6.82 (d, *J* = 8.7 Hz, 1H), 6.70 (dd, *J* = 9.3. 0.6 Hz, 1H), 6.24 (t, *J* = 6.7 Hz, 1H), 4.32 (q, *J* = 7.1 Hz, 2H), 3.78 (s, 3H), 1.32 (t, *J* = 7.1 Hz, 3H); ^13^C{^1^H} NMR (100 MHz CDCl_3_) δ 163.8, 162.4, 161.5, 140.7, 138.1, 137.3, 132.1, 127.7, 124.1, 122.0, 114.5, 106.9, 61.8, 55.4, 14.2; HRMS (ESI) calcd for C_17_H_18_O_4_N [M + H]^+^ 300.1230, found 300.1225.

##### *N*-Alkenyl-2-pyridone **3g**

The compound was prepared using the general procedure and obtained as an opaque white oil (28 mg, 76% yield): chromatography conditions 2:1 EtOAc/Hex; ^1^H NMR (400 MHz, CDCl_3_) δ 7.80 (s, 1H), 7.45 (ddd, *J* = 9.3, 6.6, 2.1, 1H), 7.23 (t, *J* = 8.0 Hz, 1H), 7.00 (ddd, *J* = 6.8, 2.0, 0.6 Hz, 1H), 6.90 (ddd, *J* = 8.3, 2.6, 0.8 Hz, 1H), 6.83 (d, *J* = 7.7, 1H), 6.69 (m, 2H), 6.21 (td, *J* = 6.7, 1.2 Hz, 1H) 4.34 (q, *J* = 7.1 Hz, 2H), 3.65 (s, 3H), 1.35 (t, *J* = 7.1 Hz, 3H); ^13^C{^1^H} NMR (100 MHz CDCl_3_) δ 163.5, 162.5, 159.8, 140.8, 138.0, 137.5, 132.8 130.4, 130.0, 123.0, 122.0, 117.2, 114.0, 106.8, 62.1, 55.2, 14.3; HRMS (ESI) calcd for C_17_H_15_O_3_F_3_N [M + H]^+^ 300.1230, found 300.1229.

##### *N*-Alkenyl-2-pyridone **3h**

The compound was prepared using the general procedure and obtained as a yellow oil (27 mg, 66% yield): chromatography conditions 1:1 EtOAc/Hex to 2:1 EtOAc/Hex; ^1^H NMR (400 MHz, CDCl_3_) δ 7.75 (s, 1H), 7.45 (m, 1H), 7.01 (ddd, *J* = 6.8, 1.9, 0.5 Hz, 1H), 6.69 (dd, *J* = 9.3, 0.6 Hz, 1H), 6.43 (s, 1H), 6.35 (m, 2H), 6.22 (m, 1H), 4.34 (q, *J* = 7.1 Hz, 2H), 3.65 (s, 6H), 1.34 (t, *J* = 7.1 Hz, 3H); ^13^C{^1^H} NMR (100 MHz CDCl_3_) δ 163.5, 162.4, 160.9, 140.8, 138.2, 137.6, 133.19, 130.6, 121.9, 107.6, 106.8, 103.5, 62.18, 55.4, 14.3; HRMS (ESI) calcd for C_18_H_20_O_5_N [M + H]^+^ 330.1336, found 330.1332.

##### *N*-Alkenyl-2-pyridone **3i**

The compound was prepared using the general procedure and obtained as a white solid (19 mg, 49%): mp = 116–120 °C; chromatography conditions 1:1 EtOAc/Hex; ^1^H NMR (400 MHz, CDCl_3_) δ 8.21 (dt, *J* = 7.2, 2.1 Hz, 1H), 8.04 (d, *J* = Hz, 1H), 7.88 (s, 1H), 7.55 (m, 3H), 6.98 (m, 1H), 6.71 (d, *J* = 9.41 Hz, 1H), 6.26 (td, *J* = 6.7, 1.2 Hz, 1H), 4.37 (q, *J* = 7.1 Hz, 2H), 1.36 (t, *J* = 7.1 Hz, 3H); ^13^C{^1^H} NMR (100 MHz CDCl_3_) δ 162.9, 162.2, 148.6, 141.1, 137.0, 135.4, 135.0, 133.3, 132.8, 130.2, 124.9, 124.5, 122.3, 107.4, 62.6, 14.2; HRMS (ESI) [M + H] calcd for C_16_H_15_N_2_O_5_ [M + H]^+^ 315.0975, found 315.0967.

##### *N*-Alkenyl-2-pyridone **3j**

The compound was prepared using the general procedure and obtained as an orange oil (20 mg, 57%); chromatography conditions 2:1 Hex/EtOAc to 1:1 EtOAc/Hex; ^1^H NMR (400 MHz, CDCl_3_) δ 7.80 (s, 1H), 7.48 (ddd, *J* = 9.3, 6.6, 2.1 Hz, 1H), 7.20 (m, 2H), 6.99 (m, 2H), 6.73 (d, *J* = 9.2 Hz, 1H), 6.22 (td, *J* = 6.7, 1.2 Hz, 1H), 4.34 (q, *J* = 7.1 Hz, 2H), 2.25 (s, 3H), 1.34 (t, *J* = 7.1 Hz, 3H); ^13^C{^1^H} NMR (100 MHz CDCl_3_) δ 163.6, 162.6, 140.9, 138.7, 138.0, 137.8, 131.5, 131.5, 130.9, 130.0, 128.9 127.1, 121.9, 107.1, 62.1, 21.4, 14.3; HRMS (ESI) calcd for C_17_H_18_O_3_N [M + H]^+^ 284.1281, found 284.1274.

##### *N*-Alkenyl-2-pyridone **3k**

The compound was prepared using the general procedure and obtained as a clear oil (31 mg, 75% yield): chromatography conditions 1:1 EtOAc/Hex; ^1^H NMR (400 MHz, CDCl_3_) δ 7.84 (s, 1H), 7.60 (d, *J* = 7.8 Hz, 1H), 7.47 (m, 3H), 7.38 (d, *J* = 7.8 Hz, 1H), 6.95 (ddd, *J* = 6.9, 2, 0.7 Hz, 1H), 6.70 (dt, *J* = 9.4, 0.7 Hz, 1H), 6.23 (td, *J* = 6.7, 1.2 Hz, 1H), 4.36 (q, *J* = 7.1 Hz, 2H), 1.35 (t, *J* = 7.1 Hz, 3H); ^13^C{^1^H} NMR (100 MHz CDCl_3_) δ 163.1, 162.4, 141.0, 137.3, 135.8, 132.9, 132.4, 132.0, 131.7 (q, *J* = 33 Hz), 129.7, 127.7 (q, *J* = 273 Hz), 127.0 (q, *J* = 4 Hz), 126.6 (q, *J* = 4 Hz), 122.1, 107.2, 62.4, 14.2; ^19^F NMR (376 MHz, CDCl_3_) δ −63.2; HRMS (ESI) calcd for C_17_H_15_O_3_F_3_N [M + H]^+^ 338.0999, found 338.0995.

##### *N*-Alkenyl-2-pyridone **3l**

The compound was prepared using the general procedure and obtained as a colorless solid (30 mg, 83%): mp 98–102 °C; chromatography conditions 1:1 EtOAc/Hex to 3:2 EtOAc/Hex; ^1^H NMR (400 MHz, CDCl_3_) δ 7.79 (s, 1H), 7.62 (m, 1H), 7.47 (m, 2H), 7.42 (m, 2H), 6.93 (ddd, *J* = 6.9, 2.0, 0.6 Hz, 1H), 6.68 (dt, *J* = 9.3, 0.9 Hz, 1H), 6.22 (td, *J* = 6.7, 1.2 Hz, 1H), 4.35 (q, *J* = 7.1 Hz, 2H), 1.33 (t, *J* = 7.1 Hz, 3H); ^13^C{^1^H} NMR (100 MHz CDCl_3_) δ 162.9, 162.3, 141.1, 137.1, 135.0, 133.7, 133.5, 133.1, 132.6, 130.0, 122.2, 117.9, 113.6, 107.2, 62.5, 14.2; HRMS (ESI) calcd for C_17_H_15_O_3_N_2_ [M + H]^+^ 295.1077, found 295.1071.

##### *N*-Alkenyl-2-pyridone **3m**

The compound was prepared using the general procedure and obtained as a clear oil (24 mg, 72% yield): chromatography conditions 93:7 EtOAc/MeOH; ^1^H NMR (400 MHz, CDCl_3_) δ 8.58 (d, *J* = 6.0 Hz, 2H), 7.75 (s, 1H), 7.47 (ddd, *J* = 9.4, 6.6, 2.0 Hz, 1H), 7.06 (d, *J* = 6.0 Hz, 2H), 6.92 (m, 1H), 6.68 (d, *J* = 9.3 Hz, 1H), 6.21 (td, *J* = 6.7, 1.2 Hz, 1H), 4.36 (q, *J* = 7.1 Hz, 2H), 1.35 (t, *J* = 7.1 Hz, 3H); ^13^C{^1^H} NMR (100 MHz CDCl_3_) δ 162.7, 162.3, 150.7, 141.1, 139.1, 137.1, 134.7, 134.2, 123.3, 122.0, 107.1, 62.6, 14.2; HRMS (ESI) calcd for C_15_H_15_O_3_N_2_ [M + H]^+^ 271.1077 found 271.1066

##### *N*-Alkenyl-2-pyridone **3n**

The compound was prepared using the general procedure and obtained as a tan solid (27 mg, 82% yield). The reaction performed using 1.5 mmol **1** in 15 mL of toluene afforded 0.355 g (83%) of **3n** (89%): mp 123–125 °C; chromatography conditions 100% EtOAc to 93:7 EtOAc/MeOH; ^1^H NMR (400 MHz, CDCl_3_) δ 8.56 (dd, *J* = 4.8, 1.6 Hz, 1H), 8.52 (d, *J* = 2.1 Hz, 1H), 7.83 (s, 1H), 7.47 (m, 2H), 7.25 (dd, *J* = 8.0, 4.8 Hz, 1H), 6.99 (m, 1H), 6.69 (dt, *J* = 9.4, 1.0 Hz, 1H), 6.24 (td, *J* = 6.7, 1.2 Hz, 1H), 4.36 (q, 7.1 Hz, 2H), 1.35 (t, *J* = 7.1 Hz, 3H); ^13^C{^1^H} NMR (100 MHz CDCl_3_) δ 163.0, 162.3, 151.22, 151.16, 141.0, 137.2, 136.2, 134.2, 132.3, 127.8, 123.9, 122.2, 107.2, 62.4, 14.2; HRMS (ESI) calcd for C_15_H_15_O_3_N_2_ [M + H]^+^ 271.1077, found 271.1077.

##### *N*-Alkenyl-2-pyridone **3o**

The compound was prepared using the general procedure and obtained as a tan solid (31 mg, 94%): mp 94–96 °C; chromatography conditions 3:1 EtOAc/Hex; ^1^H NMR (400 MHz, CDCl_3_) δ 8.57 (ddd, *J* = 4.7, 1.7, 0.9 Hz, 1H), 7.89 (s, 1H), 7.63 (td, *J* = 7.8, 1.8 Hz, 1H), 7.44 (ddd, *J* = 9.3, 6.6, 2.1 Hz, 1H), 7.22 (m, 2H), 7.04 (ddd, *J* = 6.9, 2.0, 0.7 Hz, 1H), 6.65 (dt, *J* = 9.3, 0.9 Hz, 1H), 6.19 (td, *J* = 6.7. 1.2 Hz, 1H), 4.36 (q, *J* = 7.1 Hz, 2H), 1.35 (t, *J* = 7.1 Hz, 3H); ^13^C{^1^H} NMR (100 MHz CDCl_3_) δ 163.4, 162.5, 151.3, 150.3, 140.8, 138.1, 136.71, 136.67, 132.9, 125.4, 124.2, 121.7, 106.3, 62.3, 14.2; HRMS (ESI) HRMS (ESI) calcd for C_15_H_15_O_3_N_2_ [M + H]^+^ 271.1077, found 271.1077.

##### *N*-Alkenyl-2-pyridone **3p**

The compound was prepared using the general procedure and obtained as a clear oil (31 mg, 81%); chromatography conditions 100% EtOAc to 93:7 EtOAc/Hex; ^1^H NMR (400 MHz, CDCl_3_) δ 8.65 (s, 1H), 8.36 (d, *J* = 5.1 Hz, 1H), 7.90 (s, 1H), 7.41 (ddd, *J* = 9.4, 6.6, 2.0 Hz, 1H), 6.99 (d, *J* = 5.1 Hz, 1H), 6.82 (ddd, *J* = 6.9, 2.0, 0.7 Hz, 1H), 6.64 (dt, *J* = 9.3., 0.8 Hz, 1H), 6.12 (td, *J* = 6.7, 1.2 Hz, 1H), 4.39 (q, *J* = 7.1 Hz, 2H) 1.37 (t, *J* = 7.1 Hz, 3H); ^13^C{^1^H} NMR (100 MHz CDCl_3_) δ 162.6, 162.3, 150.1, 148.2, 141.1, 138.4, 136.0, 135.9, 131.52, 131.48, 123.0, 121.7, 107.0, 62.7, 14.2; HRMS (ESI) calcd for C_15_H_14_O_3_N_2_Cl [M + H]^+^ 305.0687, found 305.0687.

##### *N*-Alkenyl-2-pyridone **3q**

The compound was prepared using the general procedure and obtained as a tan solid (21 mg, 88% yield): mp 61–65 °C; chromatography conditions 3:2 EtOAc/Hex to 2:1 EtOAc/Hex; ^1^H NMR (400 MHz, CDCl_3_) δ 7.73 (s, 1H), 7.47 (m, 2H), 7.11 (ddd, *J* = 6.9, 2.0, 0.7 Hz, 1H), 1H), 6.68 (dt, *J* = 9.3, 0.8 Hz, 1H), 6.43 (m, 2H), 6.28 (td, *J* = 6.7. 1.3 Hz, 1H), 4.32 (q, *J* = 7.1 Hz, 2H), 1.32 (t, *J* = 7.1 Hz, 3H); ^13^C{^1^H} NMR (100 MHz CDCl_3_) δ 163.4, 162.1, 148.0, 146.2, 140.6, 138.2, 126.2, 125.7, 122.0, 117.5, 112.8, 106.5, 62.0, 14.3; HRMS (ESI) calcd for C_14_H_14_O_4_N [M + H]^+^ 260.0917, found 260.0916.

##### *N*-Alkenyl-2-pyridone **3r**

The compound was prepared using the general procedure and obtained as a tan oil (13 mg, 38% yield): chromatography conditions 1:1 EtOAc to 2:1 EtOAc/Hex; ^1^H NMR (400 MHz, CDCl_3_) δ 8.03 (s, 1H), 7.45 (m, 1H), 7.40 (d, *J* = 5.1 Hz, 1H), 7.24 (m, 1H), 7.00 (m, 2H), 6.70 (dt, *J* = 9.4, 0.7 Hz, 1H), 6.30 (td, *J* = 6.7, 1.1 Hz, 1H), 4.26 (q, *J* = 7.1 Hz, 2H), 1.26 (t, *J* = 7.1 Hz, 3H); ^13^C{^1^H} NMR (100 MHz CDCl_3_) δ 163.5, 162.1, 141.1, 138.0, 134.8, 134.7, 132.5, 132.1, 127.7, 126.2, 122.6, 107.8, 62.1, 14.3; HRMS (ESI) calcd for C_14_H_14_O_3_NS [M + H]^+^ 276.0689, found 276.0687.

##### *N*-Alkenyl-2-pyridone **3s**

The compound was prepared using the general procedure and obtained as an opaque oil (39 mg, 91% yield). The reaction performed using 1.5 mmol **1** in 15 mL of toluene afforded 0.470 g (90%) of **3s**: chromatography conditions 1:1 EtOAc/Hex; ^1^H NMR (400 MHz, CDCl_3_) δ 7.94 (s, 1H), 7.61 (m 1H), 7.37 (ddd, *J* = 9.4, 6.6, 2.0 Hz, 1H), 7.20 (m, 2H), 7.10 (m, 1H), 6.84 (dd, *J* = 6.9, 2.2 Hz, 1H), 6.63 (d, *J* = 9.3, 1H), 6.06 (td, *J* = 6.7, 1.2 Hz, 1H), 4.37 (q, *J* = 7.1 Hz, 2H), 1.36 (t, *J* = 7.1 Hz, 3H); ^13^C{^1^H} NMR (100 MHz CDCl_3_) δ 163.0, 140.8, 137.6, 136.5, 133.3. 132.9, 132.6, 131.2, 129.9, 127.9, 124.6, 121.5, 106.6, 62.3, 14.2; HRMS (ESI) calcd for C_16_H_15_O_3_NBr [M + H]^+^ 348.0230, found 348.0229.

##### *N*-Alkenyl-2-pyridone **3t**

The compound was prepared using the general procedure and obtained as a clear oil (31 mg, 74% yield): chromatography conditions 1:1 EtOAc/Hex; ^1^H NMR (400 MHz, CDCl_3_) δ 7.97 (s, 1H), 7.43 (d, *J* = 7.5, 2.1 Hz, 1H), 7.37 (ddd, *J* = 9.4, 6.6, 2.1 Hz, 1H), 7.08 (m, 2H), 6.83 (ddd, *J* = 6.9, 2.0, 0.6 Hz, 1H), 6.63 (dt, *J* = 9.3, 0.8 Hz, 1H), 6.08 (td, *J* = 6.7, 1.2 Hz, 1H), 4.38 (q, *J* = 7.1 Hz, 2H), 1.36 (t, *J* = 7.1 Hz, 3H); ^13^C{^1^H} NMR (100 MHz CDCl_3_) δ 162.9, 162.8, 140.9, 137.5, 134.2, 133.7, 133.0, 132.6, 131.6, 127.92, 127.85, 121.6, 106.8, 62.5, 14.2; HRMS (ESI) calcd for C_16_H_14_O_3_NCl_2_ [M + H]^+^ 338.0345, found 338.0343.

##### *N*-Alkenyl-2-pyridone **3u**

The compound was prepared using the general procedure using 2 equiv of **2t** and obtained as a yellow oil (10 mg, 37% yield): chromatography conditions 1:1 EtOAc/Hex; to 3:1 EtOAc/Hex ^1^H NMR (400 MHz, CDCl_3_) δ 7.41 (ddd, *J* = 9.3, 6.6, 2.1 Hz, 1H), 7.13 (t, *J* = 7.6 Hz, 1H), 7.04 (dd, *J* = 6.8, 2.1 Hz, 1H), 6.63 (d, *J* = 9.3 Hz, 1H), 6.22 (td, *J* = 6.1, 1.1 Hz, 1H), 4.27 (q, *J* = 7.1 Hz, 3H), 2.12 (d, *J* = 6.2 Hz, 2H), 1.30 (t, *J* = 7.1 Hz, 4H [presumably 3H with an imbedded impurity]), 1.11 (t, *J* = 7.6 Hz, 3H); ^13^C{^1^H} NMR (100 MHz CDCl_3_) δ 162.9, 162.0, 144.9, 140.3, 137.9, 131.5, 121.9, 105.9, 61.8, 21.5, 14.2, 12.6; HRMS (ESI) calcd for C_12_H_16_O_3_N [M + H]^+^ 222.1125, found 222.1122.

##### *N*-Alkenyl-2-pyridone **3v**

The compound was prepared using the general procedure starting with 1.5 mmol of **1** and 2 equiv of **2u** in 15 mL of toluene and obtained as a yellow oil, (0.141 g, 45% yield): chromatography conditions 1:1 EtOAc/Hex to 3:1 EtOAc/Hex; ^1^H NMR (400 MHz, CDCl_3_) δ 7.43 (m, 1H), 7.24 (q, *J* = 7.2 Hz, 1H), 7.08 (d, *J* = 6.7 Hz, 1H), 6.61 (d, *J* = 9.30 Hz, 1H), 6.26 (t, *J* = 6.69 Hz, 1H) (4.26 (q, *J* = 7.1 Hz, 2H), 1.77 (d, *J* = 7.2, 3H), 1.29 (t, *J* = 7.1 Hz, 3H); ^13^C{^1^H} NMR (100 MHz CDCl_3_) δ 162.4, 161.6, 140.2, 138.3, 137.7, 132.7, 121.3, 105.8, 61.3, 13.9, 13.4; HRMS (ESI) calcd for C_11_H_14_O_3_N [M + H]^+^ 208.0968, found 208.0963.

##### *N*-Alkenyl-2-pyridone **5a**

The compound was prepared using the general procedure and obtained as a yellow solid (21 mg, 54%): mp 126–129 °C; chromatography conditions 1:1 EtOAc Hex to 2:1 EtOAc/Hex; ^1^H NMR (400 MHz, CDCl_3_) δ 8.17 (d, *J* = 8.9 Hz, 2H), 7.85 (s, 1H), 7.40 (d, *J* = 8.6 Hz, 2H), 7.34 (dd, *J* = 9.5, 2.5 Hz, 1H), 6.69 (m, 1H), 6.64 (d, *J* = 9.4 Hz, 1H), 4.37 (q, *J* = 7.1 Hz, 2H), 2.01 (d, *J* = 0.9 Hz, 3H), 1.36 (t, *J* = 7.1 Hz, 3H); ^13^C{^1^H} NMR (100 MHz CDCl_3_) δ 163.0, 161.7, 148.4, 144.1, 138.1, 134.8, 133.9, 133.5, 130.6, 124.2, 121.7, 116.4, 62.6, 17.1, 14.2; HRMS (ESI) calcd for C_17_H_17_O_5_N_2_ [M + H]^+^ 329.1132, found 329.1129.

##### *N*-Alkenyl-2-pyridone **5b**

The compound was prepared using the general procedure and obtained as a yellow oil (27 mg, 53%): chromatography conditions 3:2 Hex/EtOAc to 3:2 EtOAc/Hex; ^1^H NMR (400 MHz, CDCl_3_) δ 8.16 (d, *J* = 8.8 Hz, 2H), 7.82, (s, 1H), 7.35 (d, *J* = 8.9 Hz, 2H), 7.31 (m, 1H), 6.80 (m, 1H), 6.14 (t, *J* = 6.8 Hz, 1H), 4.37 (q, *J* = 7.1 Hz, 2H), 2.20 (s, 3H), 1.35 (t, *J* = 7.1 Hz, 3H); ^13^C{^1^H} NMR (100 MHz CDCl_3_) δ 163.0, 162.8, 148.3, 138.1(2), 138.1(0), 134.3, 134.2, 134.0, 131.3, 130.6, 124.1, 107.1, 62.5, 17.1, 14.2; HRMS (ESI) calcd for C_17_H_17_O_5_N_2_ [M + H]^+^ 329.1132, found 329.1132.

##### *N*-Alkenyl-2-pyridone **5c**

The compound was prepared using the general procedure starting with 1.5 mmol of **4c** and 3 equiv of **2a** in 15 mL of toluene and obtained as a yellow solid (0.432 g, 84%): mp 108–113 °C; chromatography conditions 3:1 EtOAc/Hex; ^1^H NMR (400 MHz, CDCl_3_) δ 8.18 (d, *J* = 8.9 Hz, 2H), 7.86 (s, 1H), 7.43 (d, *J* = 8.7 Hz, 2H), 7.33 (dd, *J* = 10.0, 3.2 Hz, 1H), 6.65 (d, *J* = 10.0 Hz, 1H), 6.39 (d, *J* = 3.1 Hz Hz, 1H), 4.37 (q, *J* = 7.1 Hz, 2H), 3.52 (s, 3H), 1.36 (t, *J* = 7.1 Hz, 3H); ^13^C{^1^H} NMR (100 MHz CDCl_3_) δ 162.6, 160.2, 148.0, 143.9, 137.8, 136.2, 134.6, 133.3, 130.4, 123.9, 122.3, 116.6, 62.3, 56.2, 14.0; HRMS (ESI) calcd for C_17_H_17_O_6_N_2_ [M + H]^+^ 345.1081, found 345.1076.

### Procedures for Mechanistic Probes

#### Scheme 4a

Compounds **1** (40 mg, 0.12 mmol), **2a** (19 mg, 0.12 mmol), and activated 4 Å MS (0.100 g) were added to a 25 mL two-neck flask charged with a stir bar. The solids were dissolved/suspended in toluene and cooled to 0 °C for 30 min while stirring. DBU (40 μL, 0.26 mmol) was added via syringe. Upon completion of the reaction (determined by the consumption of aldehyde by TLC), contents were transferred to an RBF, rinsed with DCM, and concentrated via rotary evaporation. The residual material was purified via flash column chromatography. Less than 1 mg of the desired product was isolated.

#### Scheme 4b

Compound **1** (40 mg, 0.12 mmol) was added to a 25 mL two-neck flask charged with a stir bar. The solid was suspended in toluene, and the reaction was cooled to 0 °C for 30 min while stirring. DBU (40 μL, 0.26 mmol) was added via syringe. After 20 min, water (13 μL, 0.6 mmol) was added, and the reaction was maintained overnight (∼16 h). After this time, the reaction contents were transferred to an RBF, rinsed with DCM, and concentrated via rotary evaporation. Analysis of the residue via ^1^H NMR revealed only trace amounts of the expected pyridone product **6**.

#### Scheme 4c

Compounds **1** (40 mg, 0.12 mmol) and **2a** (19 mg, 0.12 mmol) were added to a 25 mL two-neck flask charged with a stir bar. Toluene (1.2 mL) was added, and the reaction was cooled to 0 °C for 30 min while stirring. DBU (40 μL, 0.26 mmol, 2.1 equiv) and water (50 °CL, 2.4 mmol) were added sequentially via syringe, and the reaction was maintained overnight (∼16 h). After this time, reaction contents were transferred to an RBF, rinsed with DCM, and concentrated via rotary evaporation. A trace amount of product was indicated by TLC, but no product could be isolated after flash column chromatography.

### Synthetic Manipulations of *N*-Alkenyl Pyridones

#### 2-Pyridone **7**

Compound **3a** (35 mg, 0.111 mmol) and SnCl_2_ (84 mg, 0.445 mmol) were added to a 25 mL RBF charged with a stir bar. Ethanol (1.1 mL) and 1 M aqueous HCl (0.1 mL) were added, and the reaction was heated in an 80 °C oil bath for 3 h. After cooling to rt, that reaction mixture was concentrated via rotary evaporation. Saturated aqueous Na_2_CO_3_ solution (5 mL) and EtOAc (10 mL) were added to the residue, and the layers were separated. The aqueous phase was extracted with additional EtOAc (2 × 10 mL), and the combined organic layer was dried over anhydrous Na_2_SO_4_, filtered, concentrated, and purified by flash column chromatography (3:1 EtOAc/Hex to 100% EtOAc) to afford **7** as a yellow oil (18 mg, 56% yield): ^1^H NMR (400 MHz, CDCl_3_) δ 7.72 (s, 1H), 7.46 (ddd, *J* = 9.3, 6.6, 2.1 Hz, 1H), 7.06 (dd, *J* = 6.8, 1.6 Hz, 1H), 6.97 (d, *J* = 8.6 Hz, 2H), 6.70 (d, *J* = 9.3 Hz, 1H), 6.52 (d, *J* = 8.7 Hz, 2H), 6.24 (td, *J* = 6.7, 1.2 Hz, 1H), 4.30 (q, *J* = 7.1 Hz, 2H), 4.08 (s, 2H), 1.31 (t, *J* = 7.1 Hz, 3H); ^13^C{^1^H} NMR (100 MHz CDCl_3_) δ 164.2, 162.5, 149.3, 140.7, 138.5, 138.2, 132.5, 125.6, 122.0, 121.3, 114.8, 106.9, 61.7, 14.3; HRMS (ESI) calcd for C_16_H_17_O_3_N_2_ [M + H]^+^ 285.1234, found 285.1225.

#### 2-Pyridone **8**

Under Ar, Cu(OAc)_2_ (6 mg, 0.033 mmol) and 1,2-bis(diphenylphosphino)benzene (2 mg, 0.003 mmol) were added to an oven-dried 25 mL two-neck flask charged with a stir bar and equipped with a reflux condenser. The solids were dissolved in toluene (2 mL), and *tert*-butyl alcohol (0.560 mL) was added. The mixture was stirred for 15 min to allow ligation, and then polymethylhydrosiloxane (PMHS, 0.470 mL) was slowly added with continued stirring. The solution changed from blue to yellow over 10 min. Pyridone **3f** (50 mg, 0.167 mmol) was dissolved in toluene (0.5 mL), and the solution was added to the reaction, resulting in the formation of a brown reaction solution. The reaction was heated in a 60 °C oil bath, and the reaction progress was monitored by ^1^H NMR (disappearance of a vinylic hydrogen signal at ∼7.9 ppm). Upon completion, the reaction was allowed to cool to room temperature, saturated aqueous NH_4_Cl solution (5 mL) and saturated aqueous Na_2_CO_3_ solution (5 mL) were added, and stirring continued for 20 min. EtOAc (5 mL) was then added, and the mixture was transferred to a separatory funnel. The layers were separated, and the aqueous phase was extracted with EtOAc (1 × 5 mL, 2 × 10 mL, total 25 mL). The combined organic layer was dried over anhydrous Na_2_SO_4_, filtered, and concentrated *in vacuo*. The residue was purified via flash column chromatography (1:1 EtOAc/Hex to 3:1 EtOAc/Hex) to afford **8** as a clear oil (43 mg, 86%): ^1^H NMR (400 MHz, CDCl_3_) δ 7.29 (m, 1H), 7.06 (dd, *J* = 6.9, 1.8 Hz, 1H), 7.01 (d, *J* = 8.5 Hz, 2H), 6.77 (d, *J* = 8.5 Hz, 2H), 6.52 (d, *J* = 9.2 Hz, 1H), 6.08 (t, *J* = 6.7 Hz, 1H), 5.43 (dd, *J* = 9.6, 5.6 Hz, 1H), 4.24 (q, *J* = 7.14 Hz, 2H), 3.75 (s, 3H), 3.46 (dd, *J* = 14.4, 5.6 Hz, 1H), 3.29 (dd, *J* = 14.4, 9.7 Hz, 1H), 1.26 (t, *J* = 7.14 Hz, 3H); ^13^C{^1^H} NMR (100 MHz CDCl_3_) δ 169.6, 162.2, 158.6, 139.6, 136.5, 130.2, 128.0, 120.7, 114.1, 105.7, 61.9, 61.1, 55.2, 35.6, 14.1; HRMS (ESI) calcd for C_17_H_20_O_4_N [M + H]^+^ 302.1387 found 302.1377.

#### 2-Pyridone **9**

Under Ar, **3s** (50 mg, 0.144 mmol), KOAc (28 mg, 0.287 mmol), and Pd(TFA)_2_ (5 mg, 0.014 mmol) were combined in a 25 mL two-neck flask charged with a stir bar. The materials were dissolved in DMAc (1 mL), and the reaction was heated in a 100 °C oil bath overnight (∼16 h). The reaction progress was monitored by ^1^H NMR because the product and starting material have similar TLC *R*_*f*_ values (although product **9** is fluorescent, while **3s** is not). Upon completion, the reaction was allowed to cool to room temperature, followed by the addition of EtOAc (10 mL) and H_2_O (5 mL). The layers were separated, and the organic phase was washed with H_2_O (3 × 5 mL) to remove DMAc. The organic phase was then dried over anhydrous Na_2_SO_4_, filtered, and concentrated. The residue was purified via flash column chromatography (2:1 EtOAc/Hex) to afford **9** as a vibrant yellow-orange oil (21 mg, 55%): ^1^H NMR (400 MHz, CDCl_3_) δ 8.20 (m, 1H), 7.67 (dd, *J* = 8.9, 7.6 Hz, 1H), 7.60 (m, 3H), 7.32 (d, *J* = 7.5 Hz, 1H), 7.13 (s, 1H), 6.68 (d, *J* = 9.0 Hz, 1H), 4.49 (q, *J* = 7.2 Hz, 2H), 1.44 (q, *J* = 7.2 Hz, 3H); ^13^C{^1^H} NMR (100 MHz CDCl_3_) δ 164.7, 160.3, 141.1, 138.7, 130.9, 130.3, 129.8, 129.0, 128.0, 127.1, 123.8, 117.4, 114.6, 100.4, 62.1, 14.2; HRMS (ESI) calcd for C_16_H_14_O_3_N [M + H]^+^ 268.0968, found 268.0962.

## Supporting Information Available

X-ray crystallographic data and NMR spectra (PDF)

## Accession Codes

CCDC 2068873 contains the supplementary crystallographic data for this paper. These data can be obtained free of charge viawww.ccdc.cam.ac.uk/data_request/cif, or by emailing data_request@ccdc.cam.ac.uk, or by contacting The Cambridge Crystallographic Data Centre, 12 Union Road, Cambridge CB2 1EZ, UK; fax: + 44 1223 336033.
