# Supplementary material for: A Mild and Highly Diastereoselective Preparation of N-Alkenyl-2-Pyridones via 2-Halopyridinium Salts and Aldehydes
Source: J Org Chem. 2021 Aug 31;86(18):13134–42. doi: 10.1021/acs.joc.1c01566 (PMC8453634; doi:10.1021/acs.joc.1c01566)
Supplement: Supplementary file 1 — jo1c01566_si_001.pdf [file jo1c01566_si_001.pdf]

Supporting Information for:

A Mild and Highly Diastereoselective Preparation of *N*-Alkenyl-2-Pyridones via 2-Halopyridinium Salts and Aldehydes

Grant N. Shivers and F. Christopher Pigge*

Department of Chemistry, University of Iowa, Iowa City, Iowa 52242-1294

Table of Contents:

S2-S4 X-Crystallography Data

S5-S62 NMR Spectra

Crystallographic Data

Single-crystal X-ray diffraction data were collected on a Bruker® Nonius-Kappa© APEX II CCD diffractometer equipped with an Oxford Cryosystems® 700 series cold N_2 gas stream cooling system. Data were collected at room temperature (298.15 K) using graphite-monochromated MoKα radiation (λ = 0.71073 Å). Crystals were mounted in Paratone® oil on a MiTeGen© magnetic mount. Data collection strategies for ensuring maximum data redundancy and completeness were calculated using the Bruker® Apex II™ software suite. Data collection, initial indexing, frame integration, Lorentz-polarization corrections and final cell parameter calculations were likewise accomplished using the Apex II software suite. Multi-scan absorption corrections were performed using SADABS.[1] Structure solution and refinement were accomplished using SHELXT[2] and SHELXL,[3] respectively, within the Olex2[4] graphical user interface. Space groups were unambiguously verified using the PLATON©[5] executable. All non-hydrogen atoms were refined anisotropically. All hydrogen atoms were attached *via* a riding model at calculated positions using suitable HFIX commands. The occupancies of the major and minor positions for the disordered C=C core within (cat)·(3,3'-bpe) converged to their respective ratios after each was identified in the difference map and freely refined. Figures of all structures were rendered in the CCDC Mercury[6] software suite

Crystal structure of Ethyl (*Z*)-2-(3-cyanophenyl)-3-(2-oxopyridin-1(2*H*)-yl)acrylate (3l). Compound **3l** crystallizes from $CDCl_3$ as colorless plates in the monoclinic space group $P2_1/c$. The asymmetric unit consists of one full molecule of **3l** (**Figure S1a**). The pyridone ring lies significantly twisted from the plane of the phenyl ring (Θ ~ 78°), presumably to circumvent $A^{1,3}$ strain arising from the (*Z*)-configuration of the alkene (**Figure S1a**); as a consequence, the two rings are broken from conjugation with one another. The molecule self-assembles primarily via C-H···O foSrces between neighboring pyridone rings (*d*(O1···H4) ~ 2.5 Å; Θ(C4-H4-O1) ~ 137°) to form linear chains, characterized by an ABAB repeat motif, along the crystallographic *c*-axis (**Figure 1b**). Adjacent chains interact primarily via C-H···O forces between the pyridone O-atom and a methyl H-atom of the ethyl ester (*d*(O1···H9C) ~ 2.7 Å; Θ(C1-O1-H9C) ~ 100°) to generate planar sheets along the crystallographic *bc*-plane (**Figure S1c**).

Table S1. Crystal data and structure refinement for **3l** (CCDC 2068873).

Identification code	**3l** (CCDC 2068873)
Empirical formula	$C_{17}H_{14}N_2O_3$
Formula weight	294.30
Temperature/K	298.15
Crystal system	monoclinic
Space group	$P2_1/c$
a/Å	12.689(5)
b/Å	8.739(3)
c/Å	13.692(4)
α/°	90
β/°	102.695(9)
γ/°	90
Volume/Å^3	1481.2(9)

Z	4
ρ_{calc}g/cm^3	1.320
μ/mm^{-1}	0.092
F(000)	616.0
Crystal size/mm^3	0.297 × 0.21 × 0.123
Radiation	MoKα (λ = 0.71073)
2Θ range for data collection/°	5.57 to 50
Index ranges	$-15 \leq h \leq 15$, $-10 \leq k \leq 10$, $-16 \leq l \leq 16$
Reflections collected	40024
Independent reflections	2617 [R_{int} = 0.0370, R_{sigma} = 0.0215]
Data/restraints/parameters	2617/0/200
Goodness-of-fit on F^2	1.029
Final R indexes [I>=2σ (I)]	R_1 = 0.0476, wR_2 = 0.1217
Final R indexes [all data]	R_1 = 0.0510, wR_2 = 0.1241
Largest diff. peak/hole / e Å^{-3}	0.49/-0.31

Figure S1a.

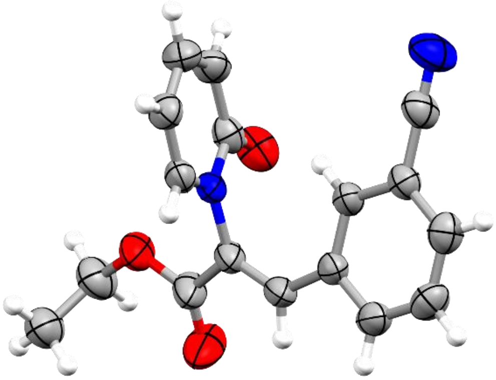

Figure S1b.

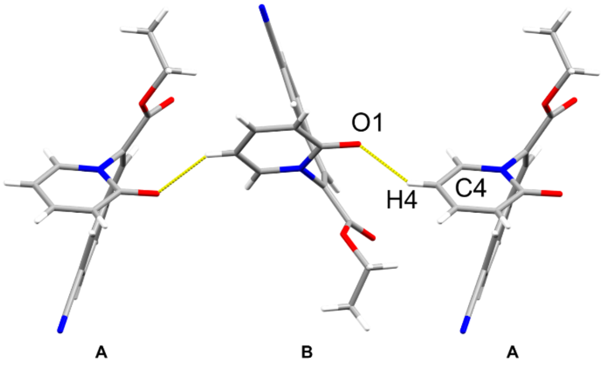

Figure S1c.

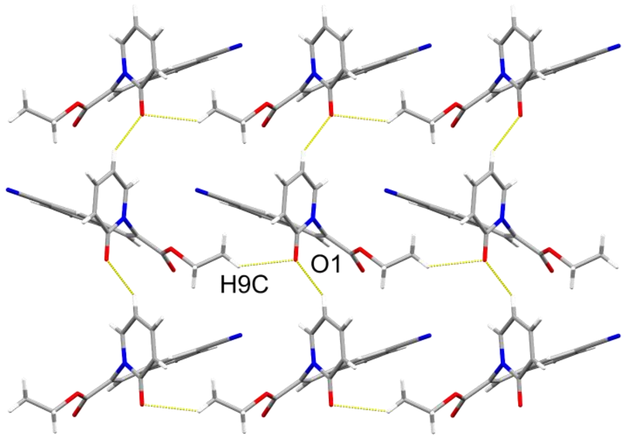

Figure S1. Perspectives of **3l**: a) asymmetric unit (thermal ellipsoids shown at 50% probability); b) linear chains illustrating ABAB repeat motif; c) planar sheets (view along *a*).

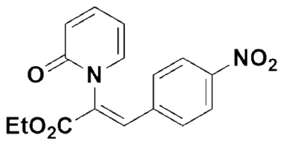

3a

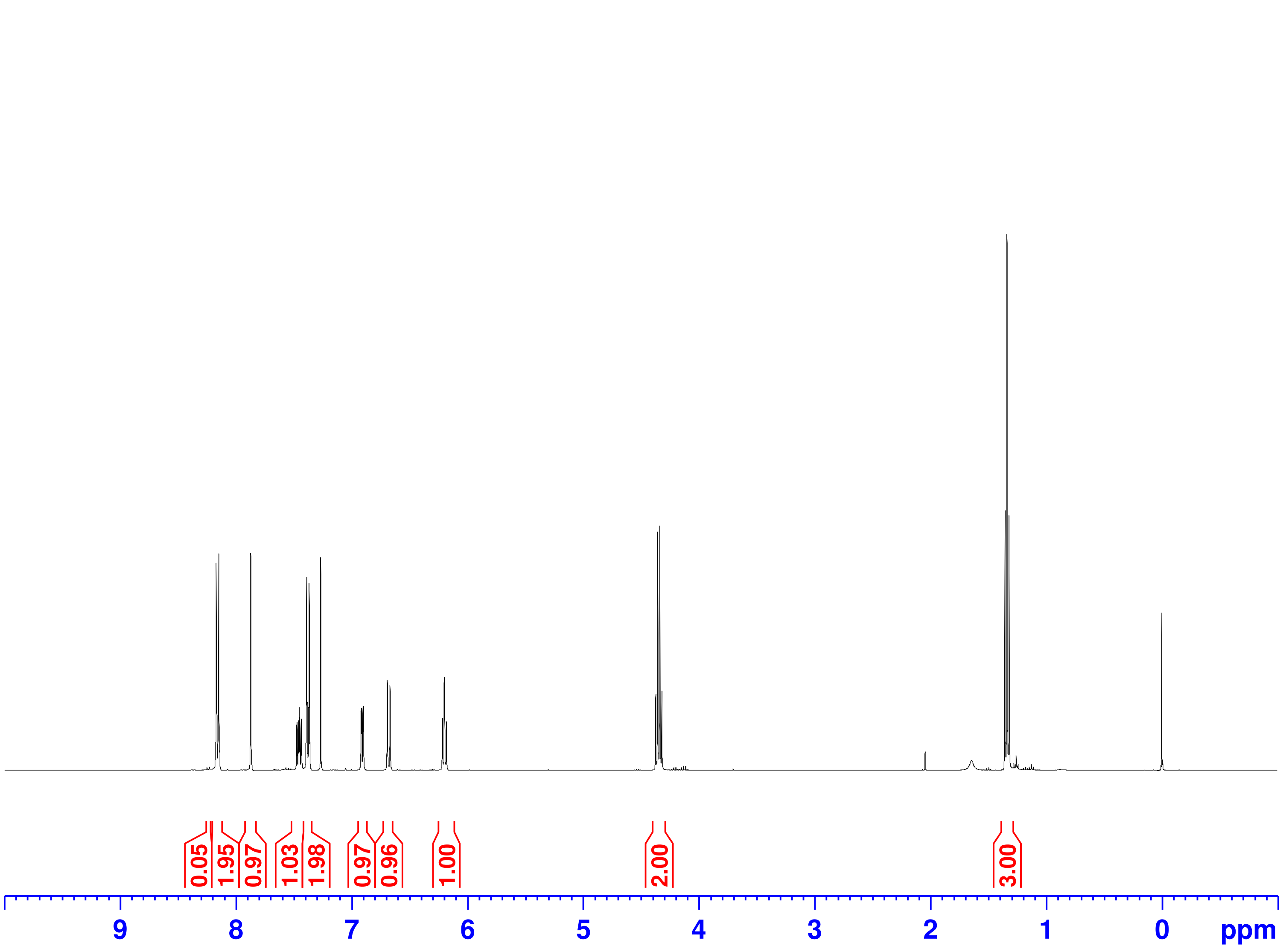

Current Data Parameters
NAME 1H gns-11-082
EXPNO 1
PROCNO 1

F2 - Acquisition Parameters
Date_ 20210120
Time 17.21 h
INSTRUM spect
PROBHD Z104450_0192 (
PULPROG zg30
TD 65536
SOLVENT CDCl3
NS 16
DS 2
SWH 8012.820 Hz
FIDRES 0.244532 Hz
AQ 4.0894465 sec
RG 456
DW 62.400 usec
DE 16.92 usec
TE 298.0 K
D1 1.00000000 sec
TD0 1
SFO1 400.1324708 MHz
NUC1 1H
P0 5.00 usec
P1 15.00 usec
PLW1 8.47000027 W

F2 - Processing parameters
SI 65536
SF 400.1300070 MHz
WDW EM
SSB 0
LB 0.30 Hz
GB 0
PC 1.00

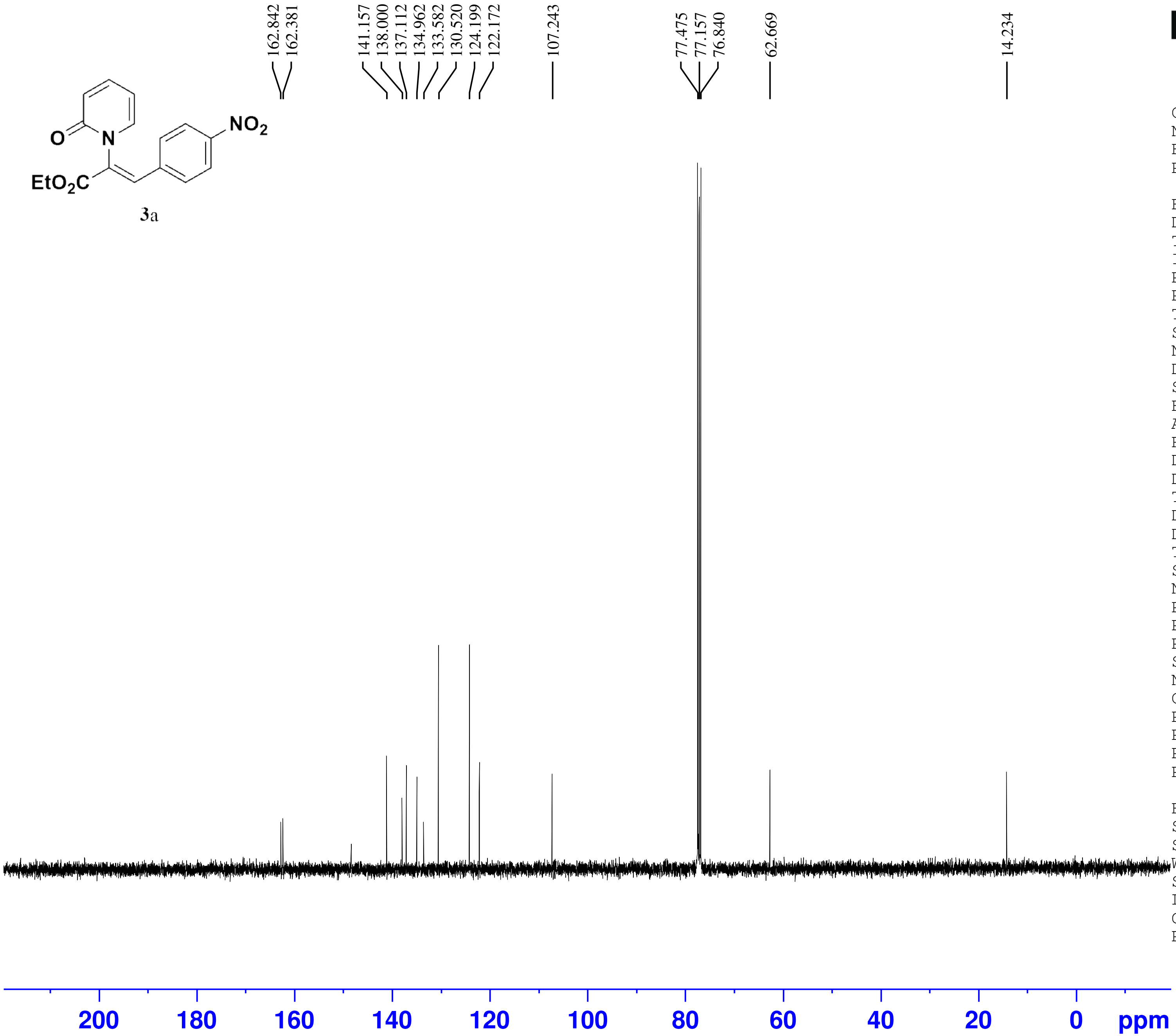

Current Data Parameters

NAME	13C gns-11-082
EXPNO	1
PROCNO	1

F2 - Acquisition Parameters

Date_	20210120
Time	17.34 h
INSTRUM	spect
PROBHD	Z104450_0192 (
PULPROG	zgpg30
TD	65536
SOLVENT	CDCl3
NS	179
DS	2
SWH	24038.461 Hz
FIDRES	0.733596 Hz
AQ	1.3631488 sec
RG	203
DW	20.800 usec
DE	6.50 usec
TE	298.0 K
D1	2.00000000 sec
D11	0.03000000 sec
TD0	1
SFO1	100.6228298 MHz
NUC1	13C
P0	3.28 usec
P1	9.85 usec
PLW1	28.63999939 W
SFO2	400.1316005 MHz
NUC2	1H
CPDPRG[2	waltz65
PCPD2	90.00 usec
PLW2	8.47000027 W
PLW12	0.23528001 W
PLW13	0.11834000 W

F2 - Processing parameters

SI	32768
SF	100.6127559 MHz
WDW	EM
SSB	0
LB	1.00 Hz
GB	0
PC	1.40

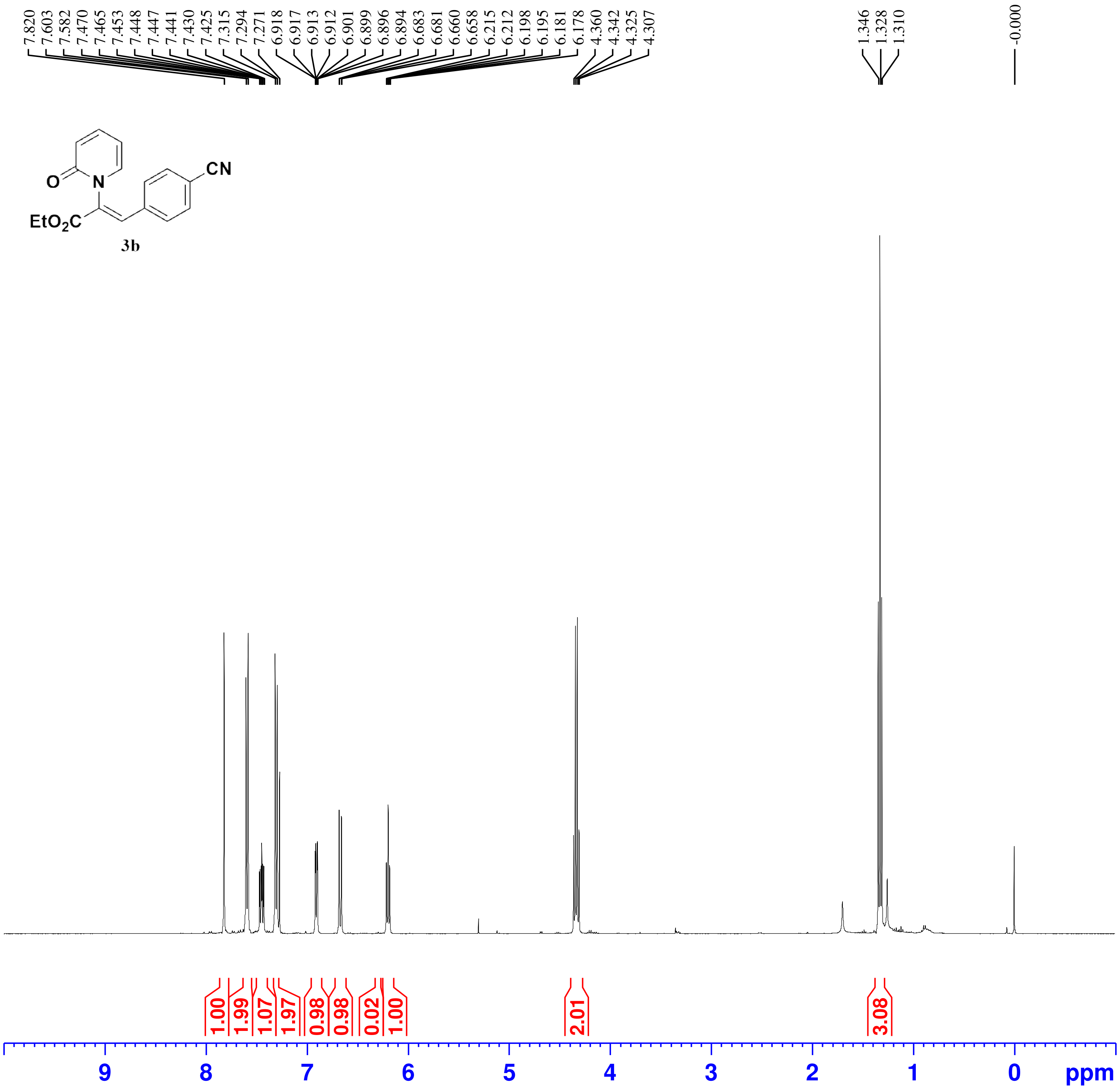

Current Data Parameters
NAME 1H gns-11-106 2
EXPNO 1
PROCNO 1

F2 - Acquisition Parameters
Date_ 20210114
Time 18.54 h
INSTRUM spect
PROBHD Z104450_0192 (
PULPROG zg30
TD 65536
SOLVENT CDCl3
NS 16
DS 2
SWH 8012.820 Hz
FIDRES 0.244532 Hz
AQ 4.0894465 sec
RG 203
DW 62.400 usec
DE 16.92 usec
TE 298.0 K
D1 1.00000000 sec
TD0 1
SFO1 400.1324708 MHz
NUC1 1H
P0 5.00 usec
P1 15.00 usec
PLW1 8.47000027 W

F2 - Processing parameters
SI 65536
SF 400.1300051 MHz
WDW EM
SSB 0
LB 0.30 Hz
GB 0
PC 1.00

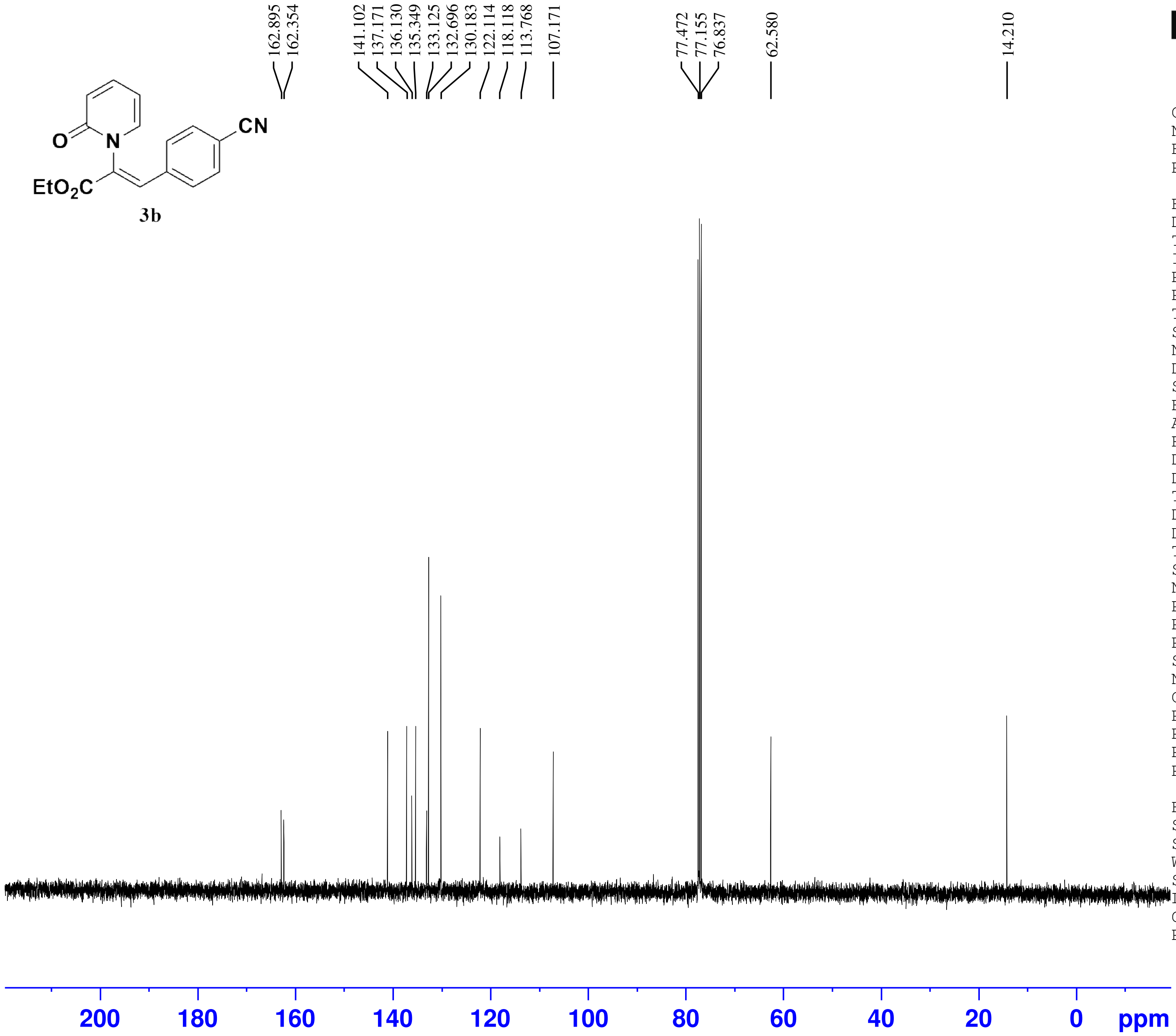


```
Current Data Parameters
NAME       13C gns-11-106 2
EXPNO                    1
PROCNO                   1

F2 - Acquisition Parameters
Date_             20210114
Time                 19.02 h
INSTRUM              spect
PROBHD     Z104450_0192 (
PULPROG            zgpg30
TD                  65536
SOLVENT             CDCl3
NS                    100
DS                      2
SWH             24038.461 Hz
FIDRES           0.733596 Hz
AQ              1.3631488 sec
RG                    203
DW                 20.800 usec
DE                   6.50 usec
TE                  298.1 K
D1             2.00000000 sec
D11            0.03000000 sec
TD0                     1
SFO1          100.6228298 MHz
NUC1                  13C
P0                   3.28 usec
P1                   9.85 usec
PLW1          28.63999939 W
SFO2          400.1316005 MHz
NUC2                   1H
CPDPRG[2          waltz65
PCPD2               90.00 usec
PLW2           8.47000027 W
PLW12          0.23528001 W
PLW13          0.11834000 W

F2 - Processing parameters
SI                  32768
SF            100.6127579 MHz
WDW                    EM
SSB        0
LB                   1.00 Hz
GB         0
PC                   1.40
```

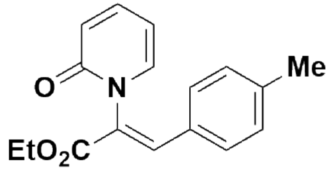

3c

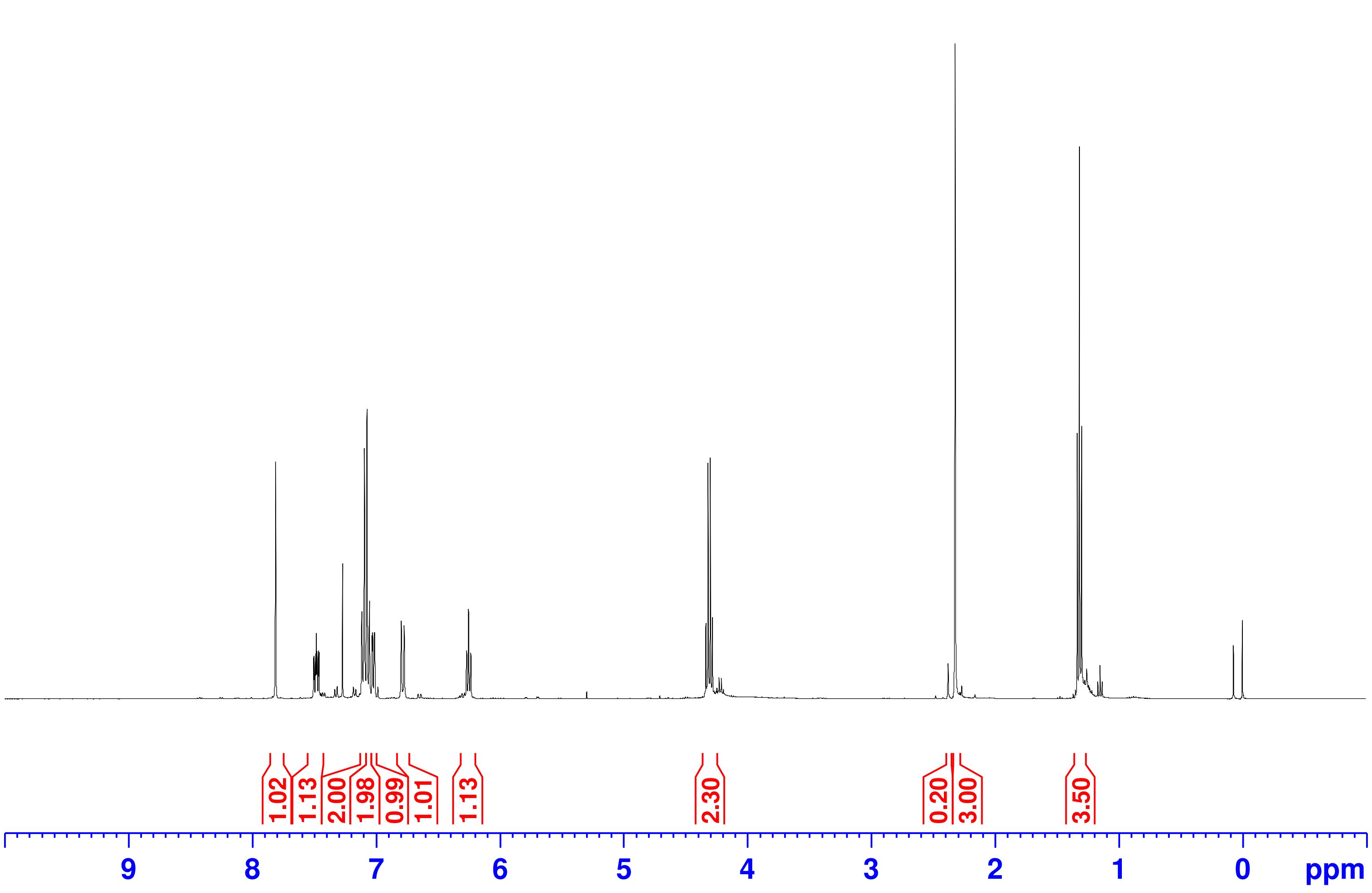


```
Current Data Parameters
NAME       1H gns-11-126
EXPNO                  1
PROCNO                 1

F2 - Acquisition Parameters
Date_           20210114
Time               15.55 h
INSTRUM            spect
PROBHD   Z104450_0192 (
PULPROG             zg30
TD                 65536
SOLVENT            CDCl3
NS                    16
DS                     2
SWH             8012.820 Hz
FIDRES          0.244532 Hz
AQ             4.0894465 sec
RG                   161
DW                62.400 usec
DE                 16.92 usec
TE                 298.0 K
D1            1.00000000 sec
TD0                    1
SFO1         400.1324708 MHz
NUC1                  1H
P0                  5.00 usec
P1                 15.00 usec
PLW1          8.47000027 W

F2 - Processing parameters
SI                 65536
SF           400.1300056 MHz
WDW                   EM
SSB      0
LB                  0.30 Hz
GB       0
PC                  1.00
```

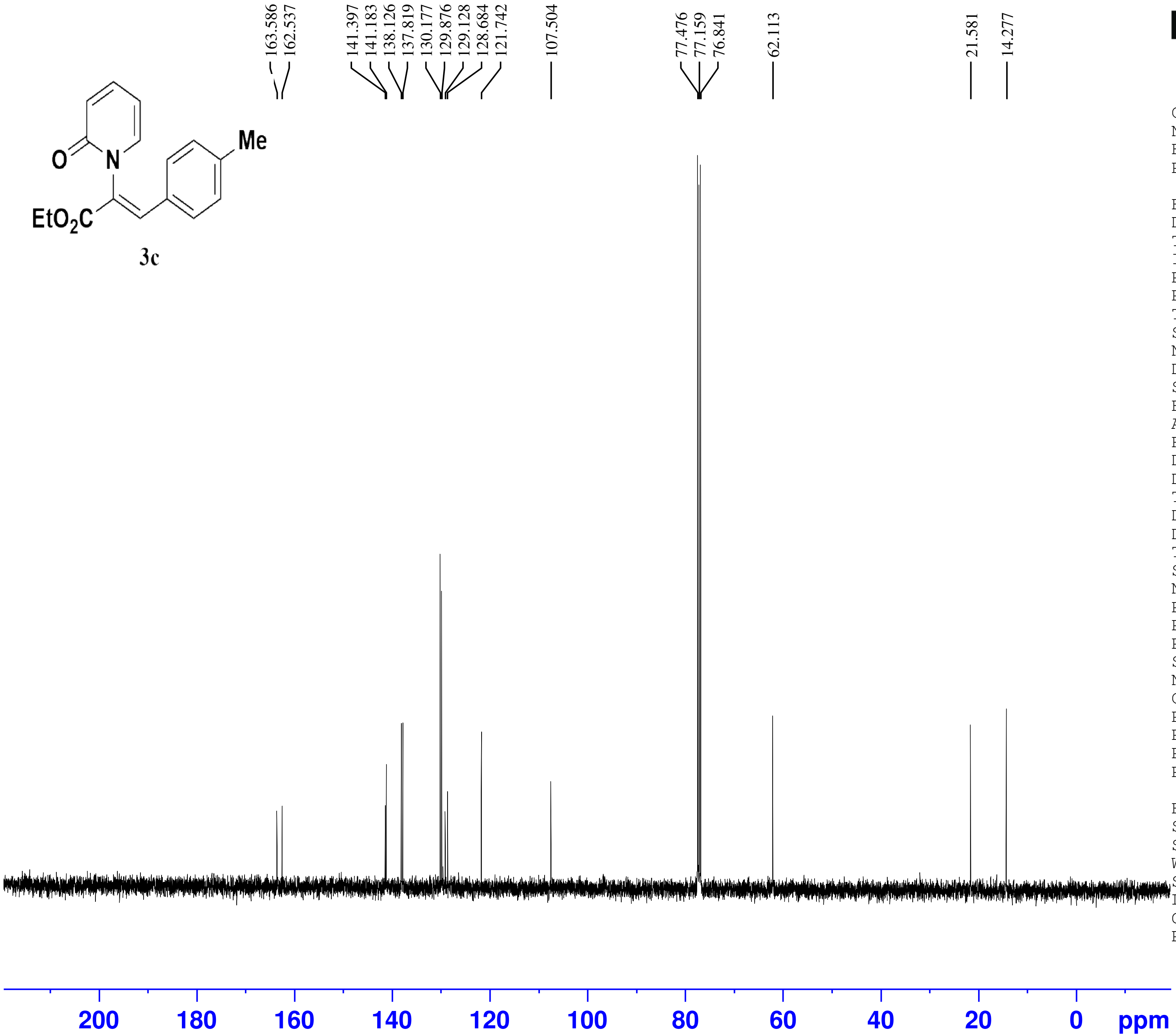

BRUKER

```
Current Data Parameters
NAME        13C gns-11-126
EXPNO                    1
PROCNO                   1

F2 - Acquisition Parameters
Date_             20210114
Time                 16.03 h
INSTRUM              spect
PROBHD    Z104450_0192 (
PULPROG            zgpg30
TD                   65536
SOLVENT              CDCl3
NS                     100
DS                       2
SWH              24038.461 Hz
FIDRES            0.733596 Hz
AQ               1.3631488 sec
RG                     203
DW                  20.800 usec
DE                    6.50 usec
TE                   298.3 K
D1              2.00000000 sec
D11             0.03000000 sec
TD0                      1
SFO1           100.6228298 MHz
NUC1                   13C
P0                    3.28 usec
P1                    9.85 usec
PLW1           28.63999939 W
SFO2           400.1316005 MHz
NUC2                    1H
CPDPRG[2           waltz65
PCPD2                90.00 usec
PLW2            8.47000027 W
PLW12           0.23528001 W
PLW13           0.11834000 W

F2 - Processing parameters
SI                   32768
SF             100.6127580 MHz
WDW                     EM
SSB      0
LB                    1.00 Hz
GB       0
PC                    1.40
```

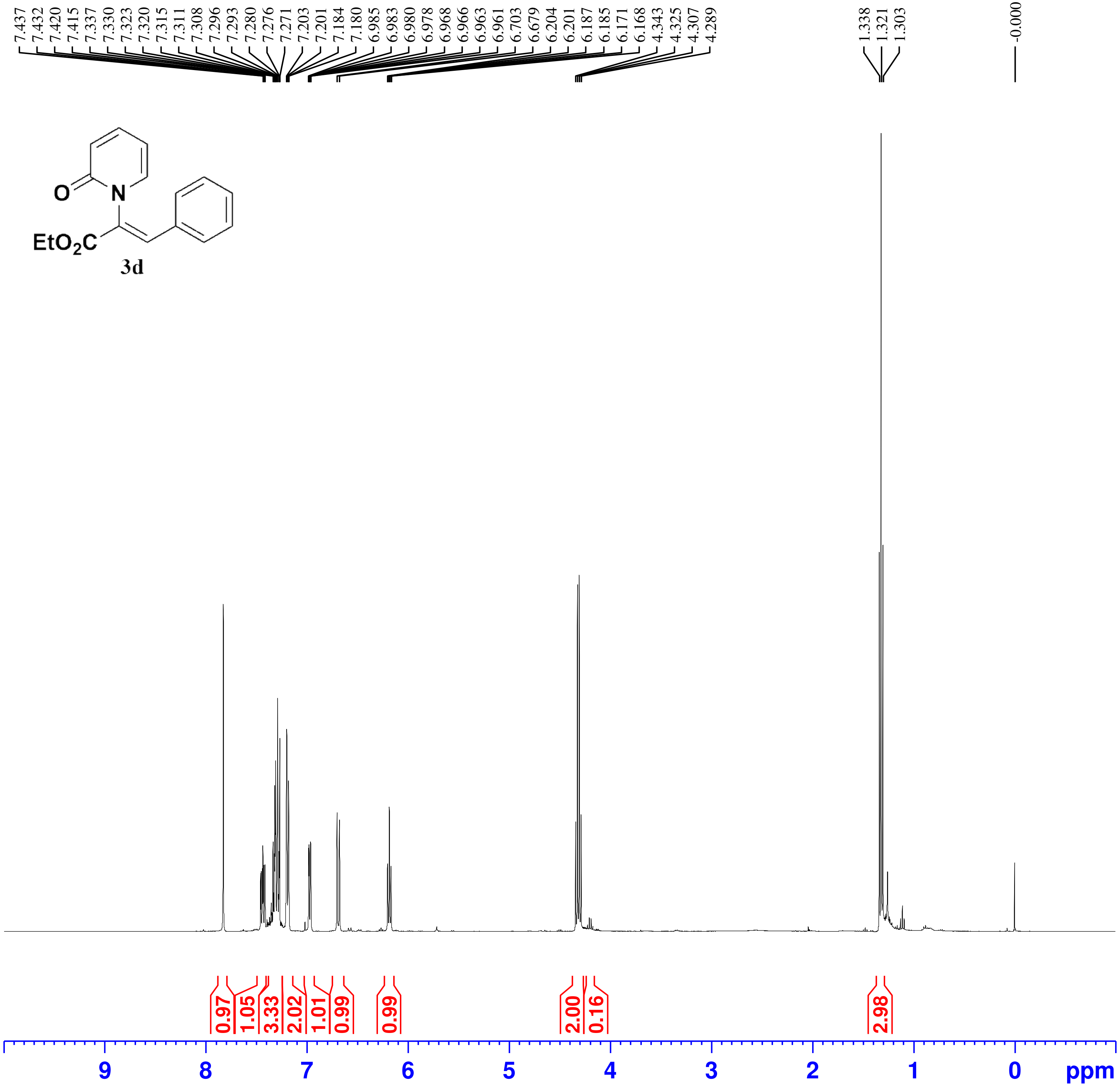


```
Current Data Parameters
NAME        1H gns-11-166
EXPNO                   1
PROCNO                  1

F2 - Acquisition Parameters
Date_            20210203
Time                17.03 h
INSTRUM             spect
PROBHD   Z104450_0192 (
PULPROG              zg30
TD                  65536
SOLVENT             CDCl3
NS                     16
DS                      2
SWH              8012.820 Hz
FIDRES           0.244532 Hz
AQ              4.0894465 sec
RG                    144
DW                 62.400 usec
DE                  16.92 usec
TE                  298.0 K
D1             1.00000000 sec
TD0                     1
SFO1          400.1324708 MHz
NUC1                   1H
P0                   5.00 usec
P1                  15.00 usec
PLW1           8.47000027 W

F2 - Processing parameters
SI                  65536
SF            400.1300053 MHz
WDW                    EM
SSB      0
LB                   0.30 Hz
GB       0
PC                   1.00
```

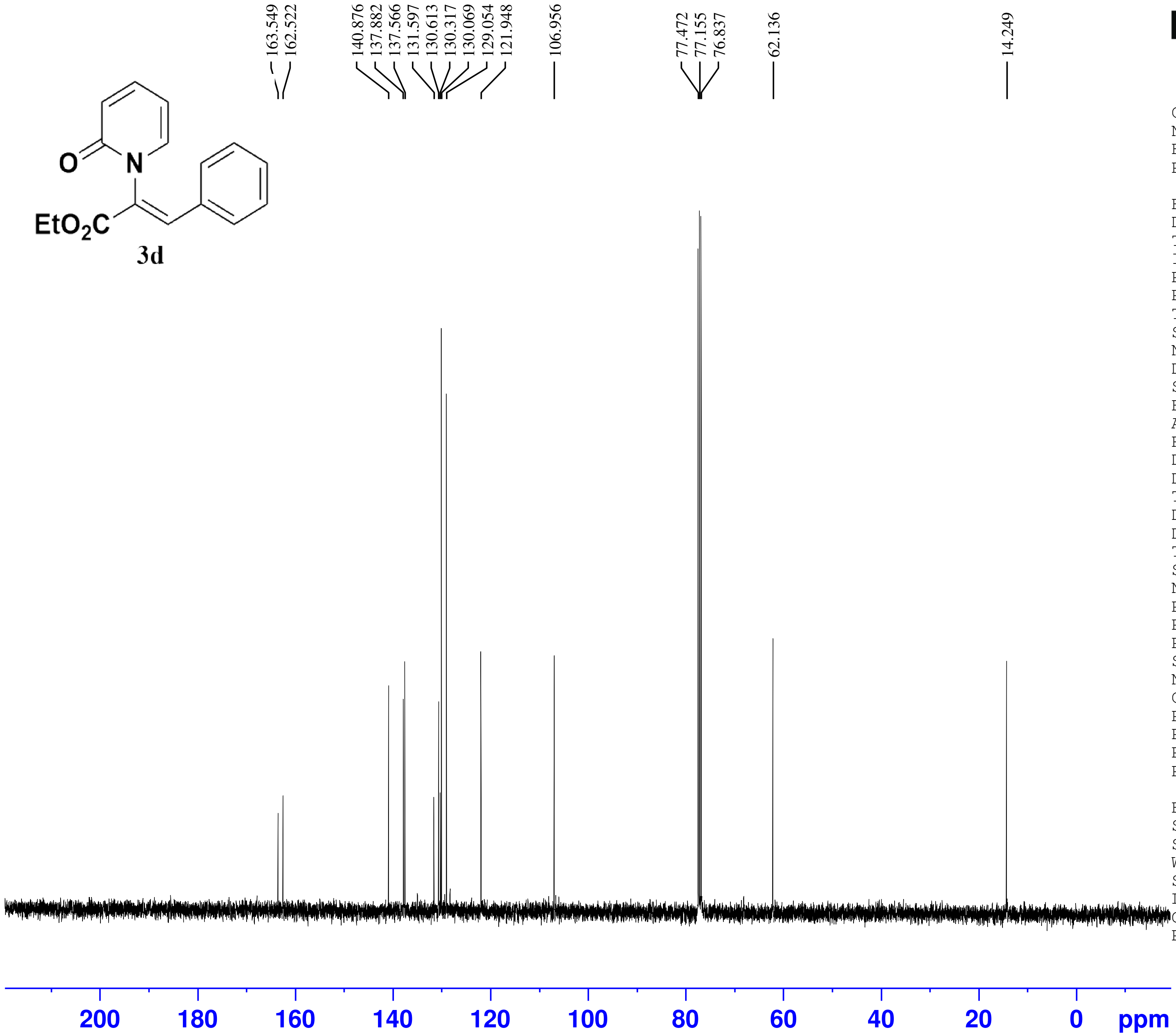

Current Data Parameters
NAME 13C gns-11-166
EXPNO 1
PROCNO 1

F2 - Acquisition Parameters
Date_ 20210203
Time 17.12 h
INSTRUM spect
PROBHD Z104450_0192 (
PULPROG zgpg30
TD 65536
SOLVENT CDCl3
NS 100
DS 2
SWH 24038.461 Hz
FIDRES 0.733596 Hz
AQ 1.3631488 sec
RG 161
DW 20.800 usec
DE 6.50 usec
TE 298.4 K
D1 2.00000000 sec
D11 0.03000000 sec
TD0 1
SFO1 100.6228298 MHz
NUC1 13C
P0 3.28 usec
P1 9.85 usec
PLW1 28.63999939 W
SFO2 400.1316005 MHz
NUC2 1H
CPDPRG[2 waltz65
PCPD2 90.00 usec
PLW2 8.47000027 W
PLW12 0.23528001 W
PLW13 0.11834000 W

F2 - Processing parameters
SI 32768
SF 100.6127594 MHz
WDW EM
SSB 0
LB 1.00 Hz
GB 0
PC 1.40

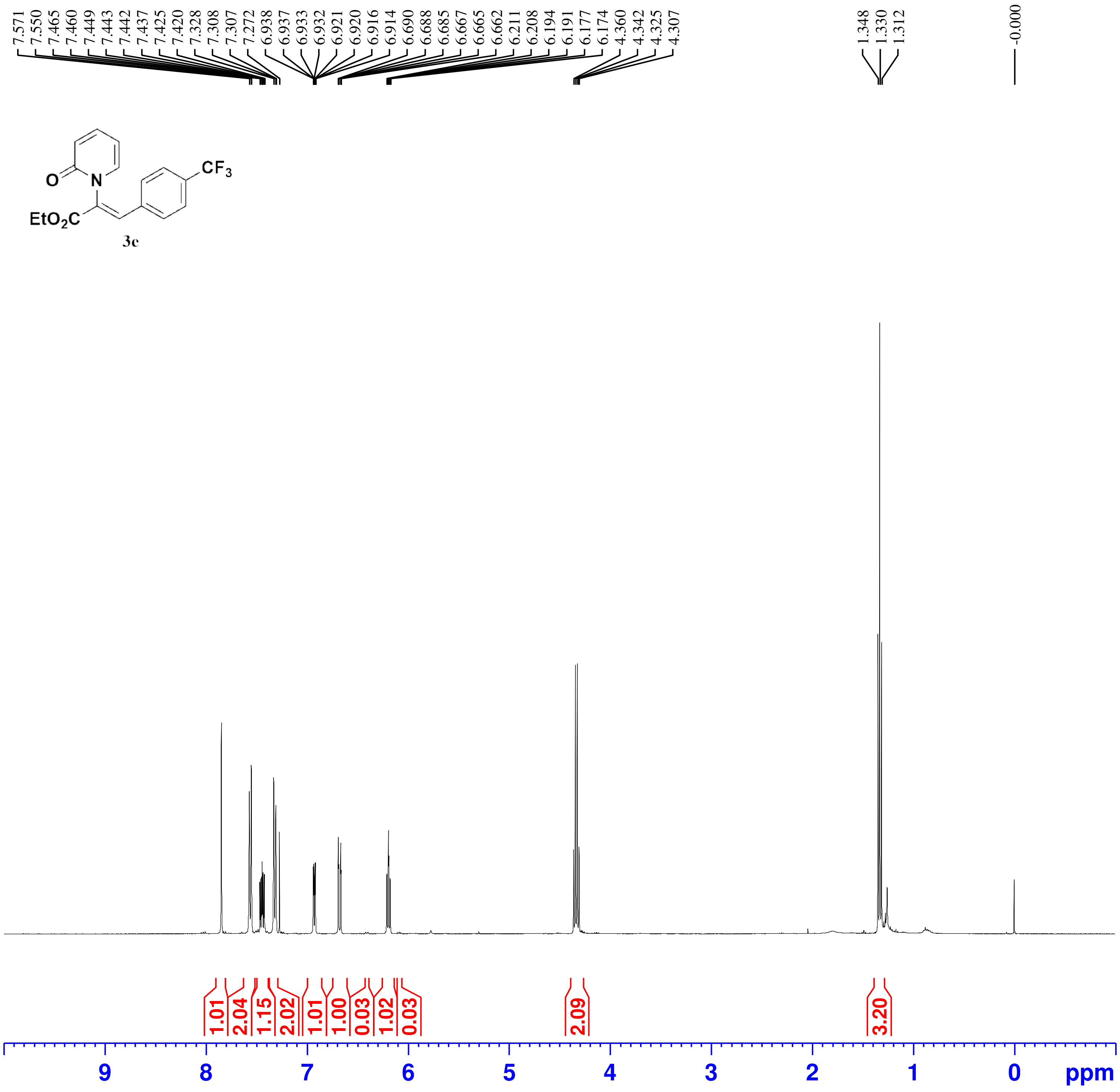

BRUKER

Current Data Parameters
NAME 1H gns-11-092
EXPNO 1
PROCNO 1

F2 - Acquisition Parameters
Date_ 20201221
Time 16.59 h
INSTRUM spect
PROBHD Z104450_0192 (
PULPROG zg30
TD 65536
SOLVENT CDCl3
NS 16
DS 2
SWH 8012.820 Hz
FIDRES 0.244532 Hz
AQ 4.0894465 sec
RG 161
DW 62.400 usec
DE 16.92 usec
TE 298.0 K
D1 1.00000000 sec
TD0 1
SFO1 400.1324708 MHz
NUC1 1H
P0 5.00 usec
P1 15.00 usec
PLW1 8.47000027 W

F2 - Processing parameters
SI 65536
SF 400.1300047 MHz
WDW EM
SSB 0
LB 0.30 Hz
GB 0
PC 1.00

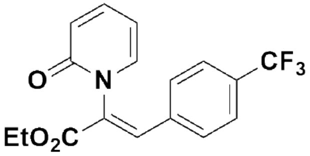

3c

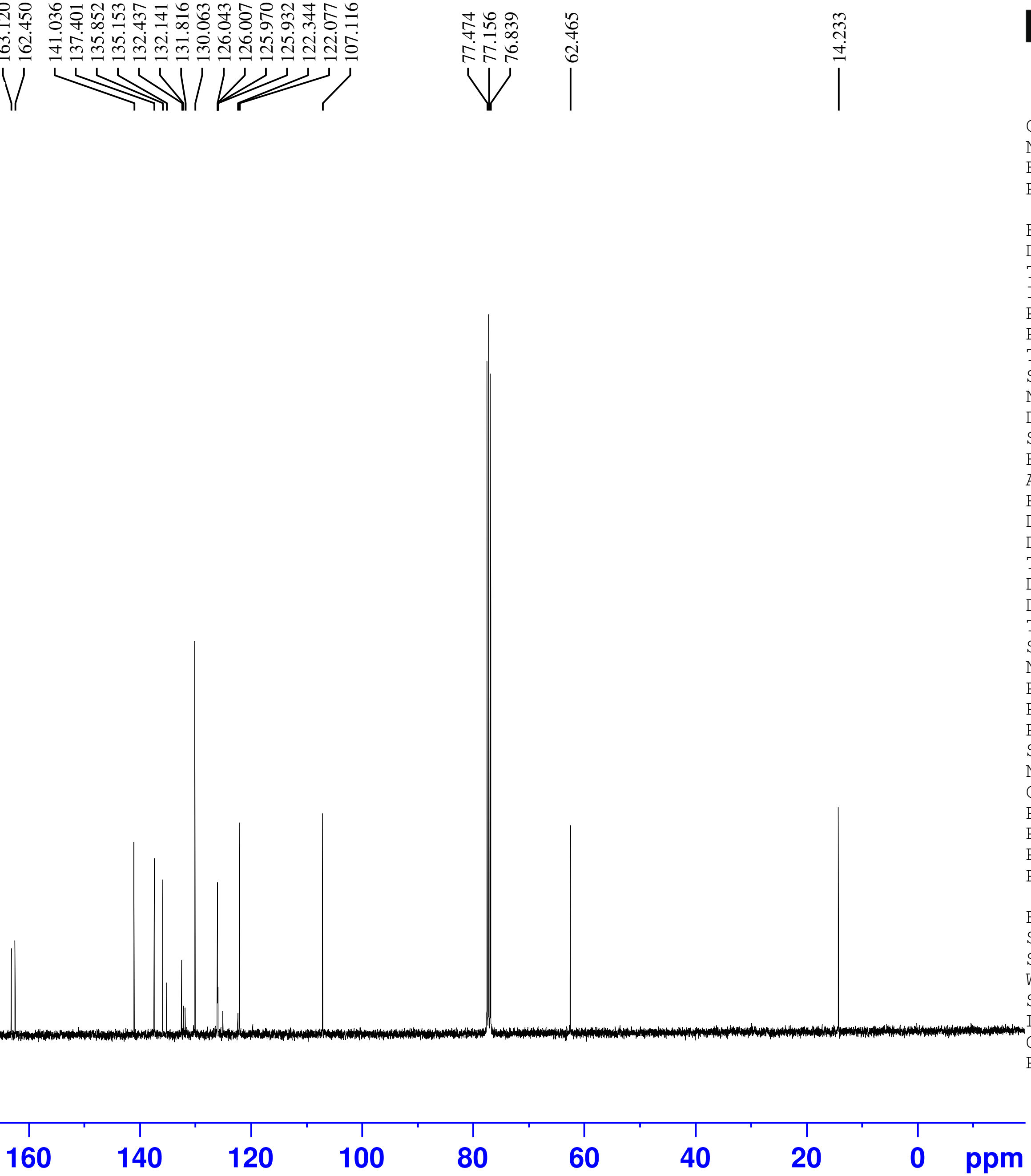

BRUKER

Current Data Parameters
NAME 13C gns-11-092 2
EXPNO 1
PROCNO 1

F2 - Acquisition Parameters
Date_ 20210126
Time 17.27 h
INSTRUM spect
PROBHD Z104450_0192 (
PULPROG zgpg30
TD 65536
SOLVENT CDCl3
NS 500
DS 2
SWH 24038.461 Hz
FIDRES 0.733596 Hz
AQ 1.3631488 sec
RG 203
DW 20.800 usec
DE 6.50 usec
TE 298.2 K
D1 2.00000000 sec
D11 0.03000000 sec
TD0 1
SFO1 100.6228298 MHz
NUC1 13C
P0 3.28 usec
P1 9.85 usec
PLW1 28.63999939 W
SFO2 400.1316005 MHz
NUC2 1H
CPDPRG[2 waltz65
PCPD2 90.00 usec
PLW2 8.47000027 W
PLW12 0.23528001 W
PLW13 0.11834000 W

F2 - Processing parameters
SI 32768
SF 100.6127565 MHz
WDW EM
SSB 0
LB 1.00 Hz
GB 0
PC 1.40

F19CPD

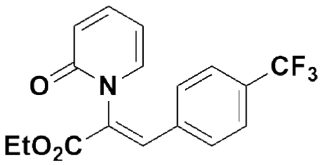

3c

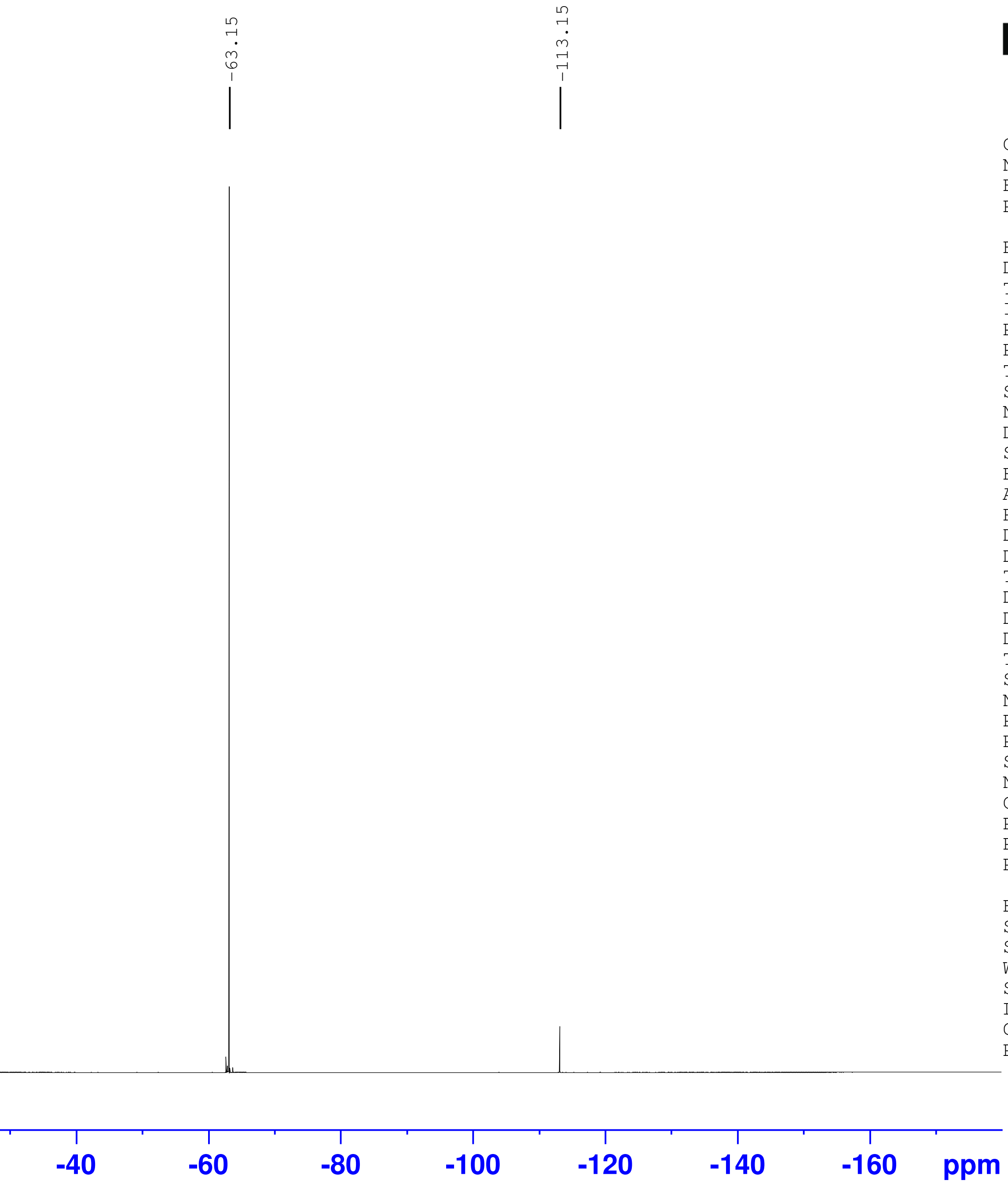

Current Data Parameters
NAME 19F gns-11-092 2
EXPNO 1
PROCNO 1

F2 - Acquisition Parameters
Date_ 20210126
Time 18.05 h
INSTRUM spect
PROBHD Z104450_0192 (
PULPROG zgfhigqn.2
TD 131072
SOLVENT CDCl3
NS 32
DS 4
SWH 89285.711 Hz
FIDRES 1.362392 Hz
AQ 0.7340032 sec
RG 1030
DW 5.600 usec
DE 6.50 usec
TE 298.1 K
D1 1.00000000 sec
D11 0.03000000 sec
D12 0.00002000 sec
TD0 1
SFO1 376.4607164 MHz
NUC1 19F
P1 14.50 usec
PLW1 11.00000000 W
SFO2 400.1316005 MHz
NUC2 1H
CPDPRG[2 waltz16
PCPD2 90.00 usec
PLW2 8.47000027 W
PLW12 0.23528001 W

F2 - Processing parameters
SI 65536
SF 376.4983880 MHz
WDW EM
SSB 0
LB 0.30 Hz
GB 0
PC 1.00

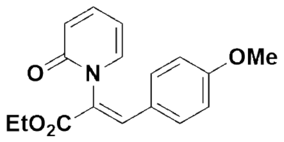

3f

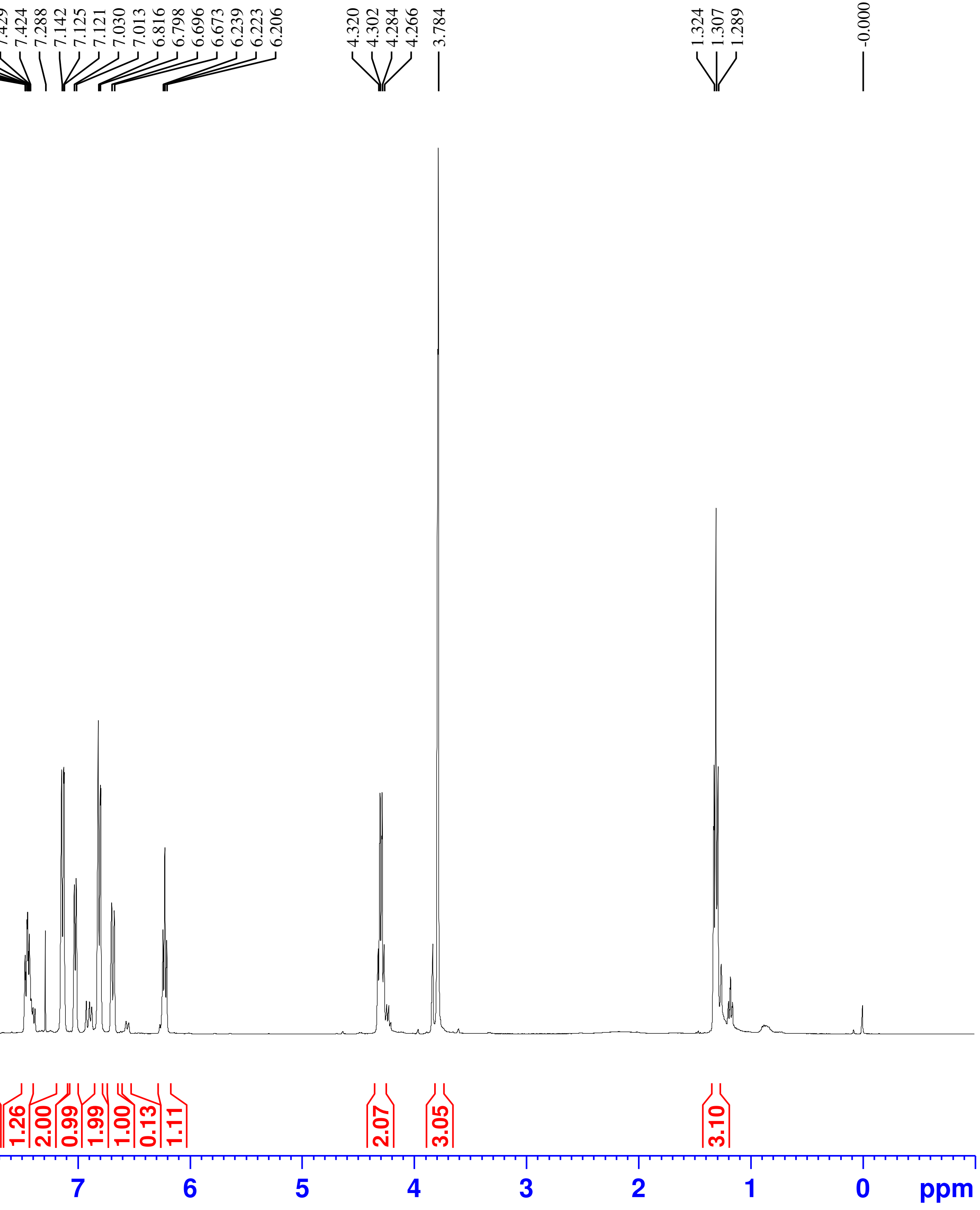


```
Current Data Parameters
NAME      1H gns-12-074 3
EXPNO                    1
PROCNO                   1

F2 - Acquisition Parameters
Date_             20210414
Time                 14.36 h
INSTRUM              spect
PROBHD    Z104450_0192 (
PULPROG               zg30
TD                   65536
SOLVENT              CDCl3
NS                      16
DS                       2
SWH               8012.820 Hz
FIDRES            0.244532 Hz
AQ               4.0894465 sec
RG                    80.6
DW                  62.400 usec
DE                   16.92 usec
TE                   296.6 K
D1              1.00000000 sec
TD0                      1
SFO1           400.1324708 MHz
NUC1                    1H
P0                    5.00 usec
P1                   15.00 usec
PLW1            8.47000027 W

F2 - Processing parameters
SI                   65536
SF             400.1299985 MHz
WDW                     EM
SSB       0
LB                    0.30 Hz
GB        0
PC                    1.00
```

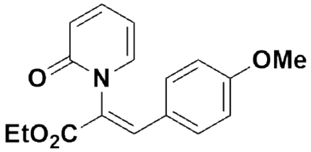

3f

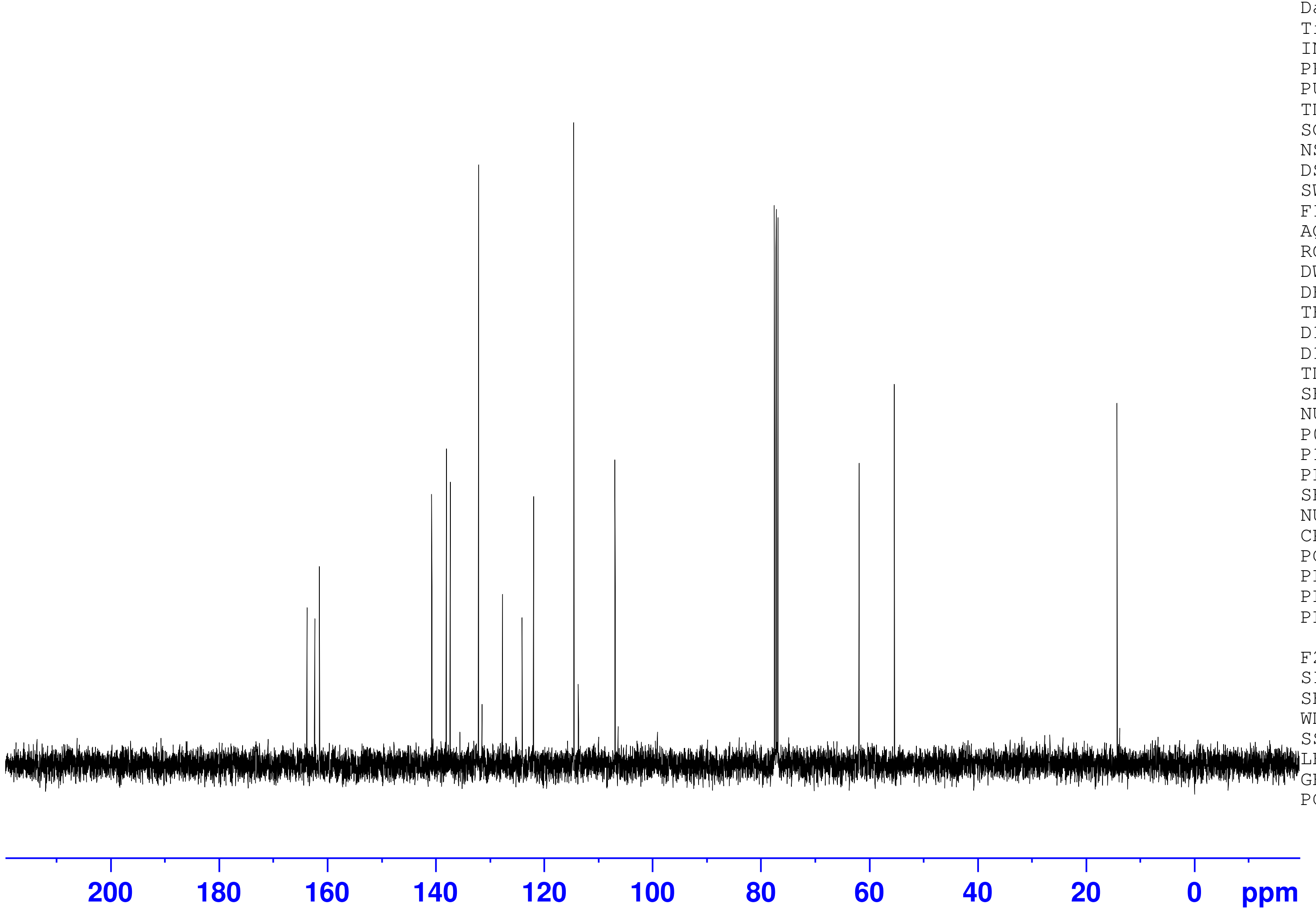

Current Data Parameters

NAME	13C gns-12-074 2
EXPNO	1
PROCNO	1

F2 - Acquisition Parameters

Date_	20210320
Time	19.22 h
INSTRUM	spect
PROBHD	Z104450_0192 (
PULPROG	zgpg30
TD	65536
SOLVENT	CDCl3
NS	11
DS	2
SWH	24038.461 Hz
FIDRES	0.733596 Hz
AQ	1.3631488 sec
RG	203
DW	20.800 usec
DE	6.50 usec
TE	296.5 K
D1	2.00000000 sec
D11	0.03000000 sec
TD0	1
SFO1	100.6228298 MHz
NUC1	13C
P0	3.28 usec
P1	9.85 usec
PLW1	28.63999939 W
SFO2	400.1316005 MHz
NUC2	1H
CPDPRG[2	waltz65
PCPD2	90.00 usec
PLW2	8.47000027 W
PLW12	0.23528001 W
PLW13	0.11834000 W

F2 - Processing parameters

SI	32768
SF	100.6127659 MHz
WDW	EM
SSB	0
LB	1.00 Hz
GB	0
PC	1.40

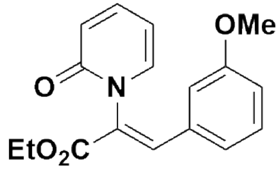

3g

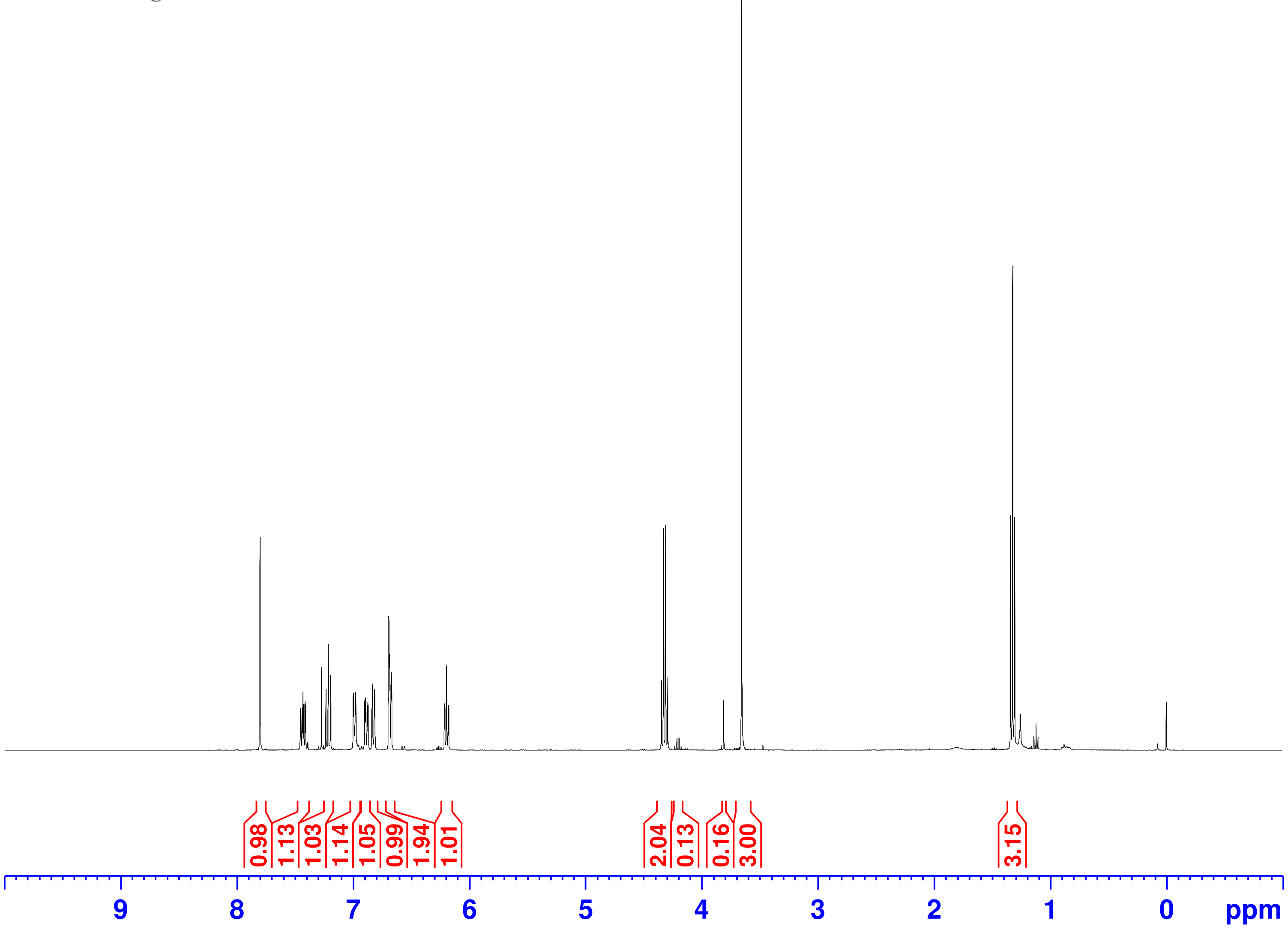

BRUKER

```
Current Data Parameters
NAME       1H gns-11-090
EXPNO                  1
PROCNO                 1

F2 - Acquisition Parameters
Date_           20201221
Time               16.39 h
INSTRUM            spect
PROBHD    Z104450_0192 (
PULPROG             zg30
TD                 65536
SOLVENT            CDCl3
NS                    16
DS                     2
SWH             8012.820 Hz
FIDRES          0.244532 Hz
AQ             4.0894465 sec
RG                   128
DW                62.400 usec
DE                 16.92 usec
TE                 298.0 K
D1            1.00000000 sec
TD0                    1
SFO1         400.1324708 MHz
NUC1                  1H
P0                  5.00 usec
P1                 15.00 usec
PLW1          8.47000027 W

F2 - Processing parameters
SI                 65536
SF           400.1300051 MHz
WDW                   EM
SSB       0
LB                  0.30 Hz
GB        0
PC                  1.00
```

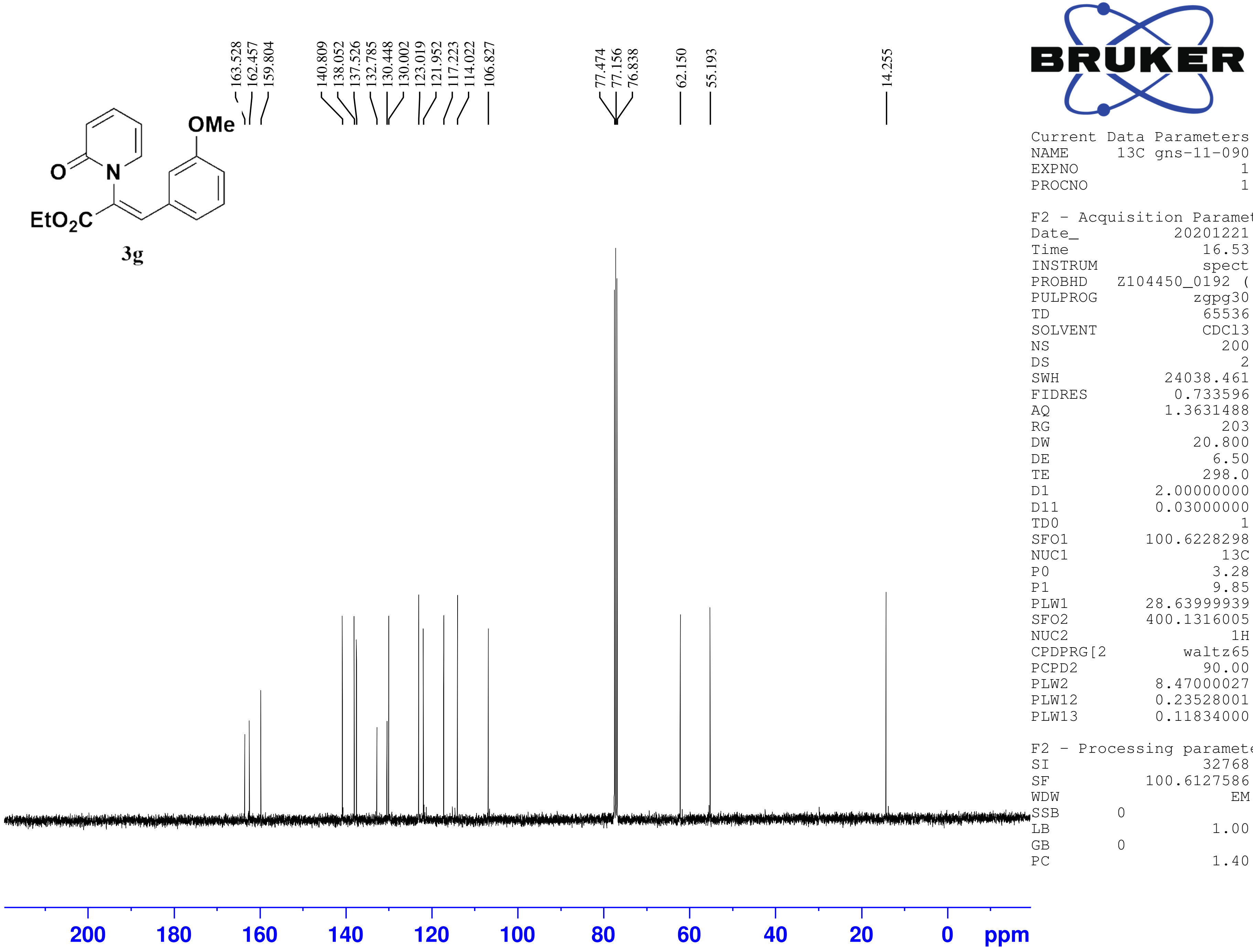

Current Data Parameters
NAME 13C gns-11-090
EXPNO 1
PROCNO 1

F2 - Acquisition Parameters

Date_	20201221
Time	16.53 h
INSTRUM	spect
PROBHD	Z104450_0192 (
PULPROG	zgpg30
TD	65536
SOLVENT	CDCl3
NS	200
DS	2
SWH	24038.461 Hz
FIDRES	0.733596 Hz
AQ	1.3631488 sec
RG	203
DW	20.800 usec
DE	6.50 usec
TE	298.0 K
D1	2.00000000 sec
D11	0.03000000 sec
TD0	1
SFO1	100.6228298 MHz
NUC1	13C
P0	3.28 usec
P1	9.85 usec
PLW1	28.63999939 W
SFO2	400.1316005 MHz
NUC2	1H
CPDPRG[2	waltz65
PCPD2	90.00 usec
PLW2	8.47000027 W
PLW12	0.23528001 W
PLW13	0.11834000 W

F2 - Processing parameters

SI	32768
SF	100.6127586 MHz
WDW	EM
SSB	0
LB	1.00 Hz
GB	0
PC	1.40

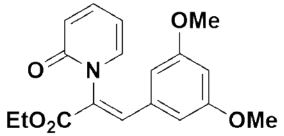

3h

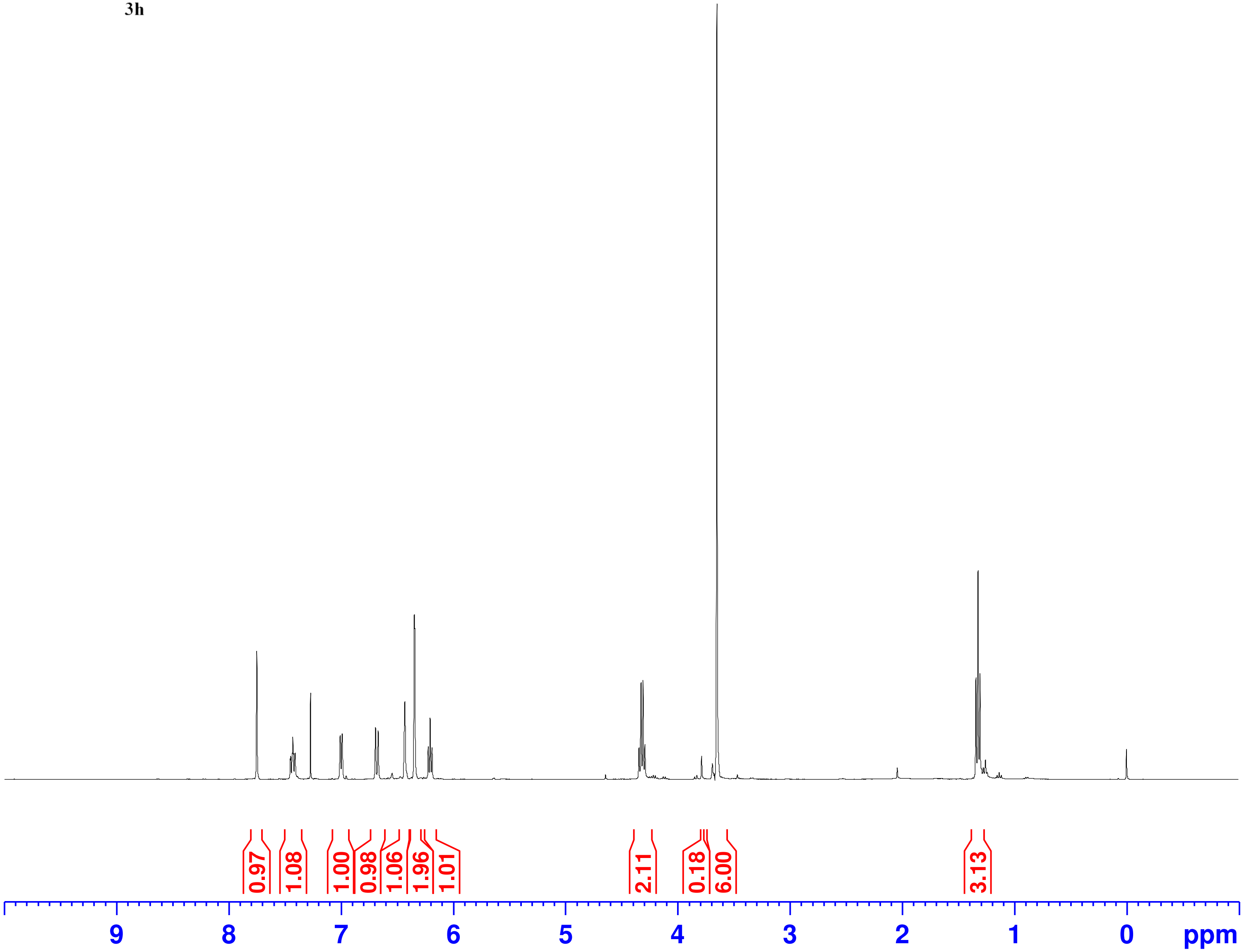


```
Current Data Parameters
NAME        1H gns-11-110
EXPNO                   1
PROCNO                  1

F2 - Acquisition Parameters
Date_            20210110
Time                12.52 h
INSTRUM             spect
PROBHD    Z104450_0192 (
PULPROG              zg30
TD                  65536
SOLVENT             CDCl3
NS                     16
DS                      2
SWH              8012.820 Hz
FIDRES           0.244532 Hz
AQ              4.0894465 sec
RG                    144
DW                 62.400 usec
DE                  16.92 usec
TE                  297.2 K
D1             1.00000000 sec
TD0                     1
SFO1          400.1324708 MHz
NUC1                   1H
P0                   5.00 usec
P1                  15.00 usec
PLW1           8.47000027 W

F2 - Processing parameters
SI                  65536
SF            400.1300052 MHz
WDW                    EM
SSB      0
LB                   0.30 Hz
GB       0
PC                   1.00
```

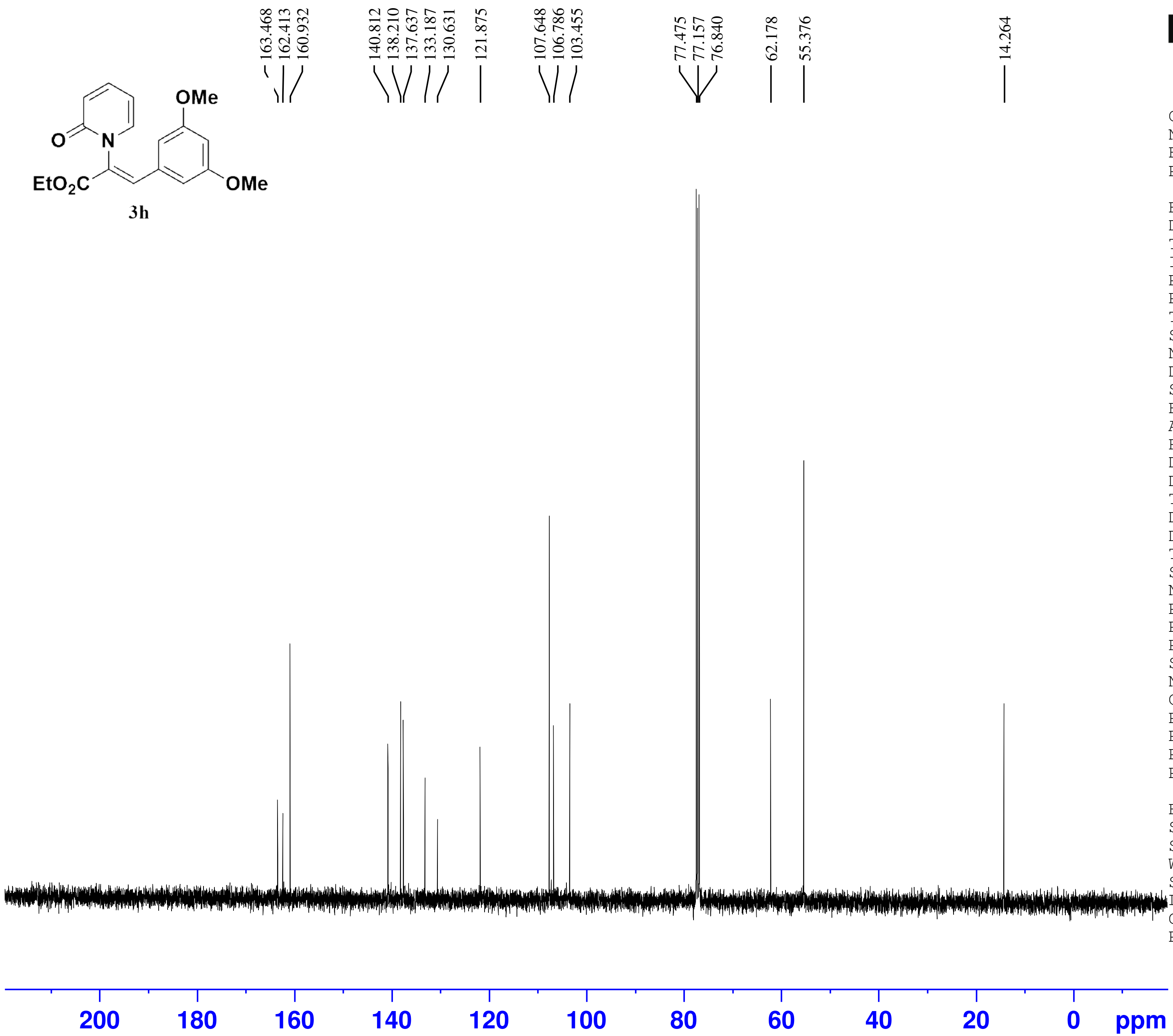


```
Current Data Parameters
NAME        13C gns-11-110
EXPNO                    1
PROCNO                   1

F2 - Acquisition Parameters
Date_             20210110
Time                 13.01 h
INSTRUM              spect
PROBHD   Z104450_0192 (
PULPROG            zgpg30
TD                   65536
SOLVENT              CDCl3
NS                     100
DS                       2
SWH              24038.461 Hz
FIDRES            0.733596 Hz
AQ               1.3631488 sec
RG                     203
DW                  20.800 usec
DE                    6.50 usec
TE                   297.8 K
D1              2.00000000 sec
D11             0.03000000 sec
TD0                      1
SFO1           100.6228298 MHz
NUC1                   13C
P0                    3.28 usec
P1                    9.85 usec
PLW1           28.63999939 W
SFO2           400.1316005 MHz
NUC2                    1H
CPDPRG[2           waltz65
PCPD2                90.00 usec
PLW2            8.47000027 W
PLW12           0.23528001 W
PLW13           0.11834000 W

F2 - Processing parameters
SI                   32768
SF             100.6127581 MHz
WDW                     EM
SSB        0
LB                    1.00 Hz
GB         0
PC                    1.40
```

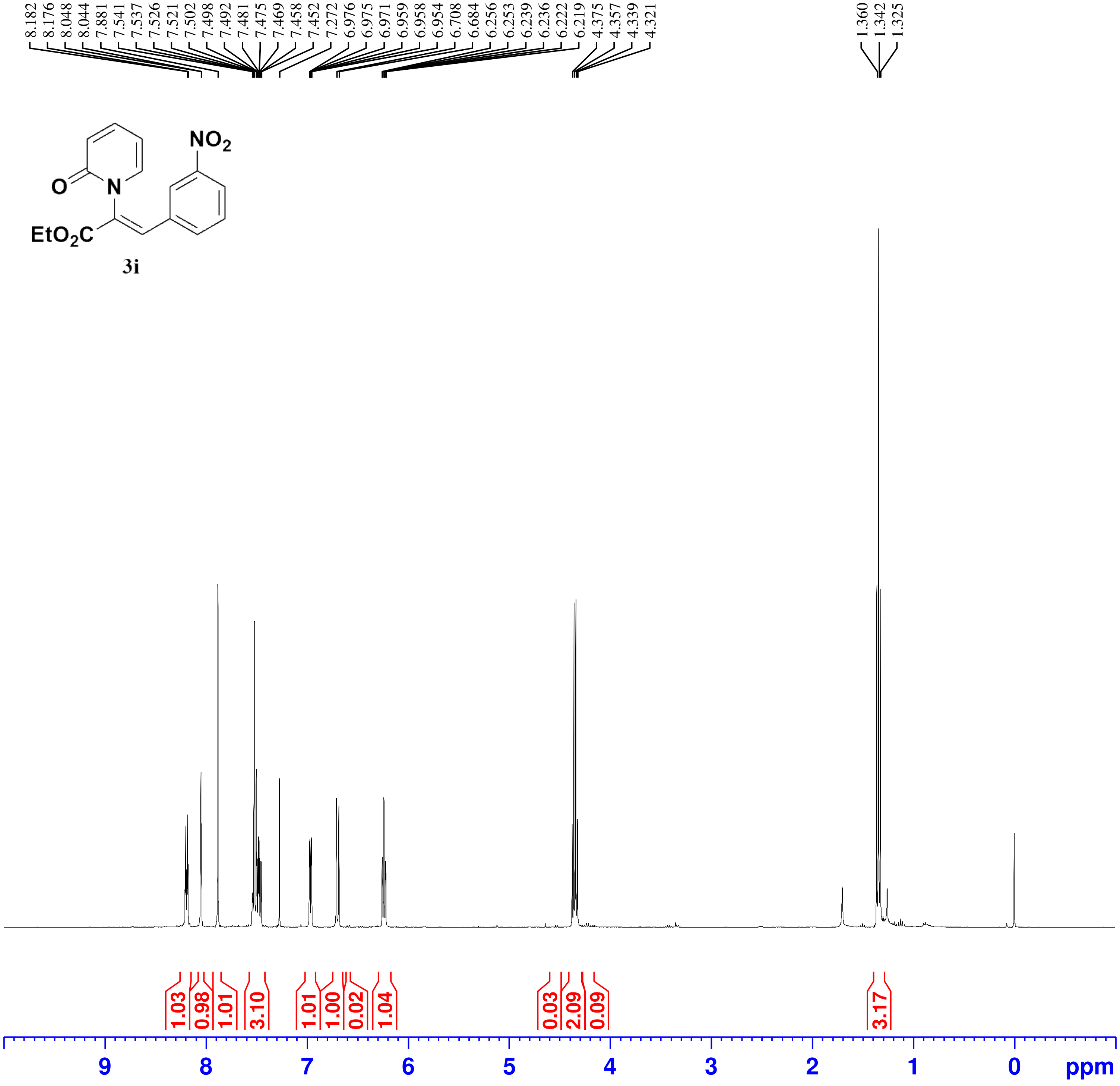

BRUKER

Current Data Parameters
NAME 1H gns-11-128
EXPNO 1
PROCNO 1

F2 - Acquisition Parameters
Date_ 20210120
Time 17.40 h
INSTRUM spect
PROBHD Z104450_0192 (
PULPROG zg30
TD 65536
SOLVENT CDCl3
NS 16
DS 2
SWH 8012.820 Hz
FIDRES 0.244532 Hz
AQ 4.0894465 sec
RG 203
DW 62.400 usec
DE 16.92 usec
TE 298.0 K
D1 1.00000000 sec
TD0 1
SFO1 400.1324708 MHz
NUC1 1H
P0 5.00 usec
P1 15.00 usec
PLW1 8.47000027 W

F2 - Processing parameters
SI 65536
SF 400.1300050 MHz
WDW EM
SSB 0
LB 0.30 Hz
GB 0
PC 1.00

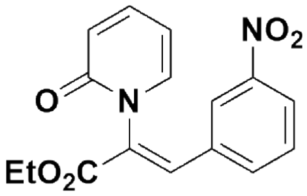

3i

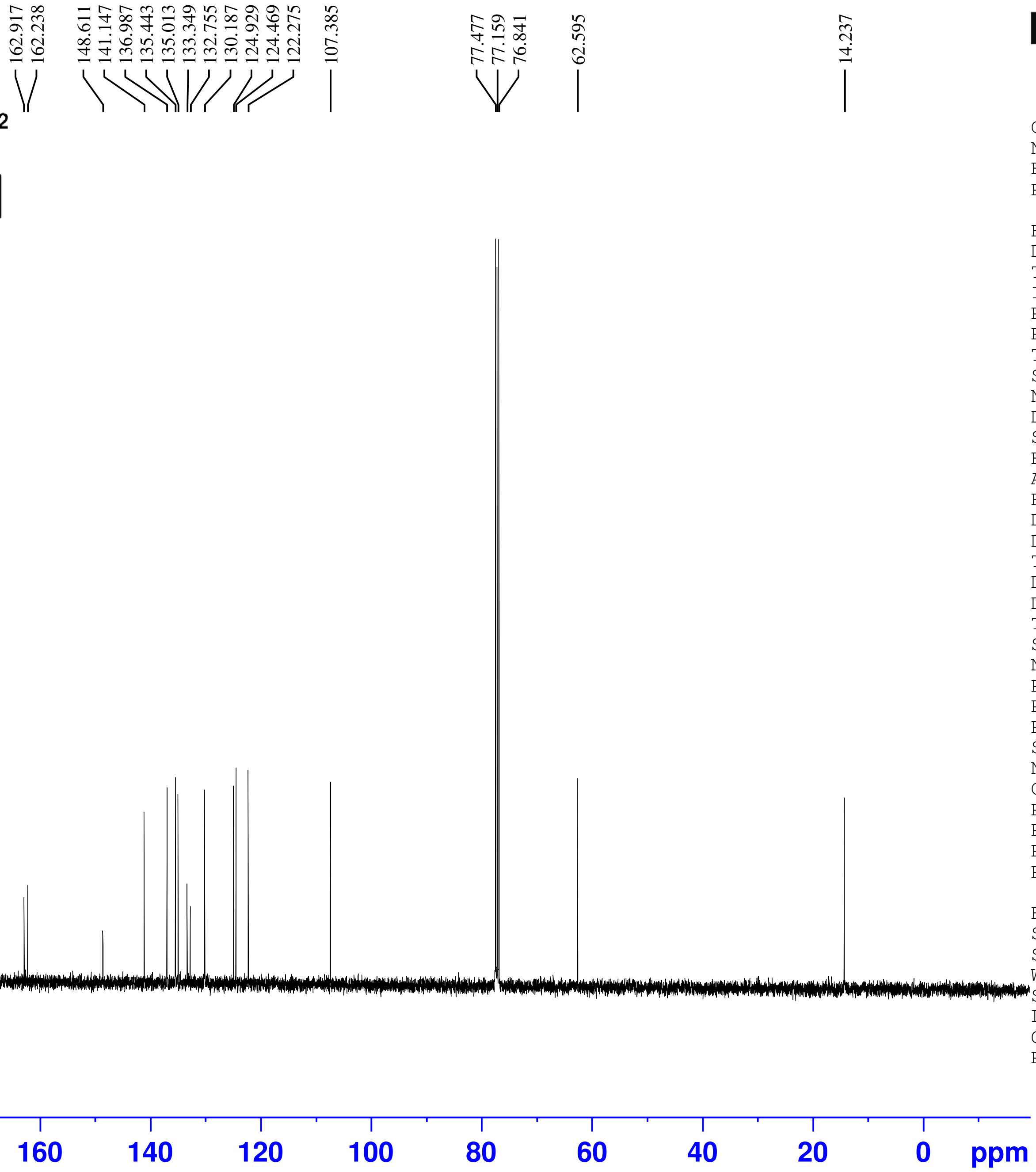

BRUKER

```
Current Data Parameters
NAME       13C gns-11-128
EXPNO                   1
PROCNO                  1

F2 - Acquisition Parameters
Date_            20210120
Time                17.54 h
INSTRUM             spect
PROBHD     Z104450_0192 (
PULPROG           zgpg30
TD                  65536
SOLVENT             CDCl3
NS                    200
DS                      2
SWH             24038.461 Hz
FIDRES           0.733596 Hz
AQ              1.3631488 sec
RG                    203
DW                 20.800 usec
DE                   6.50 usec
TE                  298.0 K
D1             2.00000000 sec
D11            0.03000000 sec
TD0                     1
SFO1          100.6228298 MHz
NUC1                  13C
P0                   3.28 usec
P1                   9.85 usec
PLW1          28.63999939 W
SFO2          400.1316005 MHz
NUC2                   1H
CPDPRG[2          waltz65
PCPD2               90.00 usec
PLW2           8.47000027 W
PLW12          0.23528001 W
PLW13          0.11834000 W

F2 - Processing parameters
SI                  32768
SF            100.6127572 MHz
WDW                    EM
SSB        0
LB                   1.00 Hz
GB         0
PC                   1.40
```

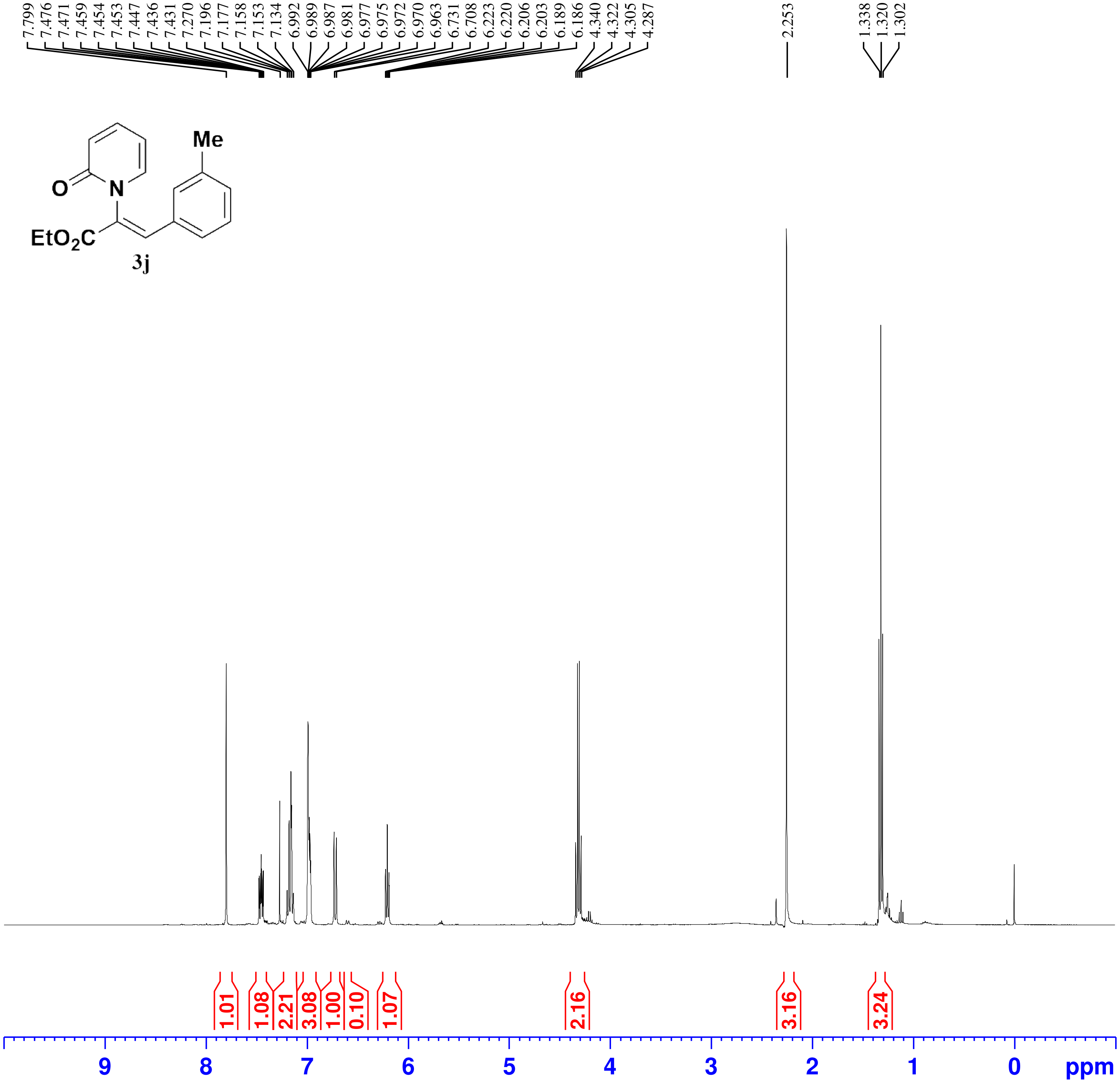

BRUKER

```
Current Data Parameters
NAME        1H gns-11-122
EXPNO                   1
PROCNO                  1

F2 - Acquisition Parameters
Date_            20210114
Time                15.34 h
INSTRUM             spect
PROBHD    Z104450_0192 (
PULPROG              zg30
TD                  65536
SOLVENT             CDCl3
NS                     16
DS                      2
SWH              8012.820 Hz
FIDRES           0.244532 Hz
AQ              4.0894465 sec
RG                    161
DW                 62.400 usec
DE                  16.92 usec
TE                  298.0 K
D1             1.00000000 sec
TD0                     1
SFO1          400.1324708 MHz
NUC1                   1H
P0                   5.00 usec
P1                  15.00 usec
PLW1           8.47000027 W

F2 - Processing parameters
SI                  65536
SF            400.1300058 MHz
WDW                    EM
SSB      0
LB                   0.30 Hz
GB       0
PC                   1.00
```

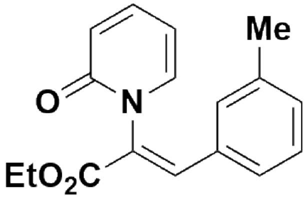

3j

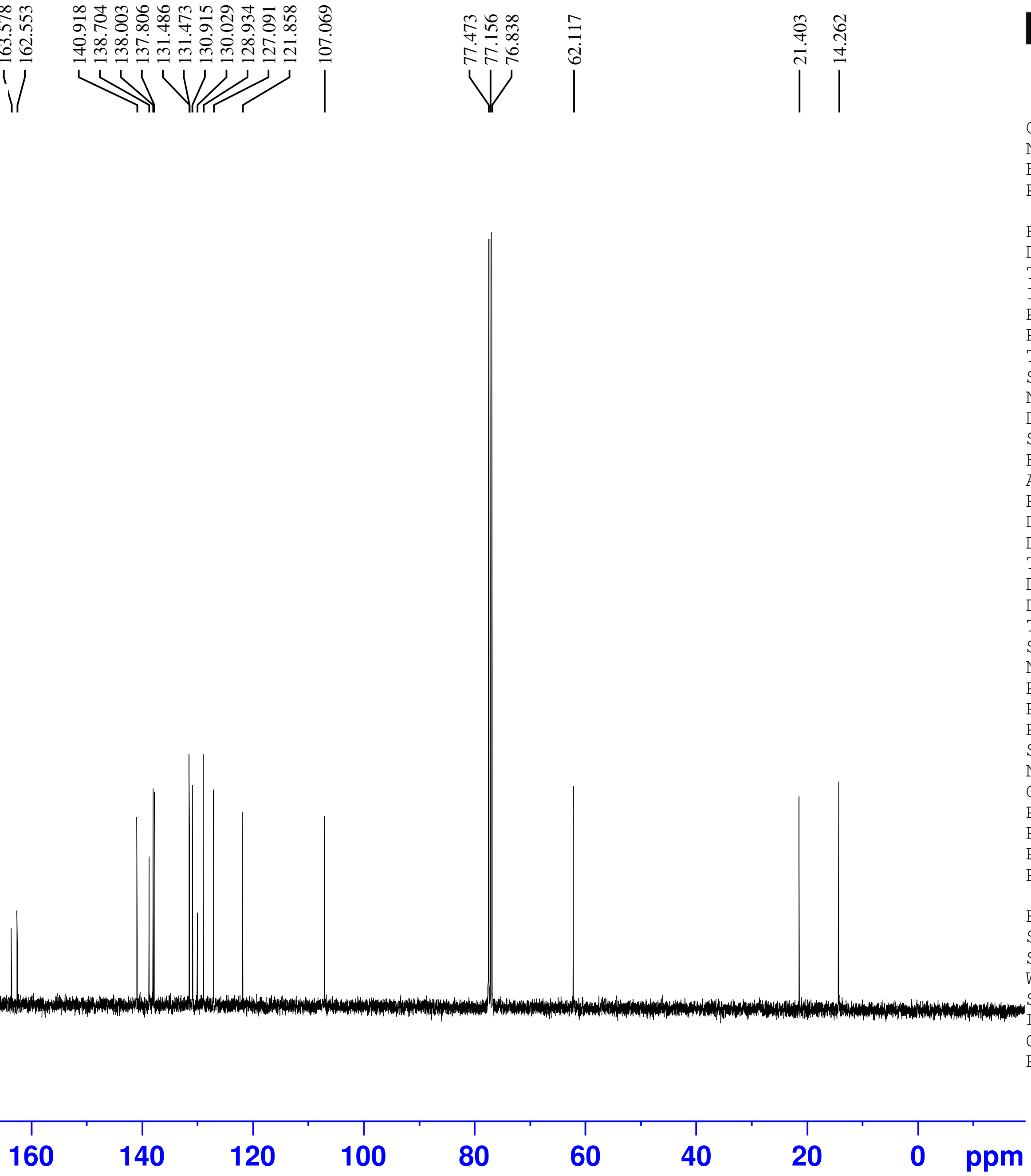

BRUKER

```
Current Data Parameters
NAME        13C gns-11-122
EXPNO                    1
PROCNO                   1

F2 - Acquisition Parameters
Date_             20210114
Time                 15.48 h
INSTRUM              spect
PROBHD    Z104450_0192 (
PULPROG            zgpg30
TD                   65536
SOLVENT              CDCl3
NS                     200
DS                       2
SWH              24038.461 Hz
FIDRES            0.733596 Hz
AQ               1.3631488 sec
RG                     287
DW                  20.800 usec
DE                    6.50 usec
TE                   298.3 K
D1              2.00000000 sec
D11             0.03000000 sec
TD0                      1
SFO1           100.6228298 MHz
NUC1                   13C
P0                    3.28 usec
P1                    9.85 usec
PLW1           28.63999939 W
SFO2           400.1316005 MHz
NUC2                    1H
CPDPRG[2           waltz65
PCPD2                90.00 usec
PLW2            8.47000027 W
PLW12           0.23528001 W
PLW13           0.11834000 W

F2 - Processing parameters
SI                   32768
SF             100.6127584 MHz
WDW                     EM
SSB          0
LB                    1.00 Hz
GB           0
PC                    1.40
```

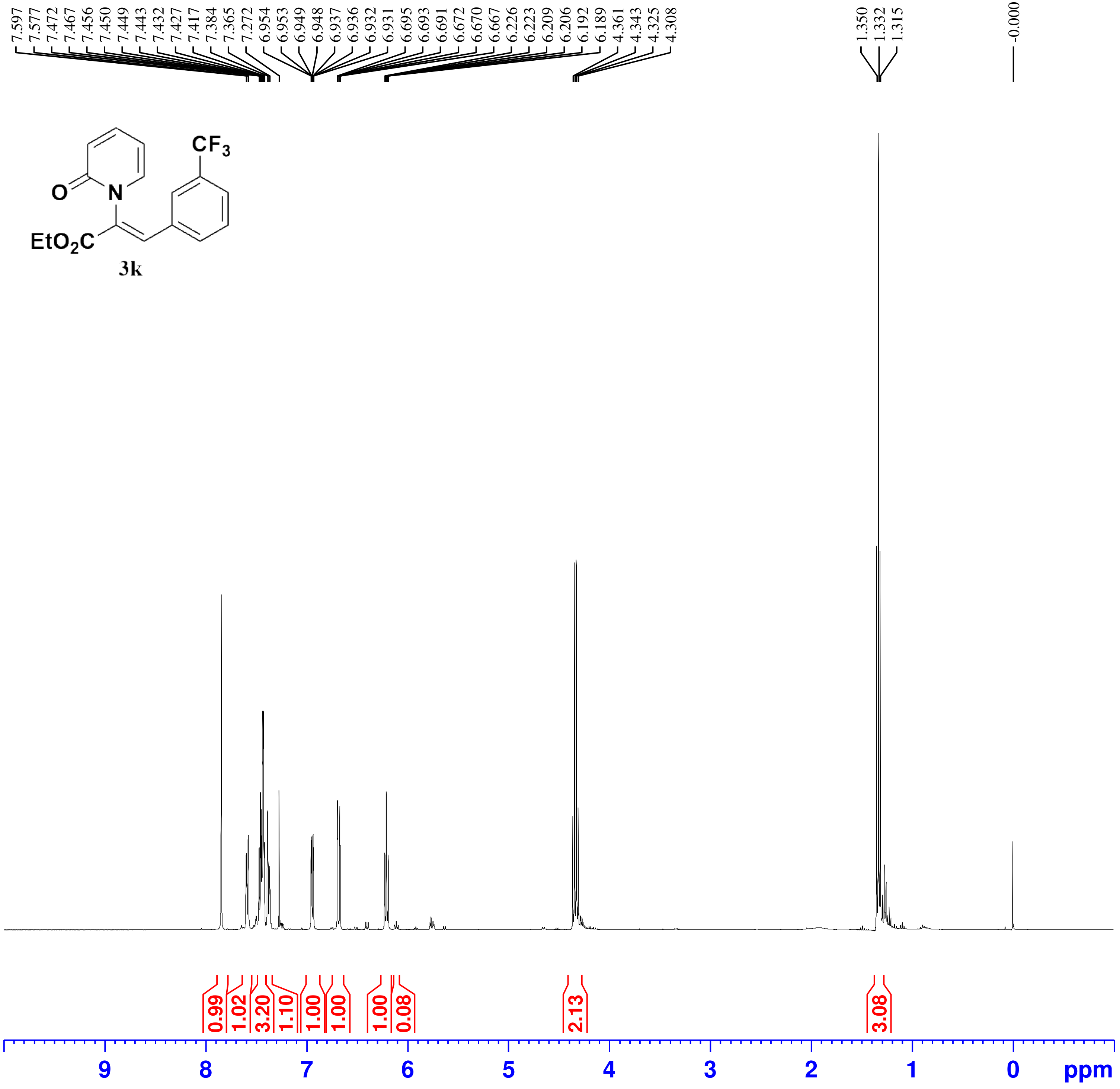

3k

Current Data Parameters

NAME	1H gns-11-118
EXPNO	1
PROCNO	1

F2 - Acquisition Parameters

Date_	20210112
Time	18.07 h
INSTRUM	spect
PROBHD	Z104450_0192 (
PULPROG	zg30
TD	65536
SOLVENT	CDCl3
NS	16
DS	2
SWH	8012.820 Hz
FIDRES	0.244532 Hz
AQ	4.0894465 sec
RG	161
DW	62.400 usec
DE	16.92 usec
TE	298.0 K
D1	1.00000000 sec
TD0	1
SFO1	400.1324708 MHz
NUC1	1H
P0	5.00 usec
P1	15.00 usec
PLW1	8.47000027 W

F2 - Processing parameters

SI	65536
SF	400.1300046 MHz
WDW	EM
SSB	0
LB	0.30 Hz
GB	0
PC	1.00

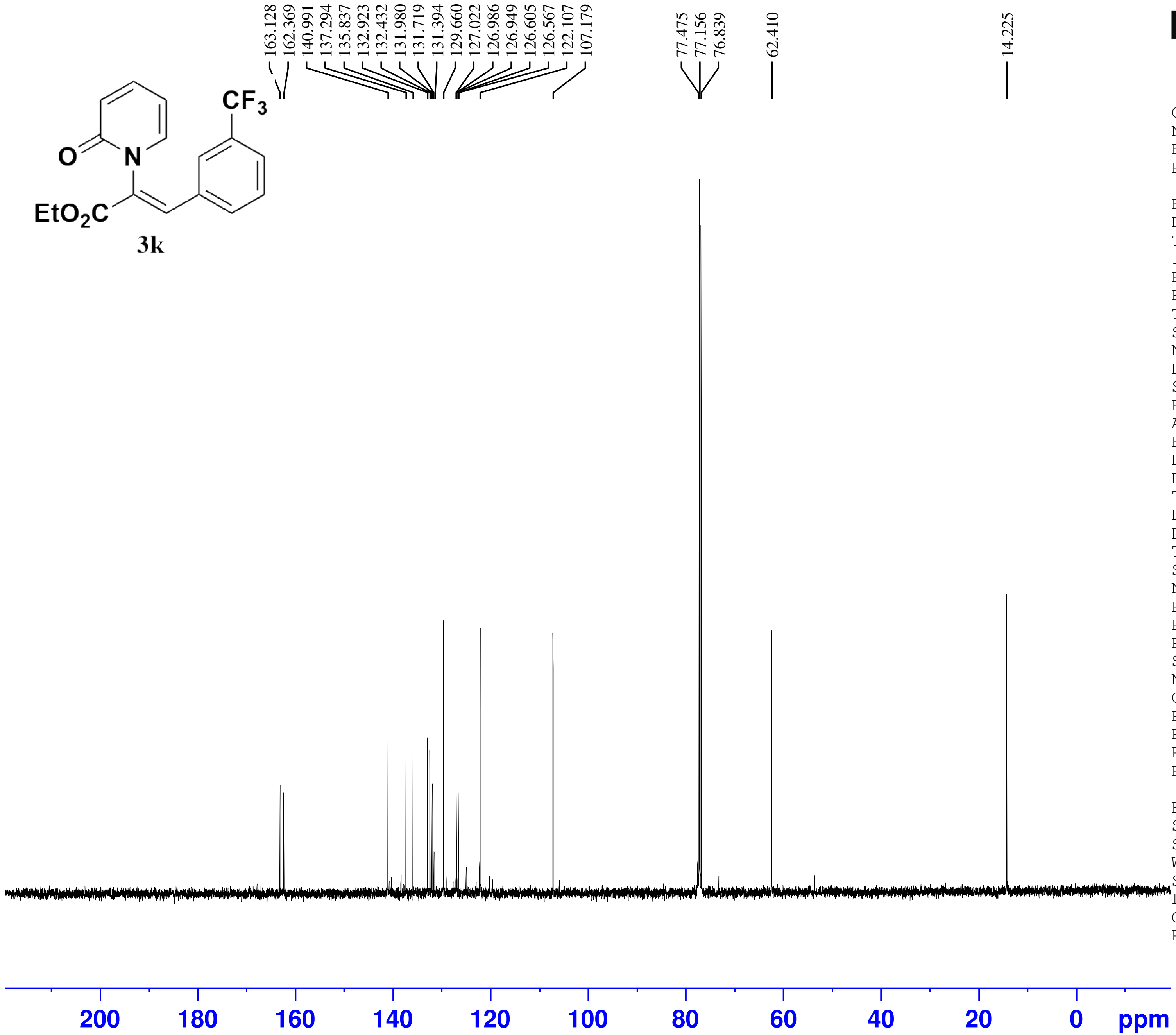

BRUKER

Current Data Parameters
NAME 13C gns-11-118 2
EXPNO 1
PROCNO 1

F2 - Acquisition Parameters
Date_ 20210126
Time 17.59 h
INSTRUM spect
PROBHD Z104450_0192 (
PULPROG zgpg30
TD 65536
SOLVENT CDCl3
NS 500
DS 2
SWH 24038.461 Hz
FIDRES 0.733596 Hz
AQ 1.3631488 sec
RG 203
DW 20.800 usec
DE 6.50 usec
TE 298.3 K
D1 2.00000000 sec
D11 0.03000000 sec
TD0 1
SFO1 100.6228298 MHz
NUC1 13C
P0 3.28 usec
P1 9.85 usec
PLW1 28.63999939 W
SFO2 400.1316005 MHz
NUC2 1H
CPDPRG[2 waltz65
PCPD2 90.00 usec
PLW2 8.47000027 W
PLW12 0.23528001 W
PLW13 0.11834000 W

F2 - Processing parameters
SI 32768
SF 100.6127580 MHz
WDW EM
SSB 0
LB 1.00 Hz
GB 0
PC 1.40

F19CPD

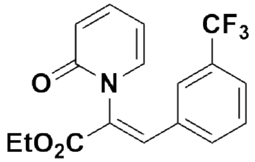

3k

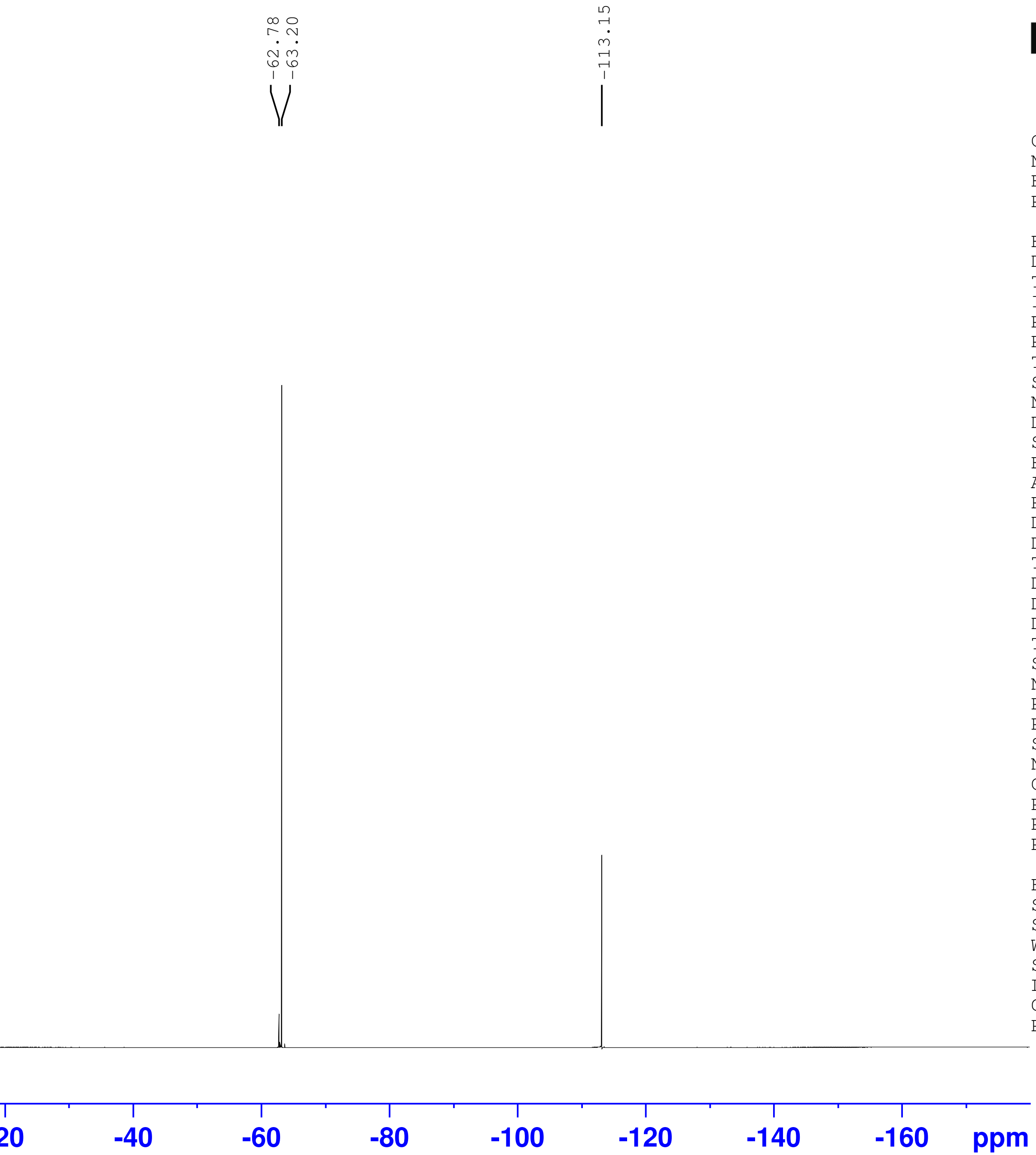


```
Current Data Parameters
NAME      19F gns-11-118 2
EXPNO                    1
PROCNO                   1

F2 - Acquisition Parameters
Date_             20210126
Time                 18.13 h
INSTRUM              spect
PROBHD    Z104450_0192 (
PULPROG         zgfhigqn.2
TD                  131072
SOLVENT              CDCl3
NS                      32
DS                       4
SWH              89285.711 Hz
FIDRES            1.362392 Hz
AQ               0.7340032 sec
RG                    1290
DW                   5.600 usec
DE                    6.50 usec
TE                   298.1 K
D1              1.00000000 sec
D11             0.03000000 sec
D12             0.00002000 sec
TD0                      1
SFO1           376.4607164 MHz
NUC1                   19F
P1                   14.50 usec
PLW1           11.00000000 W
SFO2           400.1316005 MHz
NUC2                    1H
CPDPRG[2           waltz16
PCPD2                90.00 usec
PLW2            8.47000027 W
PLW12           0.23528001 W

F2 - Processing parameters
SI                   65536
SF             376.4983877 MHz
WDW                     EM
SSB      0
LB                    0.30 Hz
GB       0
PC                    1.00
```

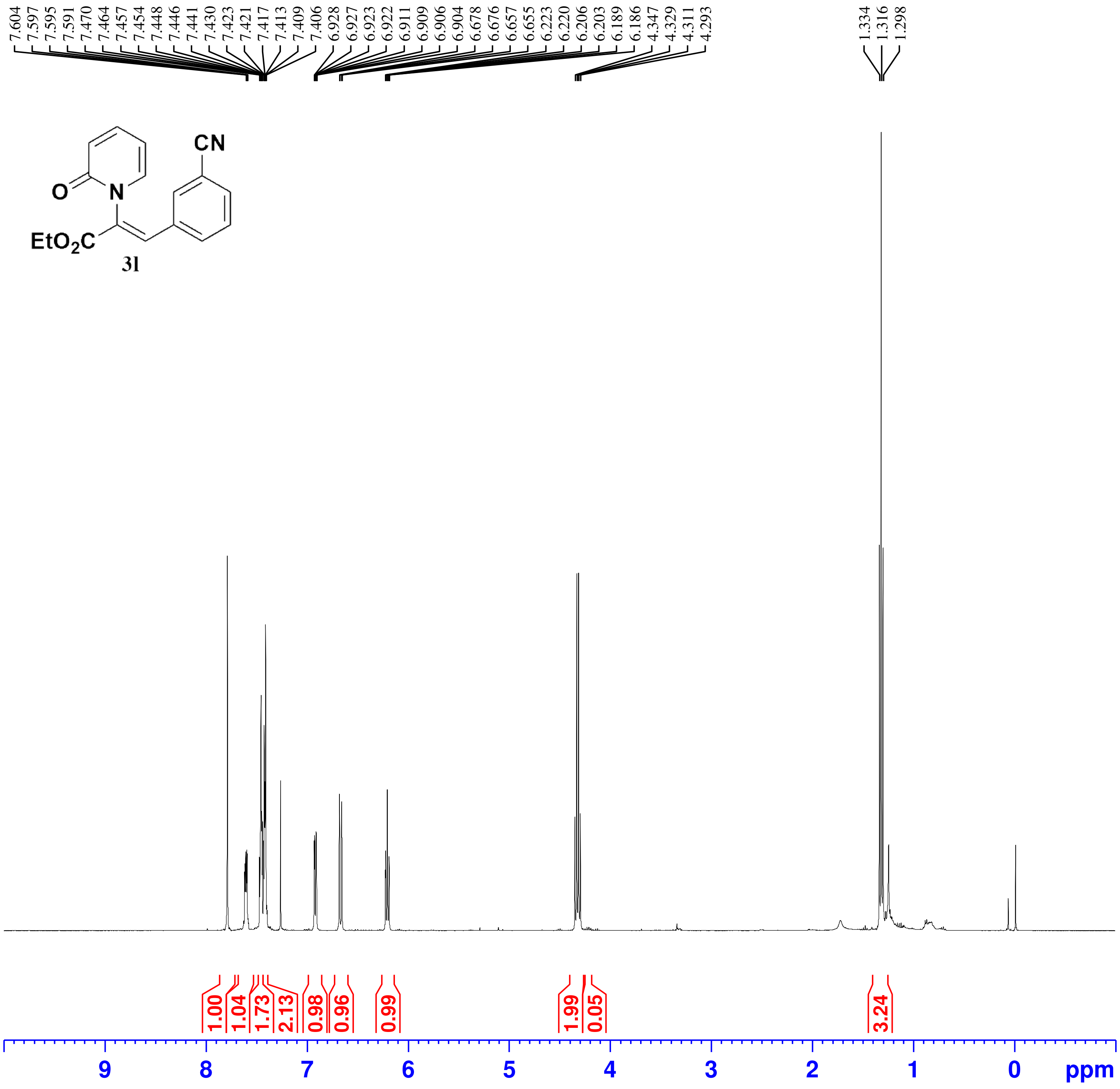

3l

```
Current Data Parameters
NAME       1H gns-11-132 3
EXPNO                    1
PROCNO                   1

F2 - Acquisition Parameters
Date_             20210123
Time                 16.25 h
INSTRUM              spect
PROBHD    Z104450_0192 (
PULPROG               zg30
TD                   65536
SOLVENT              CDCl3
NS                      16
DS                       2
SWH              8012.820 Hz
FIDRES           0.244532 Hz
AQ              4.0894465 sec
RG                     161
DW                  62.400 usec
DE                   16.92 usec
TE                   298.0 K
D1              1.00000000 sec
TD0                      1
SFO1           400.1324708 MHz
NUC1                    1H
P0                    5.00 usec
P1                   15.00 usec
PLW1            8.47000027 W

F2 - Processing parameters
SI                   65536
SF             400.1300096 MHz
WDW                     EM
SSB      0
LB                    0.30 Hz
GB       0
PC                    1.00
```

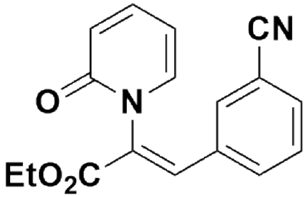

3l

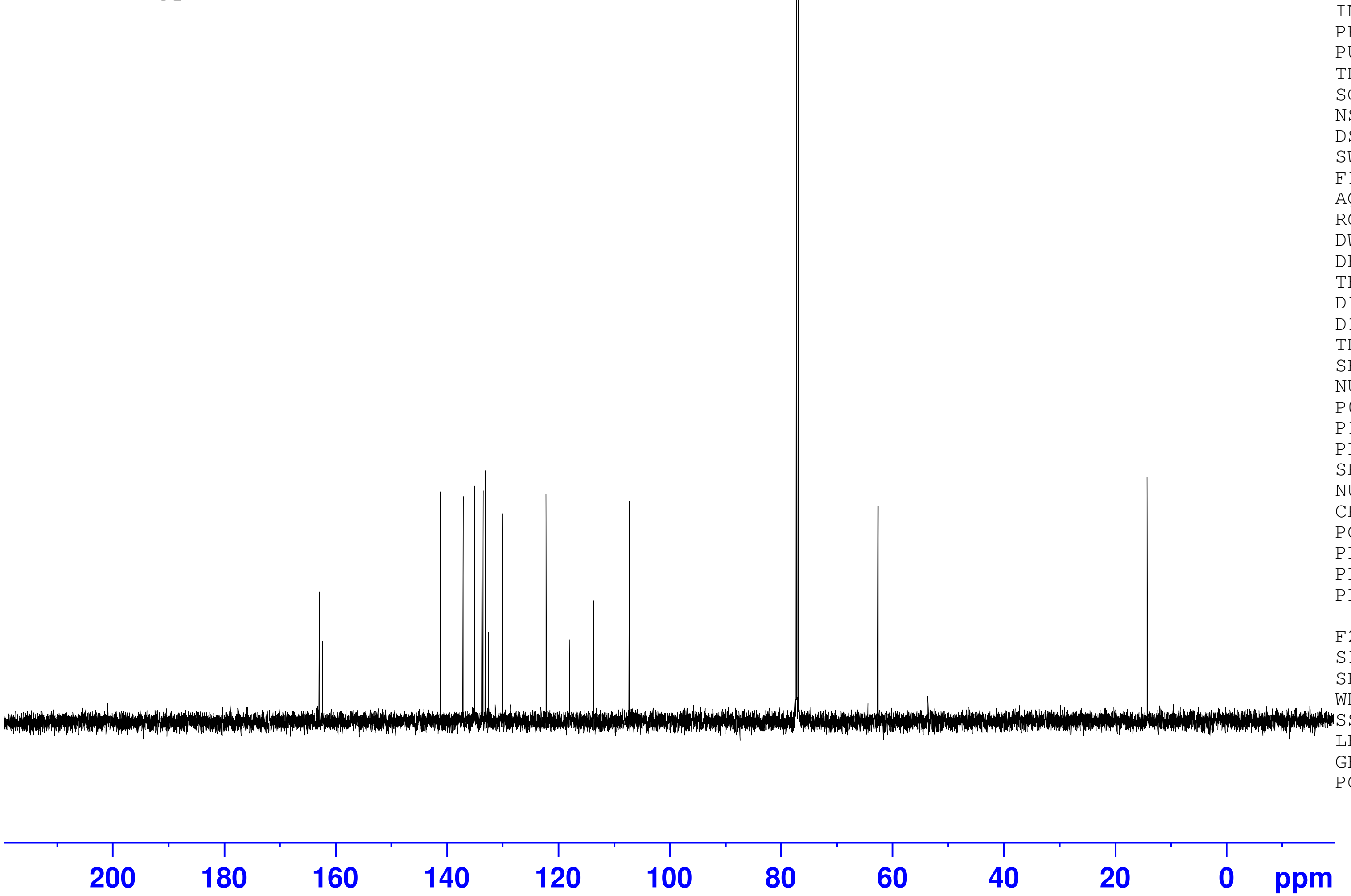

Current Data Parameters
NAME 13C gns-11-132 2
EXPNO 10
PROCNO 1

F2 - Acquisition Parameters
Date_ 20210122
Time 11.15 h
INSTRUM spect
PROBHD Z104450_0192 (
PULPROG zgpg30
TD 65536
SOLVENT CDCl3
NS 100
DS 2
SWH 24038.461 Hz
FIDRES 0.733596 Hz
AQ 1.3631488 sec
RG 203
DW 20.800 usec
DE 6.50 usec
TE 298.2 K
D1 2.00000000 sec
D11 0.03000000 sec
TD0 1
SFO1 100.6228298 MHz
NUC1 13C
P0 3.28 usec
P1 9.85 usec
PLW1 28.63999939 W
SFO2 400.1316005 MHz
NUC2 1H
CPDPRG[2 waltz65
PCPD2 90.00 usec
PLW2 8.47000027 W
PLW12 0.23528001 W
PLW13 0.11834000 W

F2 - Processing parameters
SI 32768
SF 100.6127586 MHz
WDW EM
SSB 0
LB 1.00 Hz
GB 0
PC 1.40

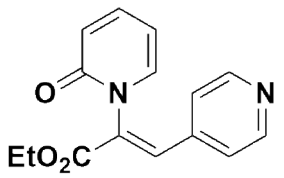

3m

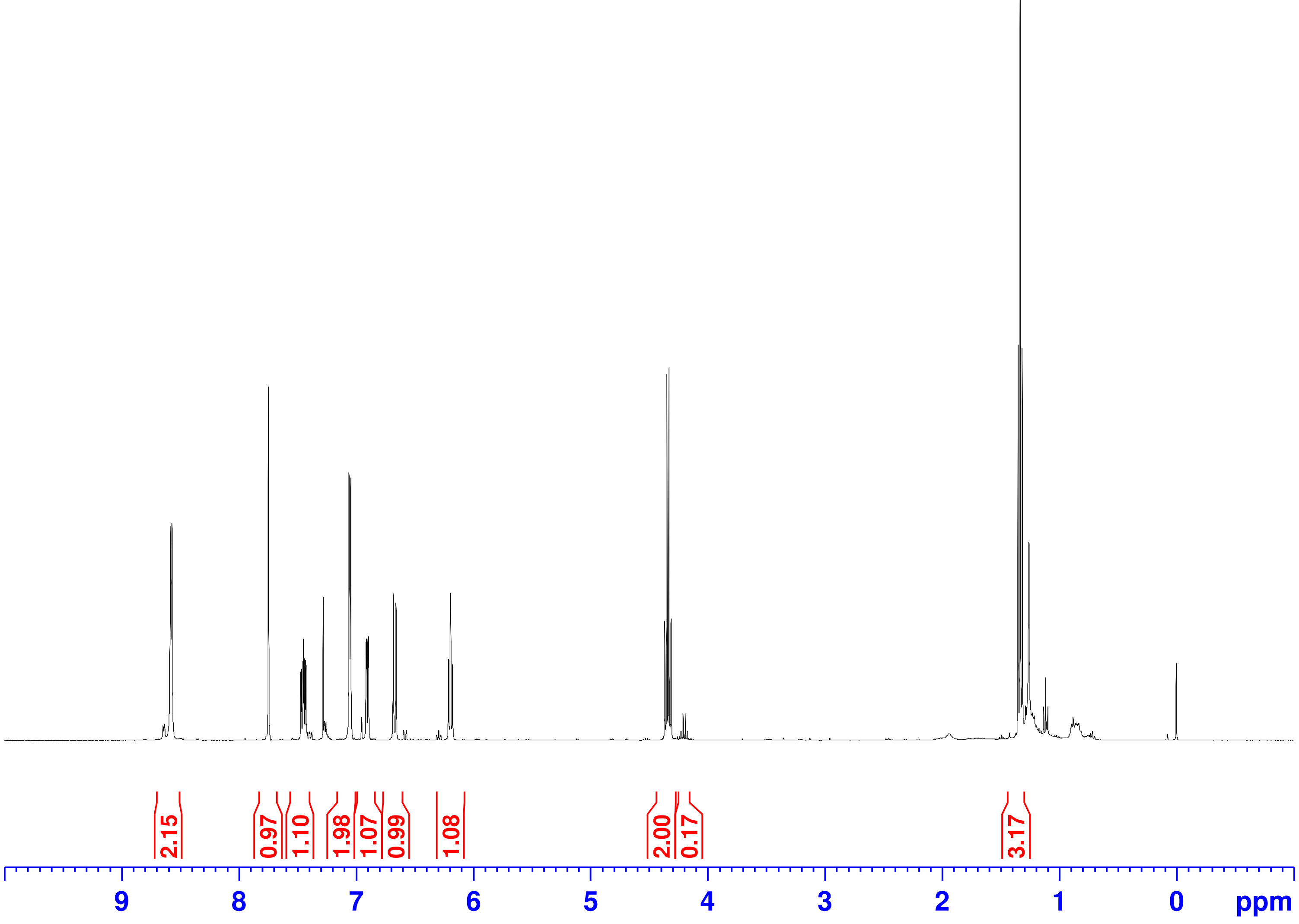

Current Data Parameters
NAME 1H gns-11-066 3
EXPNO 1
PROCNO 1

F2 - Acquisition Parameters
Date_ 20210123
Time 19.28 h
INSTRUM spect
PROBHD Z104450_0192 (
PULPROG zg30
TD 65536
SOLVENT CDCl3
NS 16
DS 2
SWH 8012.820 Hz
FIDRES 0.244532 Hz
AQ 4.0894465 sec
RG 144
DW 62.400 usec
DE 16.92 usec
TE 298.0 K
D1 1.00000000 sec
TD0 1
SFO1 400.1324708 MHz
NUC1 1H
P0 5.00 usec
P1 15.00 usec
PLW1 8.47000027 W

F2 - Processing parameters
SI 65536
SF 400.1300016 MHz
WDW EM
SSB 0
LB 0.30 Hz
GB 0
PC 1.00

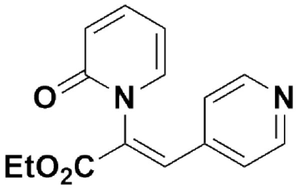

3m

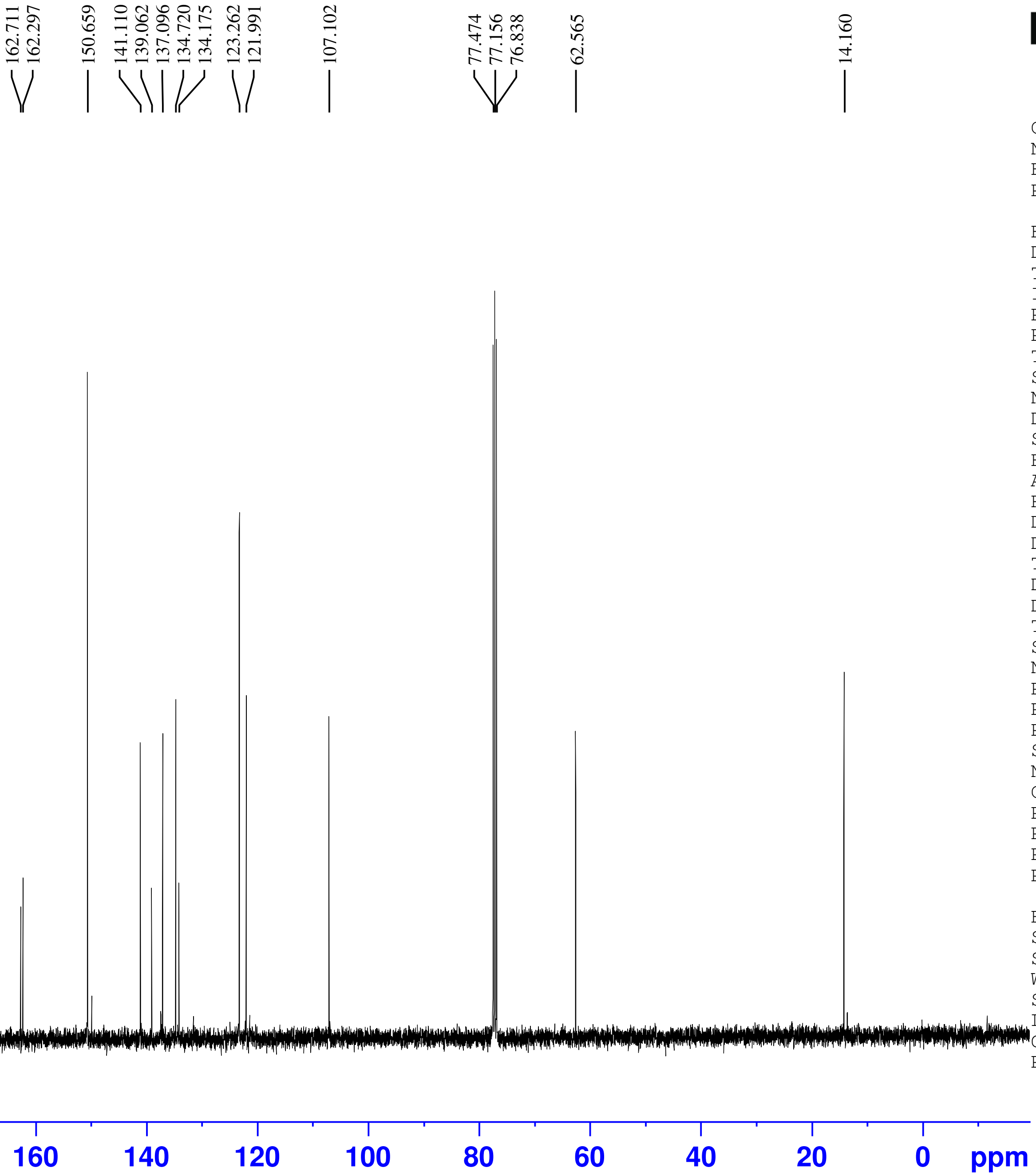

BRUKER

```
Current Data Parameters
NAME       13C gns-11-066
EXPNO                   1
PROCNO                  1

F2 - Acquisition Parameters
Date_            20201204
Time                12.33 h
INSTRUM             spect
PROBHD    Z104450_0192 (
PULPROG           zgpg30
TD                 65536
SOLVENT            CDCl3
NS                   100
DS                     2
SWH            24038.461 Hz
FIDRES          0.733596 Hz
AQ             1.3631488 sec
RG                   203
DW                20.800 usec
DE                  6.50 usec
TE                 297.8 K
D1            2.00000000 sec
D11           0.03000000 sec
TD0                    1
SFO1         100.6228298 MHz
NUC1                 13C
P0                  3.28 usec
P1                  9.85 usec
PLW1         28.63999939 W
SFO2         400.1316005 MHz
NUC2                  1H
CPDPRG[2          waltz65
PCPD2              90.00 usec
PLW2          8.47000027 W
PLW12         0.23528001 W
PLW13         0.11834000 W

F2 - Processing parameters
SI                 32768
SF           100.6127608 MHz
WDW                   EM
SSB      0
LB                  1.00 Hz
GB       0
PC                  1.40
```

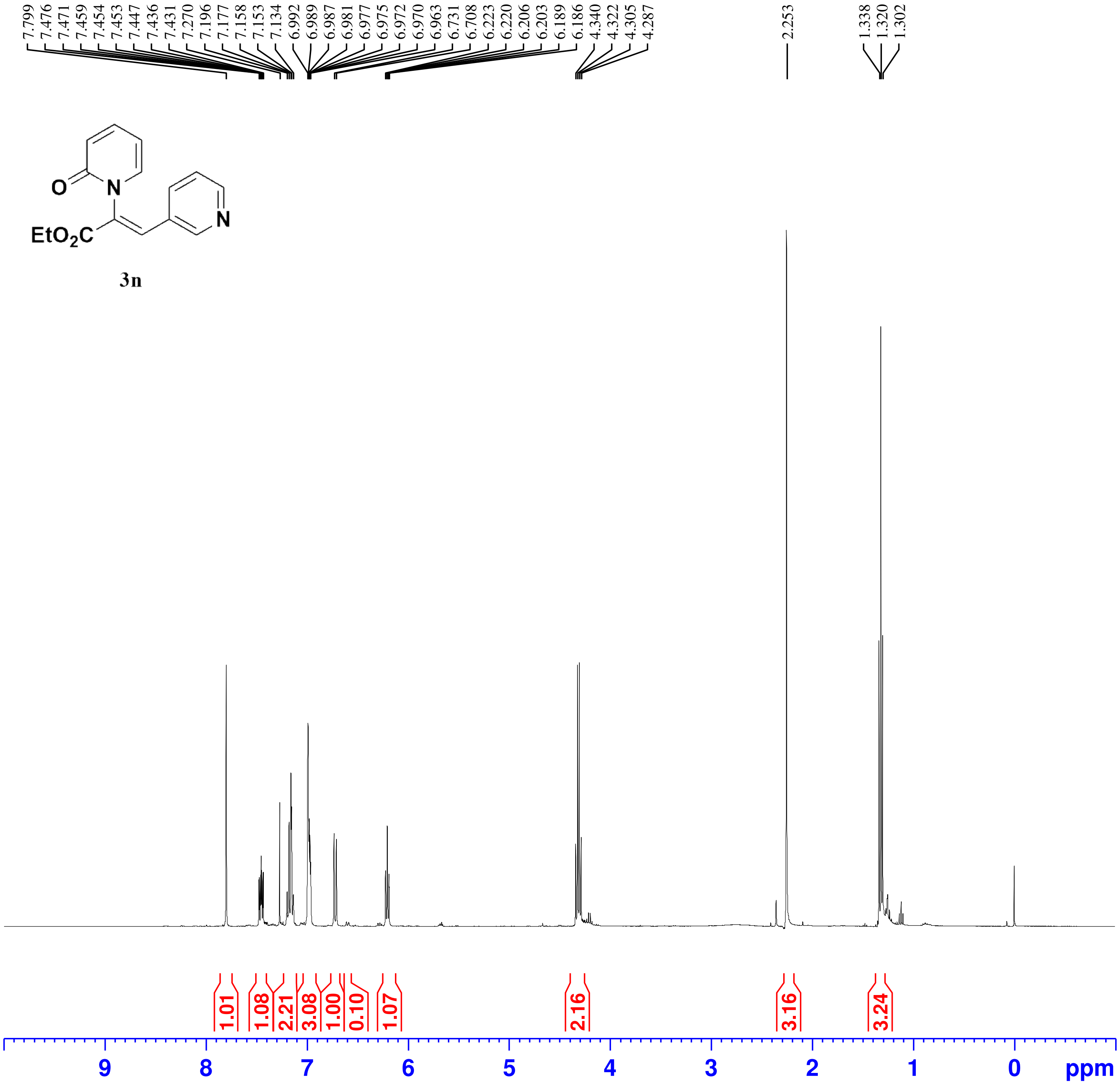

BRUKER

Current Data Parameters

NAME	1H gns-11-122
EXPNO	1
PROCNO	1

F2 - Acquisition Parameters

Date_	20210114
Time	15.34 h
INSTRUM	spect
PROBHD	Z104450_0192 (
PULPROG	zg30
TD	65536
SOLVENT	CDCl3
NS	16
DS	2
SWH	8012.820 Hz
FIDRES	0.244532 Hz
AQ	4.0894465 sec
RG	161
DW	62.400 usec
DE	16.92 usec
TE	298.0 K
D1	1.00000000 sec
TD0	1
SFO1	400.1324708 MHz
NUC1	1H
P0	5.00 usec
P1	15.00 usec
PLW1	8.47000027 W

F2 - Processing parameters

SI	65536
SF	400.1300058 MHz
WDW	EM
SSB	0
LB	0.30 Hz
GB	0
PC	1.00

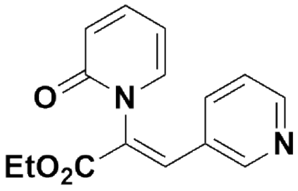

3n

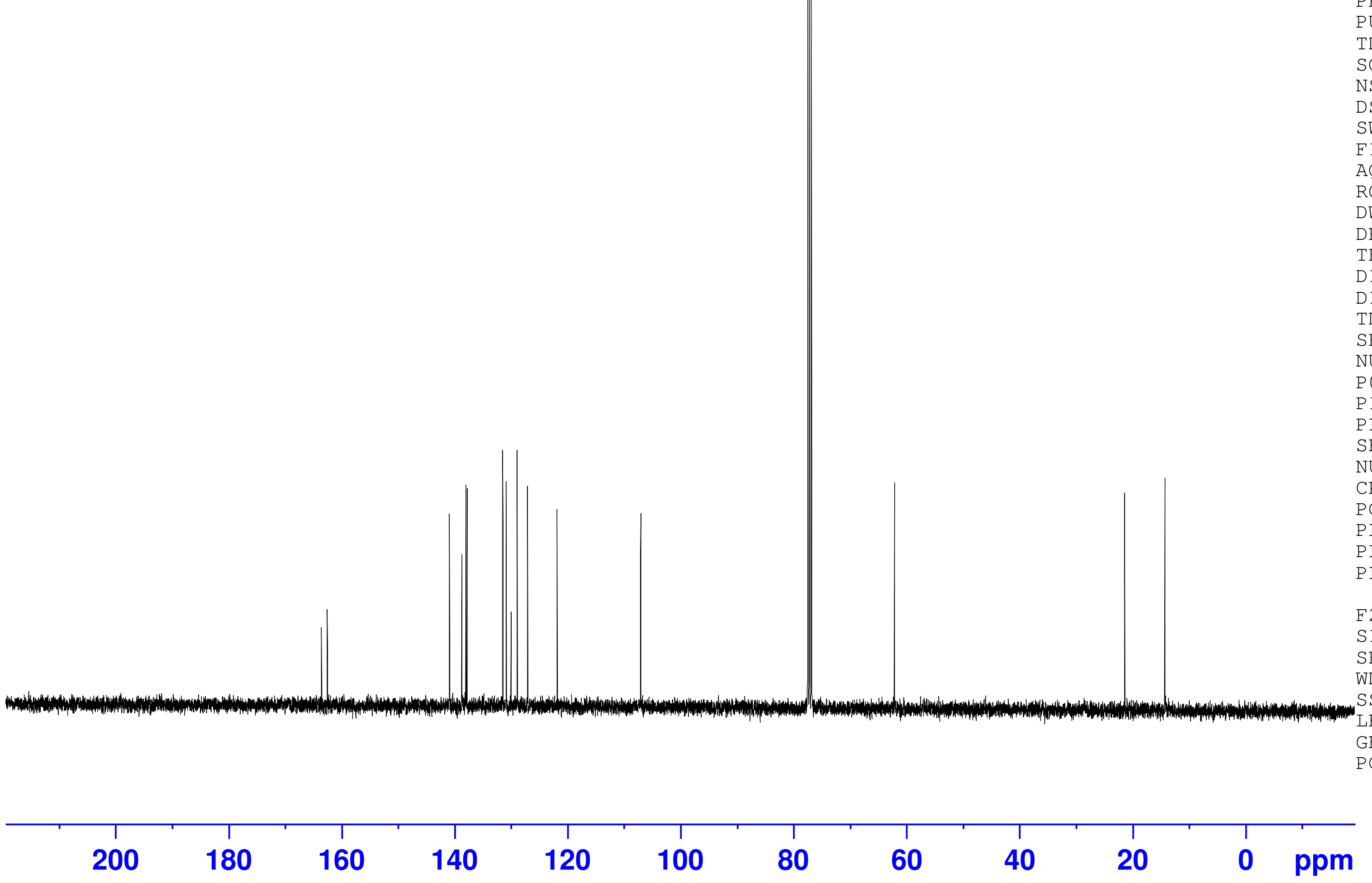

Current Data Parameters
NAME 13C gns-11-122
EXPNO 1
PROCNO 1

F2 - Acquisition Parameters
Date_ 20210114
Time 15.48 h
INSTRUM spect
PROBHD Z104450_0192 (
PULPROG zgpg30
TD 65536
SOLVENT CDCl3
NS 200
DS 2
SWH 24038.461 Hz
FIDRES 0.733596 Hz
AQ 1.3631488 sec
RG 287
DW 20.800 usec
DE 6.50 usec
TE 298.3 K
D1 2.00000000 sec
D11 0.03000000 sec
TD0 1
SFO1 100.6228298 MHz
NUC1 13C
P0 3.28 usec
P1 9.85 usec
PLW1 28.63999939 W
SFO2 400.1316005 MHz
NUC2 1H
CPDPRG[2 waltz65
PCPD2 90.00 usec
PLW2 8.47000027 W
PLW12 0.23528001 W
PLW13 0.11834000 W

F2 - Processing parameters
SI 32768
SF 100.6127584 MHz
WDW EM
SSB 0
LB 1.00 Hz
GB 0
PC 1.40

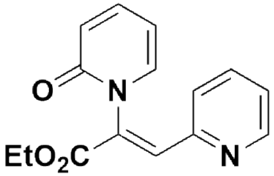

3o

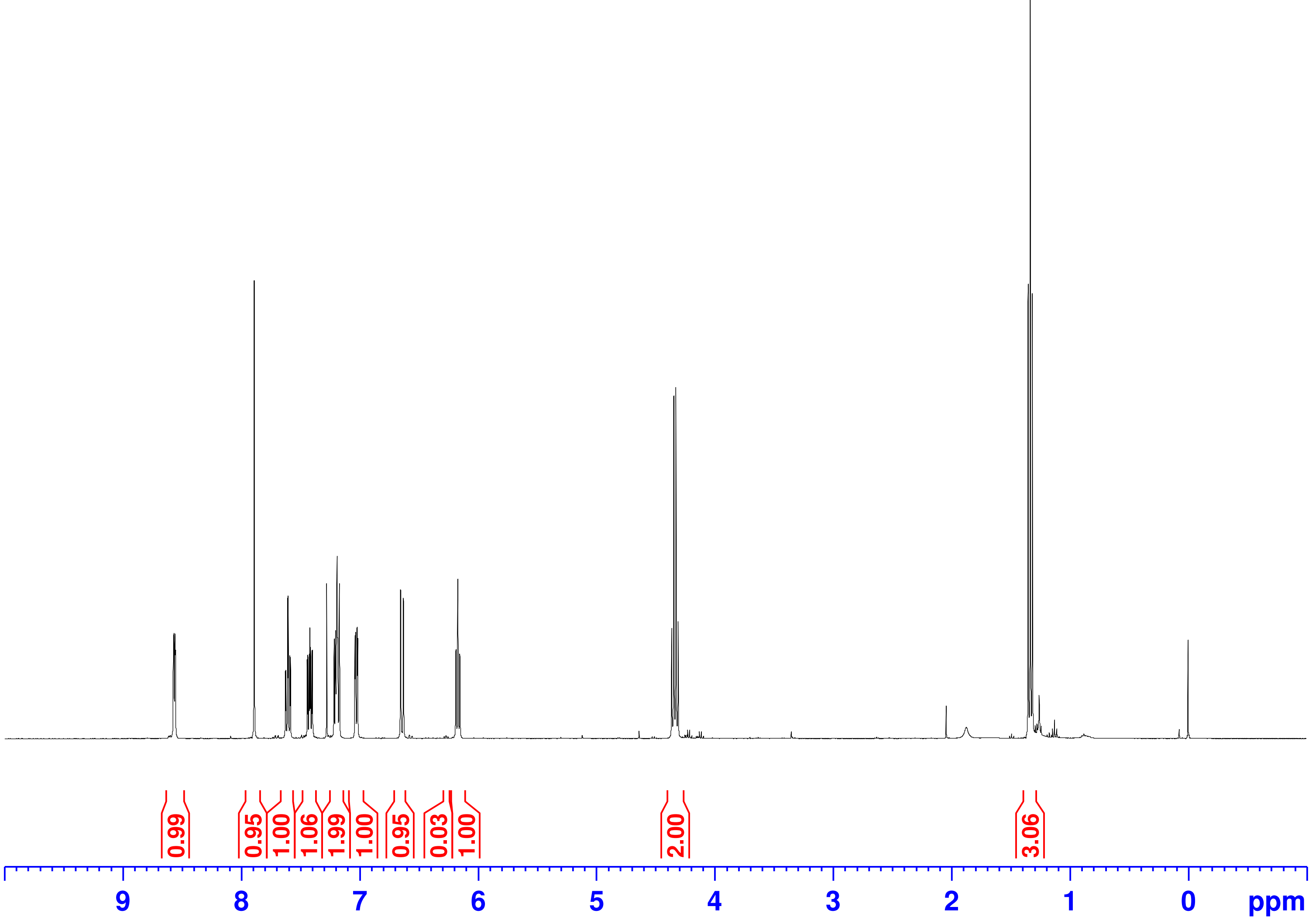


```
Current Data Parameters
NAME       1H gns-11-114
EXPNO                  1
PROCNO                 1

F2 - Acquisition Parameters
Date_           20210112
Time               17.53 h
INSTRUM            spect
PROBHD    Z104450_0192 (
PULPROG             zg30
TD                 65536
SOLVENT            CDCl3
NS                    16
DS                     2
SWH             8012.820 Hz
FIDRES          0.244532 Hz
AQ             4.0894465 sec
RG                   161
DW                62.400 usec
DE                 16.92 usec
TE                 298.0 K
D1            1.00000000 sec
TD0                    1
SFO1         400.1324708 MHz
NUC1                  1H
P0                  5.00 usec
P1                 15.00 usec
PLW1          8.47000027 W

F2 - Processing parameters
SI                 65536
SF           400.1300026 MHz
WDW                   EM
SSB      0
LB                  0.30 Hz
GB       0
PC                  1.00
```

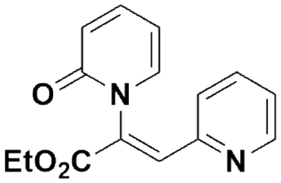

3o

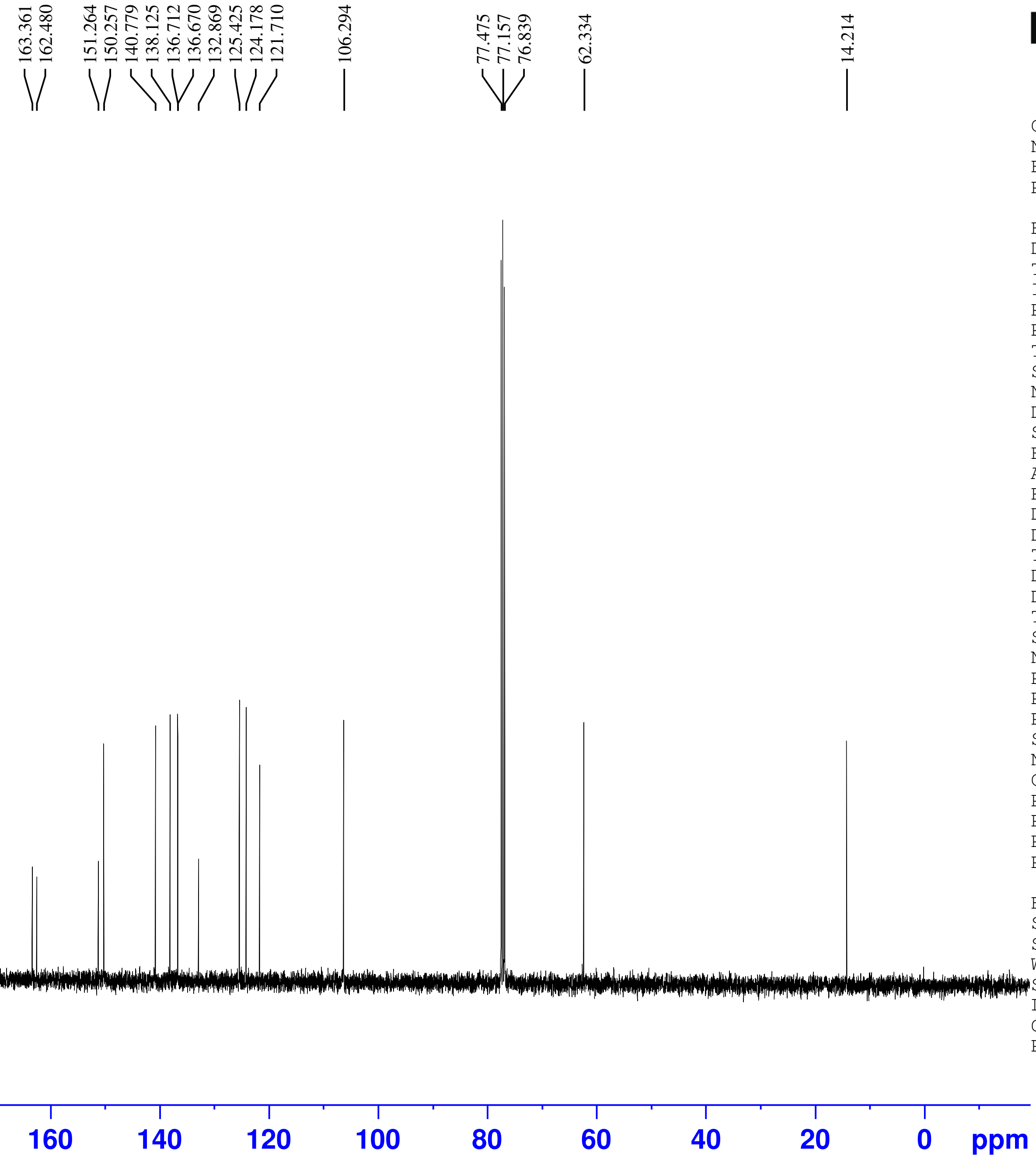

BRUKER

Current Data Parameters
NAME 13C gns-11-114
EXPNO 1
PROCNO 1

F2 - Acquisition Parameters
Date_ 20210112
Time 18.01 h
INSTRUM spect
PROBHD Z104450_0192 (
PULPROG zgpg30
TD 65536
SOLVENT CDCl3
NS 100
DS 2
SWH 24038.461 Hz
FIDRES 0.733596 Hz
AQ 1.3631488 sec
RG 203
DW 20.800 usec
DE 6.50 usec
TE 298.1 K
D1 2.00000000 sec
D11 0.03000000 sec
TD0 1
SFO1 100.6228298 MHz
NUC1 13C
P0 3.28 usec
P1 9.85 usec
PLW1 28.63999939 W
SFO2 400.1316005 MHz
NUC2 1H
CPDPRG[2 waltz65
PCPD2 90.00 usec
PLW2 8.47000027 W
PLW12 0.23528001 W
PLW13 0.11834000 W

F2 - Processing parameters
SI 32768
SF 100.6127586 MHz
WDW EM
SSB 0
LB 1.00 Hz
GB 0
PC 1.40

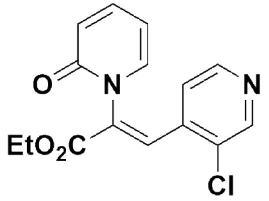

3p

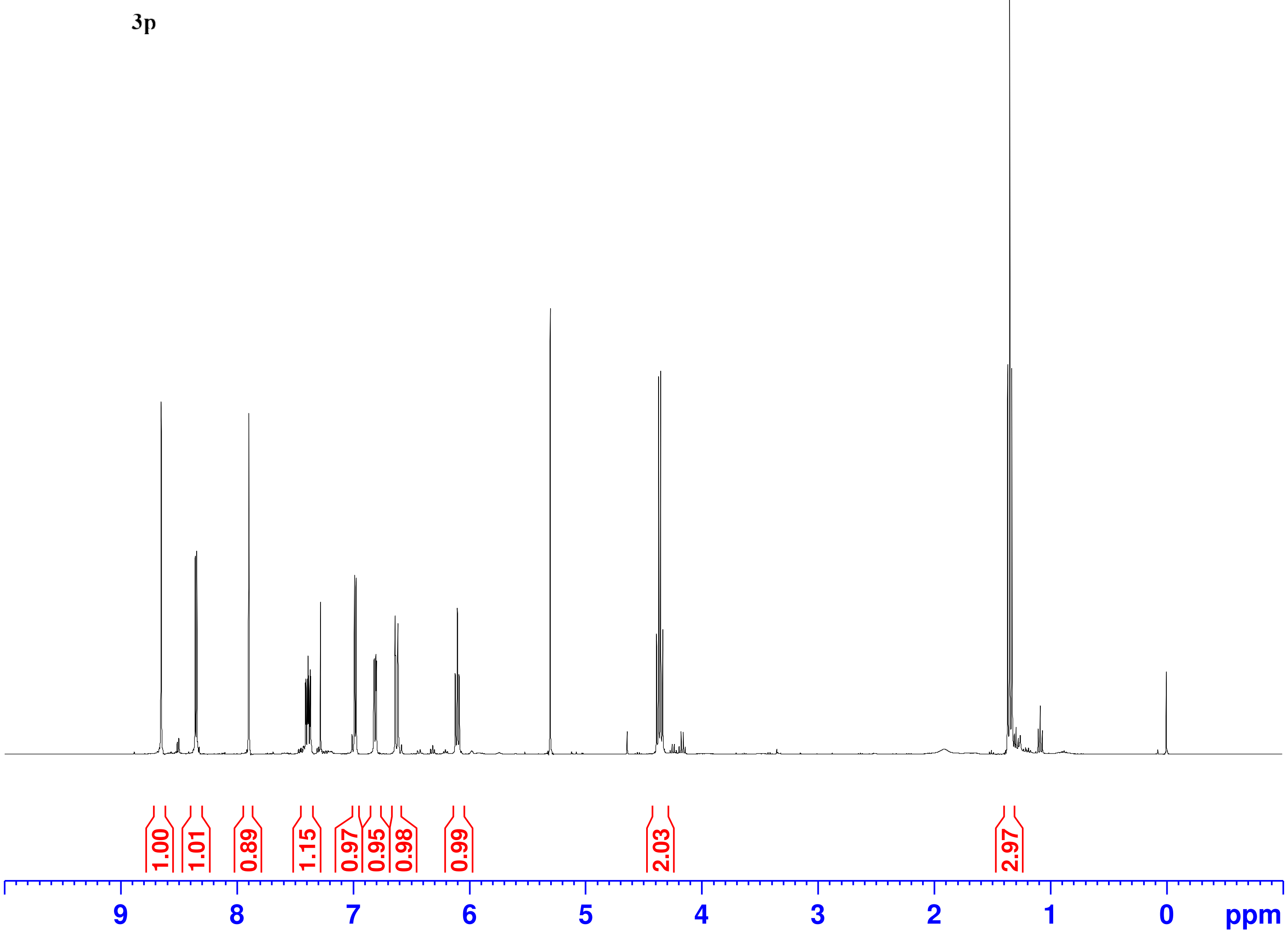

BRUKER

Current Data Parameters
NAME 1H gns-11-116 2
EXPNO 1
PROCNO 1

F2 - Acquisition Parameters
Date_ 20210114
Time 15.28 h
INSTRUM spect
PROBHD Z104450_0192 (
PULPROG zg30
TD 65536
SOLVENT CDCl3
NS 16
DS 2
SWH 8012.820 Hz
FIDRES 0.244532 Hz
AQ 4.0894465 sec
RG 161
DW 62.400 usec
DE 16.92 usec
TE 298.0 K
D1 1.00000000 sec
TD0 1
SFO1 400.1324708 MHz
NUC1 1H
P0 5.00 usec
P1 15.00 usec
PLW1 8.47000027 W

F2 - Processing parameters
SI 65536
SF 400.1300019 MHz
WDW EM
SSB 0
LB 0.30 Hz
GB 0
PC 1.00

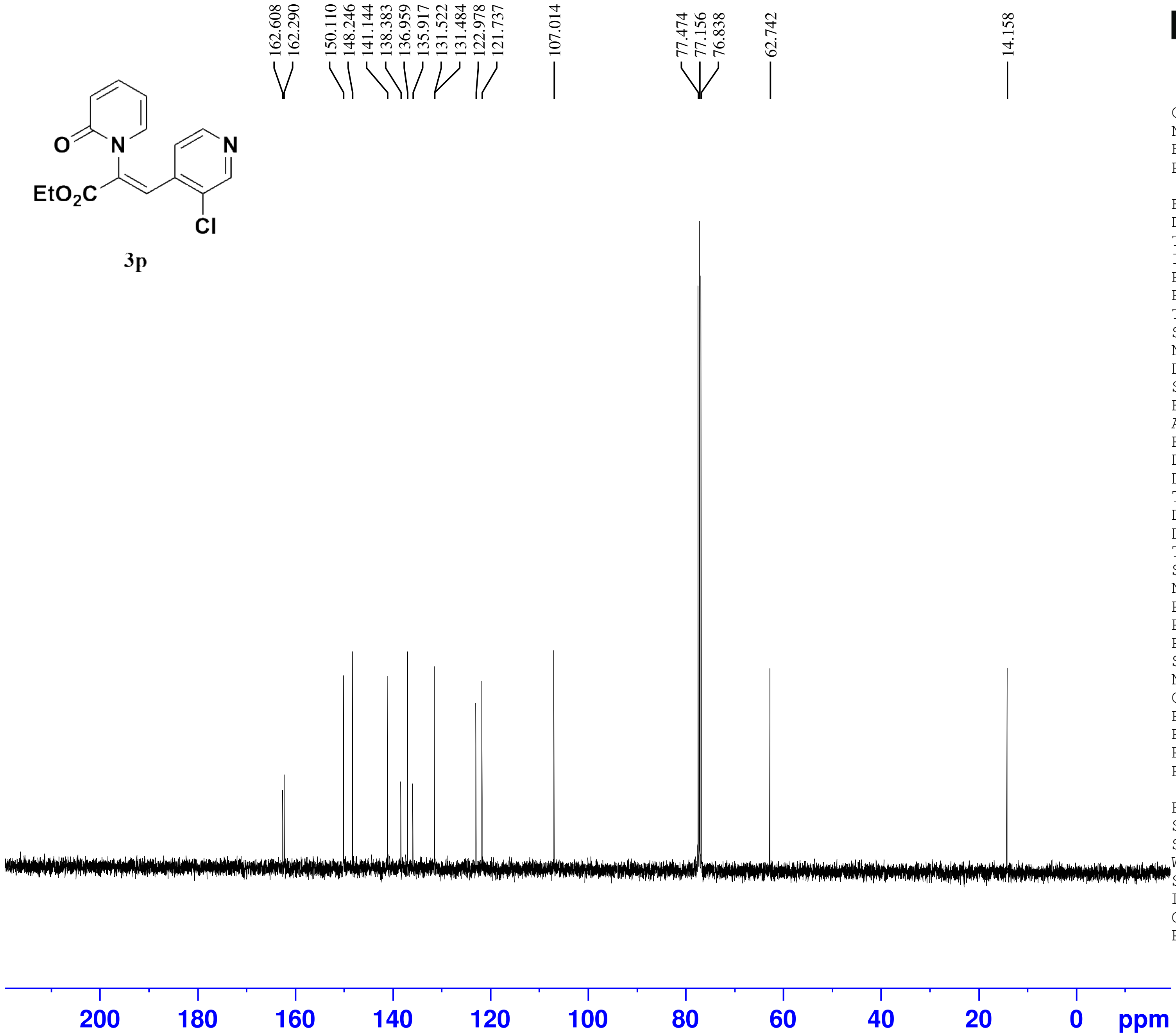

BRUKER

```
Current Data Parameters
NAME       13C gns-11-116
EXPNO                   1
PROCNO                  1

F2 - Acquisition Parameters
Date_            20210112
Time                17.45 h
INSTRUM             spect
PROBHD   Z104450_0192 (
PULPROG           zgpg30
TD                 65536
SOLVENT            CDCl3
NS                   100
DS                     2
SWH            24038.461 Hz
FIDRES          0.733596 Hz
AQ             1.3631488 sec
RG                   203
DW                20.800 usec
DE                  6.50 usec
TE                 298.1 K
D1            2.00000000 sec
D11           0.03000000 sec
TD0                    1
SFO1         100.6228298 MHz
NUC1                 13C
P0                  3.28 usec
P1                  9.85 usec
PLW1         28.63999939 W
SFO2         400.1316005 MHz
NUC2                  1H
CPDPRG[2         waltz65
PCPD2              90.00 usec
PLW2          8.47000027 W
PLW12         0.23528001 W
PLW13         0.11834000 W

F2 - Processing parameters
SI                 32768
SF           100.6127586 MHz
WDW                   EM
SSB      0
LB                  1.00 Hz
GB       0
PC                  1.40
```

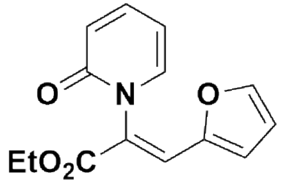

3q

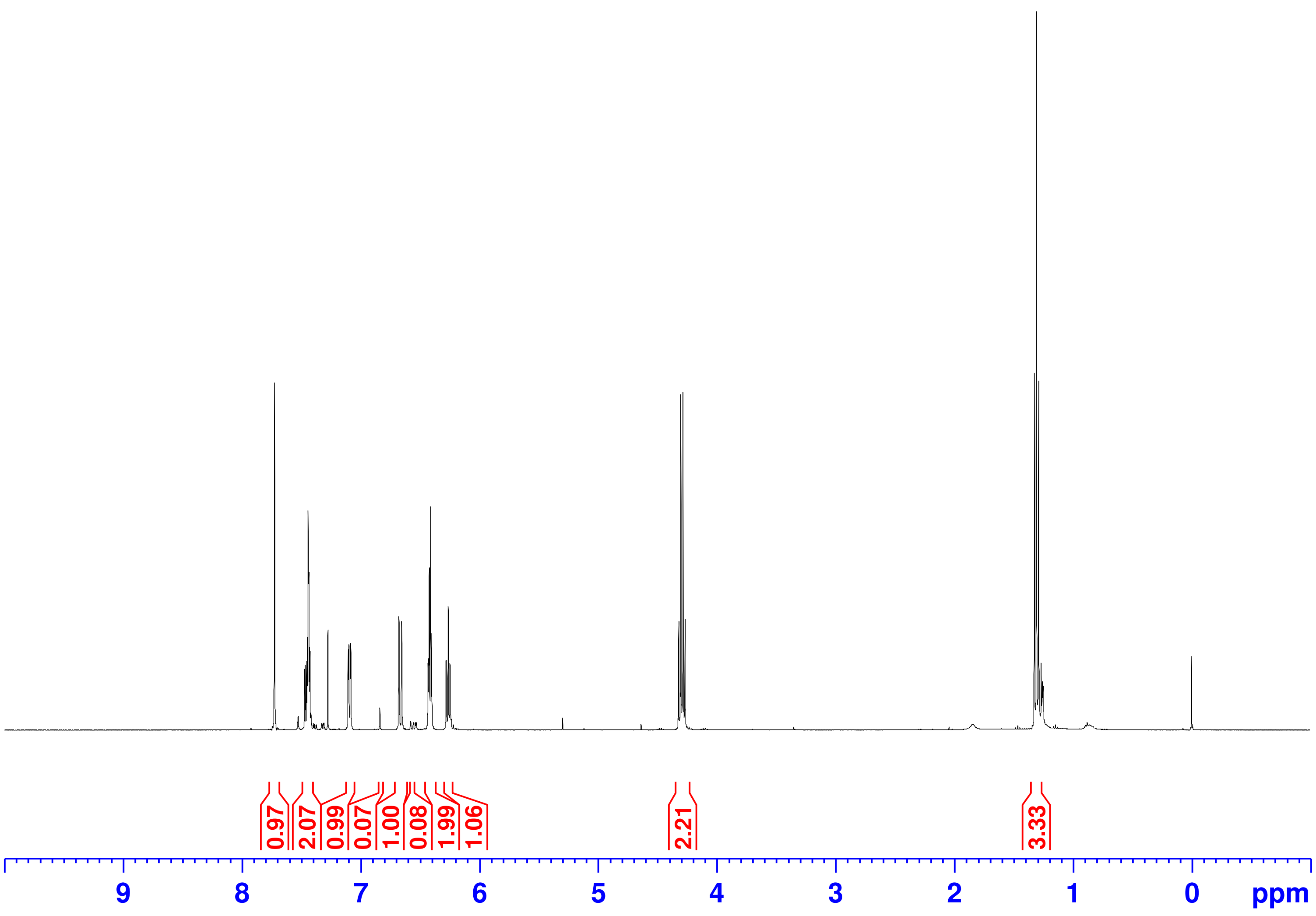


```
Current Data Parameters
NAME        1H gns-11-104
EXPNO                   1
PROCNO                  1

F2 - Acquisition Parameters
Date_            20210106
Time                12.46 h
INSTRUM             spect
PROBHD   Z104450_0192 (
PULPROG              zg30
TD                  65536
SOLVENT             CDCl3
NS                     16
DS                      2
SWH              8012.820 Hz
FIDRES           0.244532 Hz
AQ              4.0894465 sec
RG                    144
DW                 62.400 usec
DE                  16.92 usec
TE                  297.2 K
D1             1.00000000 sec
TD0                     1
SFO1          400.1324708 MHz
NUC1                   1H
P0                   5.00 usec
P1                  15.00 usec
PLW1           8.47000027 W

F2 - Processing parameters
SI                  65536
SF            400.1300032 MHz
WDW                    EM
SSB      0
LB                   0.30 Hz
GB       0
PC                   1.00
```

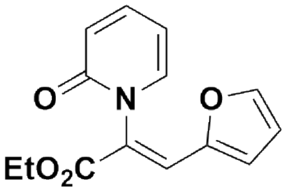

3q

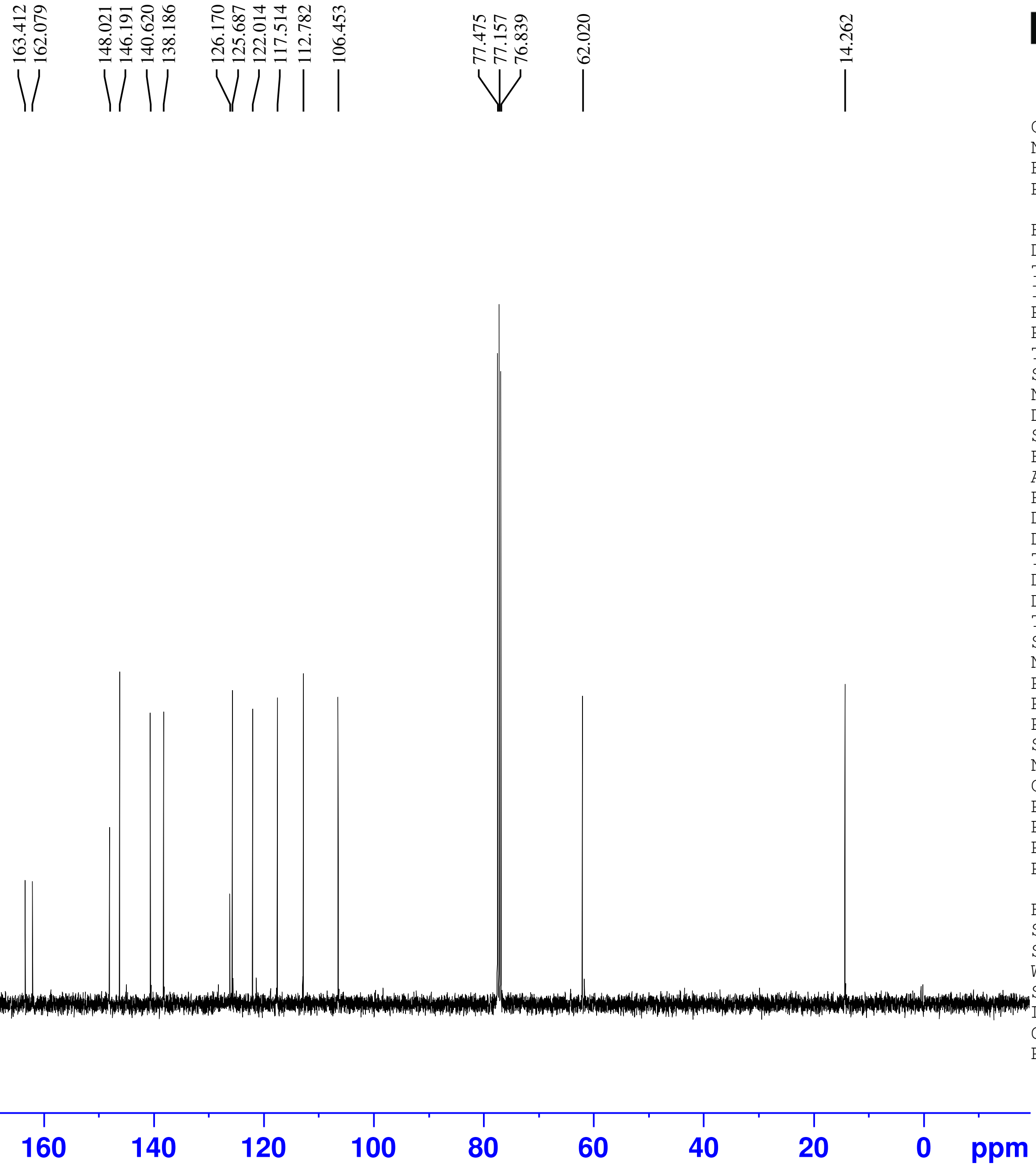

BRUKER

```
Current Data Parameters
NAME      13C gns-11-104
EXPNO                  1
PROCNO                 1

F2 - Acquisition Parameters
Date_           20210106
Time               12.54 h
INSTRUM            spect
PROBHD   Z104450_0192 (
PULPROG          zgpg30
TD                 65536
SOLVENT            CDCl3
NS                   100
DS                     2
SWH            24038.461 Hz
FIDRES          0.733596 Hz
AQ             1.3631488 sec
RG                   161
DW                20.800 usec
DE                  6.50 usec
TE                 297.9 K
D1            2.00000000 sec
D11           0.03000000 sec
TD0                    1
SFO1         100.6228298 MHz
NUC1                 13C
P0                  3.28 usec
P1                  9.85 usec
PLW1         28.63999939 W
SFO2         400.1316005 MHz
NUC2                  1H
CPDPRG[2         waltz65
PCPD2              90.00 usec
PLW2          8.47000027 W
PLW12         0.23528001 W
PLW13         0.11834000 W

F2 - Processing parameters
SI                 32768
SF           100.6127593 MHz
WDW                   EM
SSB      0
LB                  1.00 Hz
GB       0
PC                  1.40
```

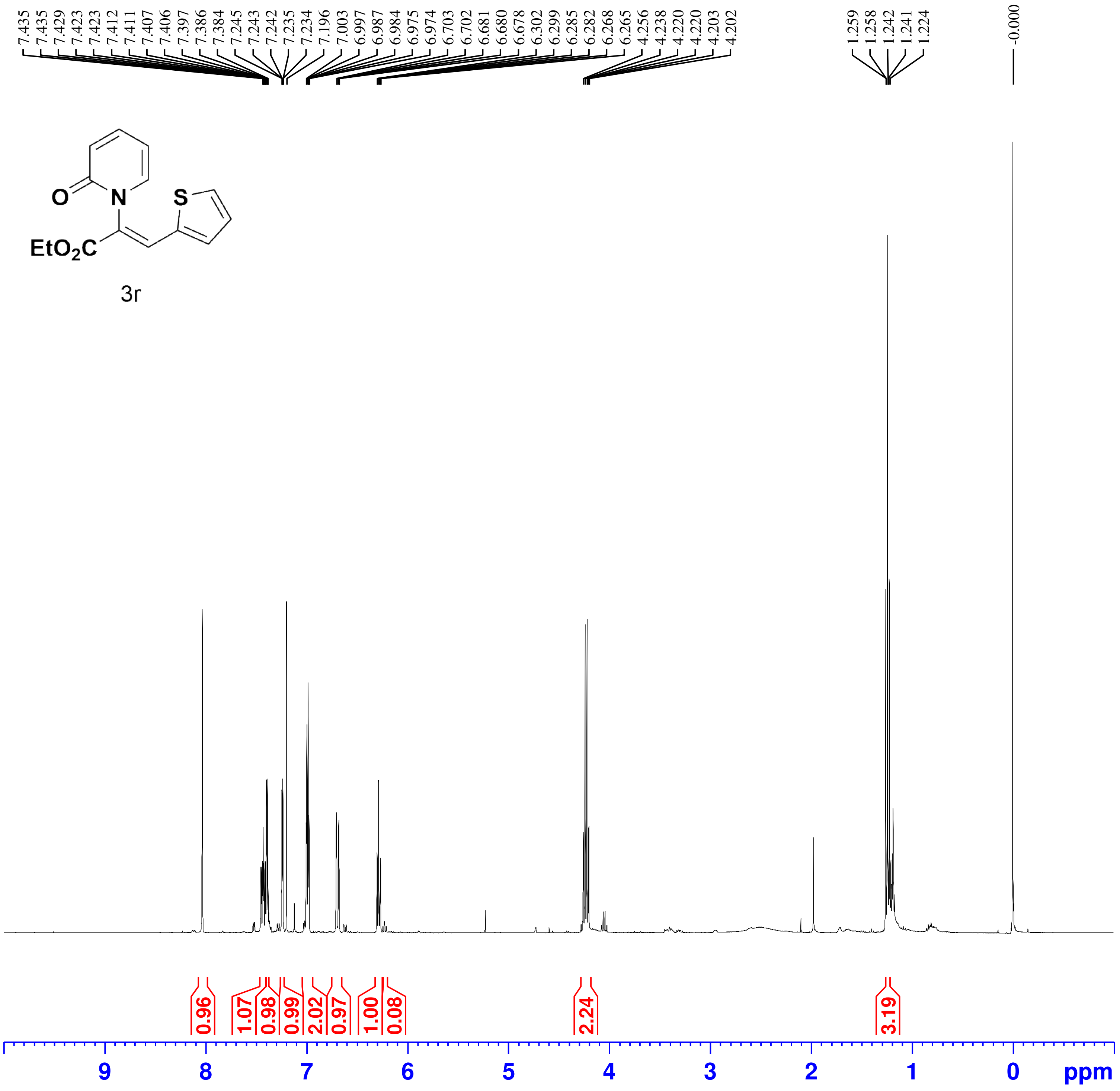

Current Data Parameters
NAME 1H gns-13-038
EXPNO 1
PROCNO 1

F2 - Acquisition Parameters
Date_ 20210617
Time 10.42 h
INSTRUM Avance
PROBHD Z167430_0032 (
PULPROG zg30
TD 65536
SOLVENT CDCl3
NS 16
DS 2
SWH 8196.722 Hz
FIDRES 0.250144 Hz
AQ 3.9976959 sec
RG 101
DW 61.000 usec
DE 13.20 usec
TE 298.0 K
D1 0.01000000 sec
TD0 1
SFO1 400.3024719 MHz
NUC1 1H
P0 4.00 usec
P1 12.00 usec
PLW1 8.80000019 W

F2 - Processing parameters
SI 65536
SF 400.3000337 MHz
WDW no
SSB 0
LB 0 Hz
GB 0
PC 1.00

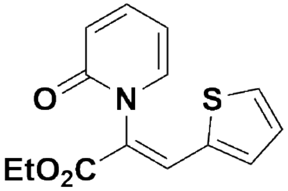

3r

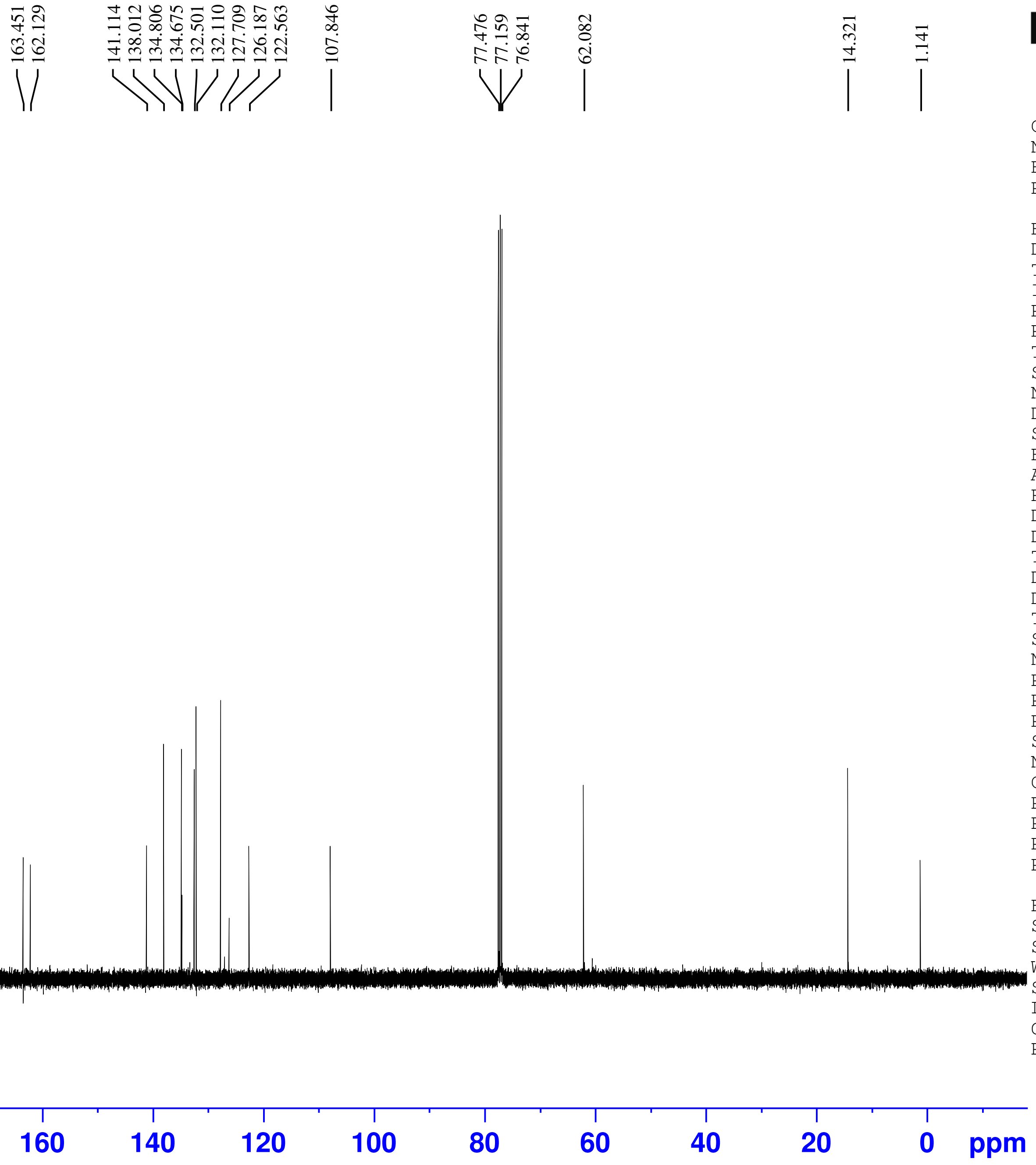

BRUKER

Current Data Parameters
NAME 13C gns-13-038
EXPNO 1
PROCNO 1

F2 - Acquisition Parameters
Date_ 20210617
Time 10.49 h
INSTRUM Avance
PROBHD Z167430_0032 (
PULPROG zgpg30
TD 65536
SOLVENT CDCl3
NS 100
DS 4
SWH 23809.523 Hz
FIDRES 0.726609 Hz
AQ 1.3762560 sec
RG 3.25
DW 21.000 usec
DE 19.29 usec
TE 298.0 K
D1 2.00000000 sec
D11 0.03000000 sec
TD0 1
SFO1 100.6655806 MHz
NUC1 13C
P0 3.33 usec
P1 10.00 usec
PLW1 39.31399918 W
SFO2 400.3016012 MHz
NUC2 1H
CPDPRG[2 waltz64
PCPD2 80.00 usec
PLW2 8.80000019 W
PLW12 0.20176961 W
PLW13 0.10112690 W

F2 - Processing parameters
SI 131072
SF 100.6555030 MHz
WDW no
SSB 0
LB 0 Hz
GB 0
PC 1.40

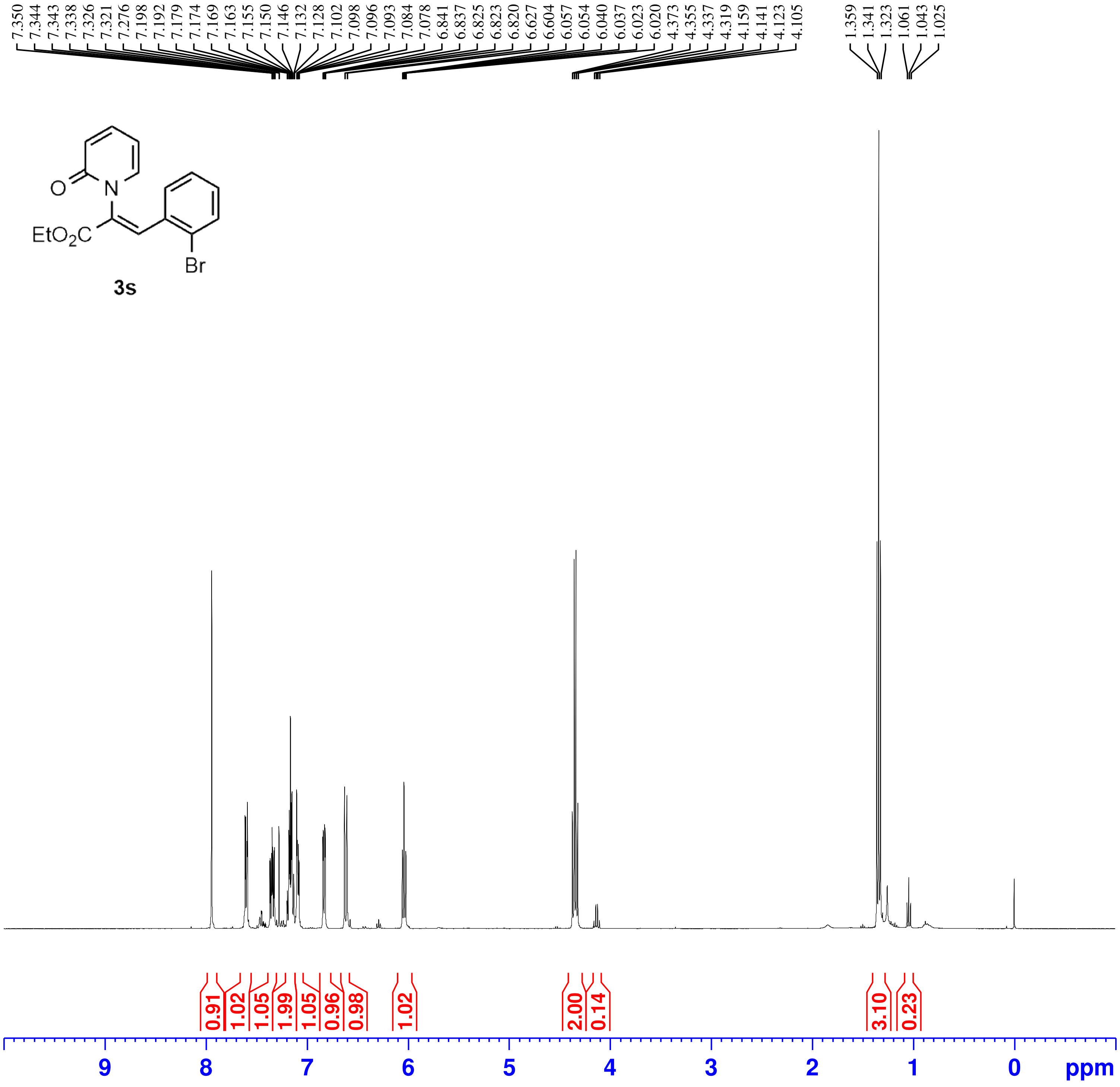

Current Data Parameters
NAME 1H gns-11-094 3
EXPNO 1
PROCNO 1

F2 - Acquisition Parameters
Date_ 20210110
Time 12.19 h
INSTRUM spect
PROBHD Z104450_0192 (
PULPROG zg30
TD 65536
SOLVENT CDCl3
NS 16
DS 2
SWH 8012.820 Hz
FIDRES 0.244532 Hz
AQ 4.0894465 sec
RG 128
DW 62.400 usec
DE 16.92 usec
TE 297.0 K
D1 1.00000000 sec
TD0 1
SFO1 400.1324708 MHz
NUC1 1H
P0 5.00 usec
P1 15.00 usec
PLW1 8.47000027 W

F2 - Processing parameters
SI 65536
SF 400.1300032 MHz
WDW EM
SSB 0
LB 0.30 Hz
GB 0
PC 1.00

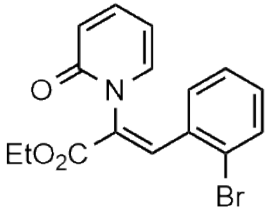

3s

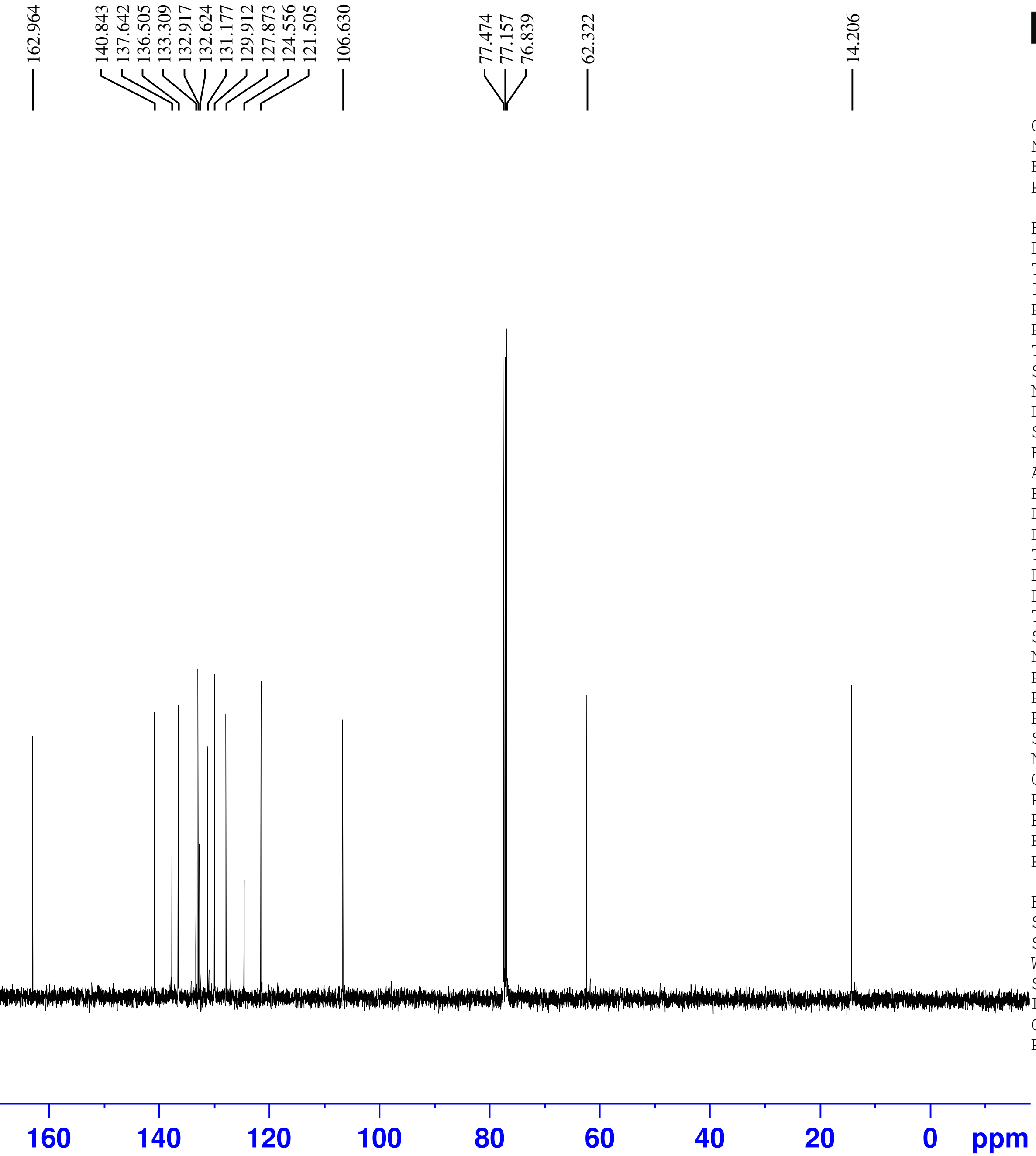

BRUKER

```
Current Data Parameters
NAME       13C gns-11-094 3
EXPNO                    1
PROCNO                   1

F2 - Acquisition Parameters
Date_             20210110
Time                 12.27 h
INSTRUM              spect
PROBHD    Z104450_0192 (
PULPROG            zgpg30
TD                  65536
SOLVENT             CDCl3
NS                    100
DS                      2
SWH            24038.461 Hz
FIDRES          0.733596 Hz
AQ             1.3631488 sec
RG                    203
DW                 20.800 usec
DE                   6.50 usec
TE                  297.7 K
D1             2.00000000 sec
D11            0.03000000 sec
TD0                     1
SFO1          100.6228298 MHz
NUC1                  13C
P0                   3.28 usec
P1                   9.85 usec
PLW1          28.63999939 W
SFO2          400.1316005 MHz
NUC2                   1H
CPDPRG[2          waltz65
PCPD2               90.00 usec
PLW2           8.47000027 W
PLW12          0.23528001 W
PLW13          0.11834000 W

F2 - Processing parameters
SI                  32768
SF            100.6127597 MHz
WDW                    EM
SSB        0
LB                   1.00 Hz
GB         0
PC                   1.40
```

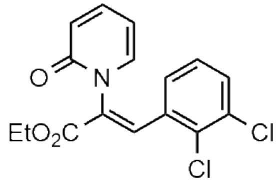

3t

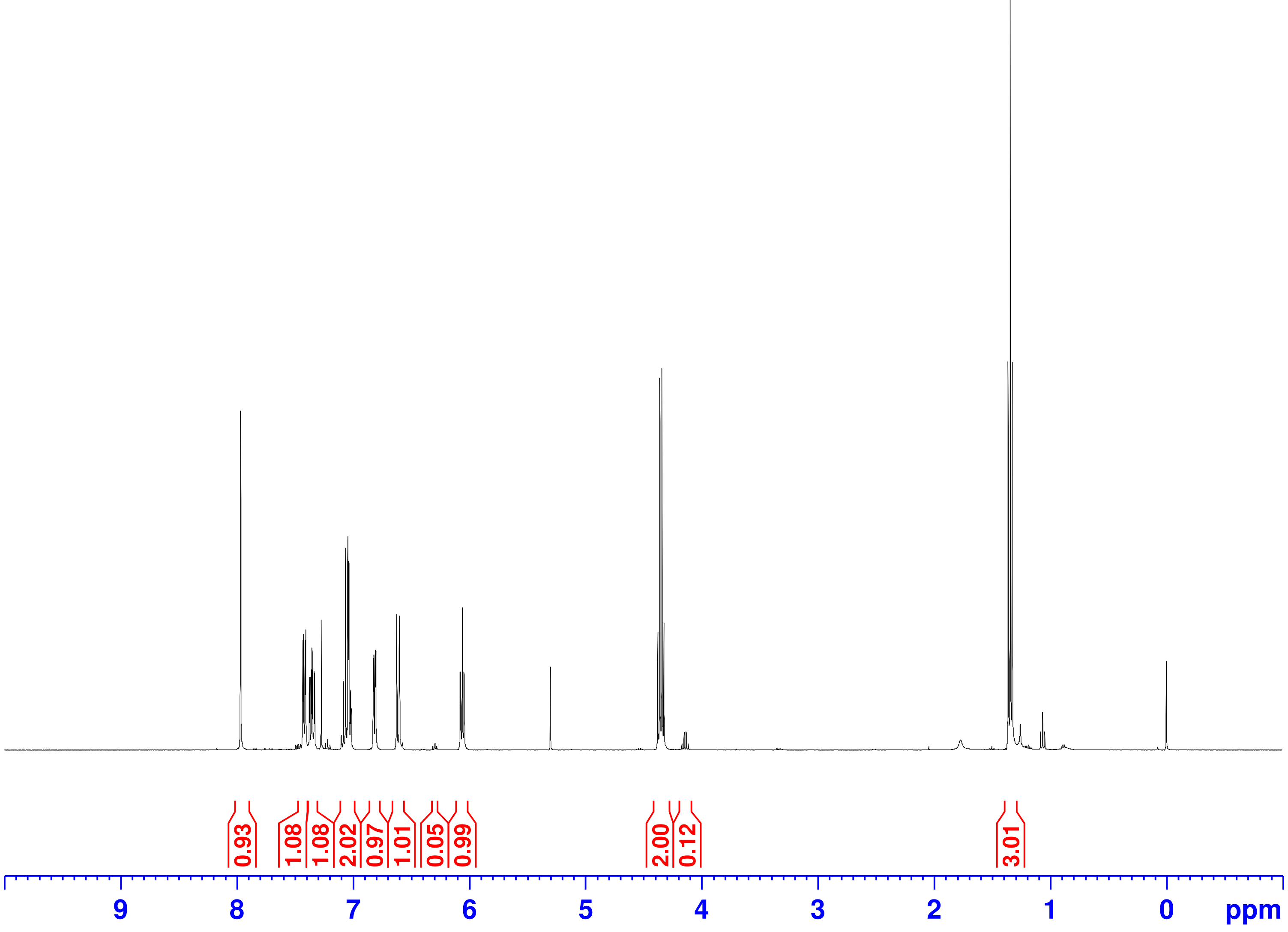

BRUKER

Current Data Parameters
NAME 1H gns-11-120
EXPNO 1
PROCNO 1

F2 - Acquisition Parameters
Date_ 20210112
Time 18.26 h
INSTRUM spect
PROBHD Z104450_0192 (
PULPROG zg30
TD 65536
SOLVENT CDCl3
NS 16
DS 2
SWH 8012.820 Hz
FIDRES 0.244532 Hz
AQ 4.0894465 sec
RG 161
DW 62.400 usec
DE 16.92 usec
TE 298.0 K
D1 1.00000000 sec
TD0 1
SFO1 400.1324708 MHz
NUC1 1H
P0 5.00 usec
P1 15.00 usec
PLW1 8.47000027 W

F2 - Processing parameters
SI 65536
SF 400.1300042 MHz
WDW EM
SSB 0
LB 0.30 Hz
GB 0
PC 1.00

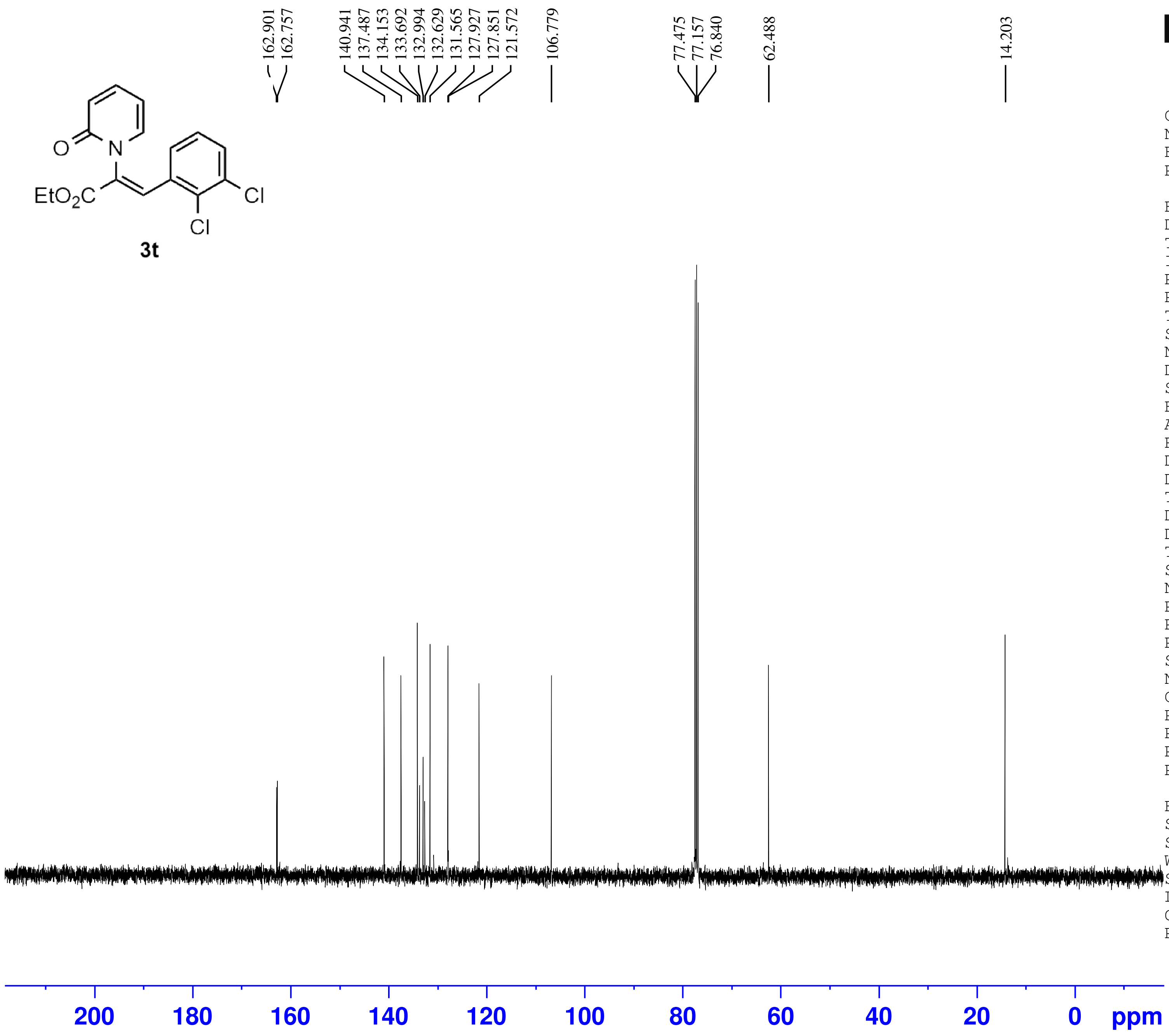

BRUKER

Current Data Parameters

NAME	13C gns-11-120
EXPNO	1
PROCNO	1

F2 - Acquisition Parameters

Date_	20210112
Time	18.35 h
INSTRUM	spect
PROBHD	Z104450_0192 (
PULPROG	zgpg30
TD	65536
SOLVENT	CDCl3
NS	100
DS	2
SWH	24038.461 Hz
FIDRES	0.733596 Hz
AQ	1.3631488 sec
RG	203
DW	20.800 usec
DE	6.50 usec
TE	298.1 K
D1	2.00000000 sec
D11	0.03000000 sec
TD0	1
SFO1	100.6228298 MHz
NUC1	13C
P0	3.28 usec
P1	9.85 usec
PLW1	28.63999939 W
SFO2	400.1316005 MHz
NUC2	1H
CPDPRG[2	waltz65
PCPD2	90.00 usec
PLW2	8.47000027 W
PLW12	0.23528001 W
PLW13	0.11834000 W

F2 - Processing parameters

SI	32768
SF	100.6127579 MHz
WDW	EM
SSB	0
LB	1.00 Hz
GB	0
PC	1.40

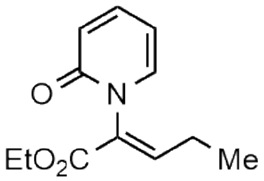

3u

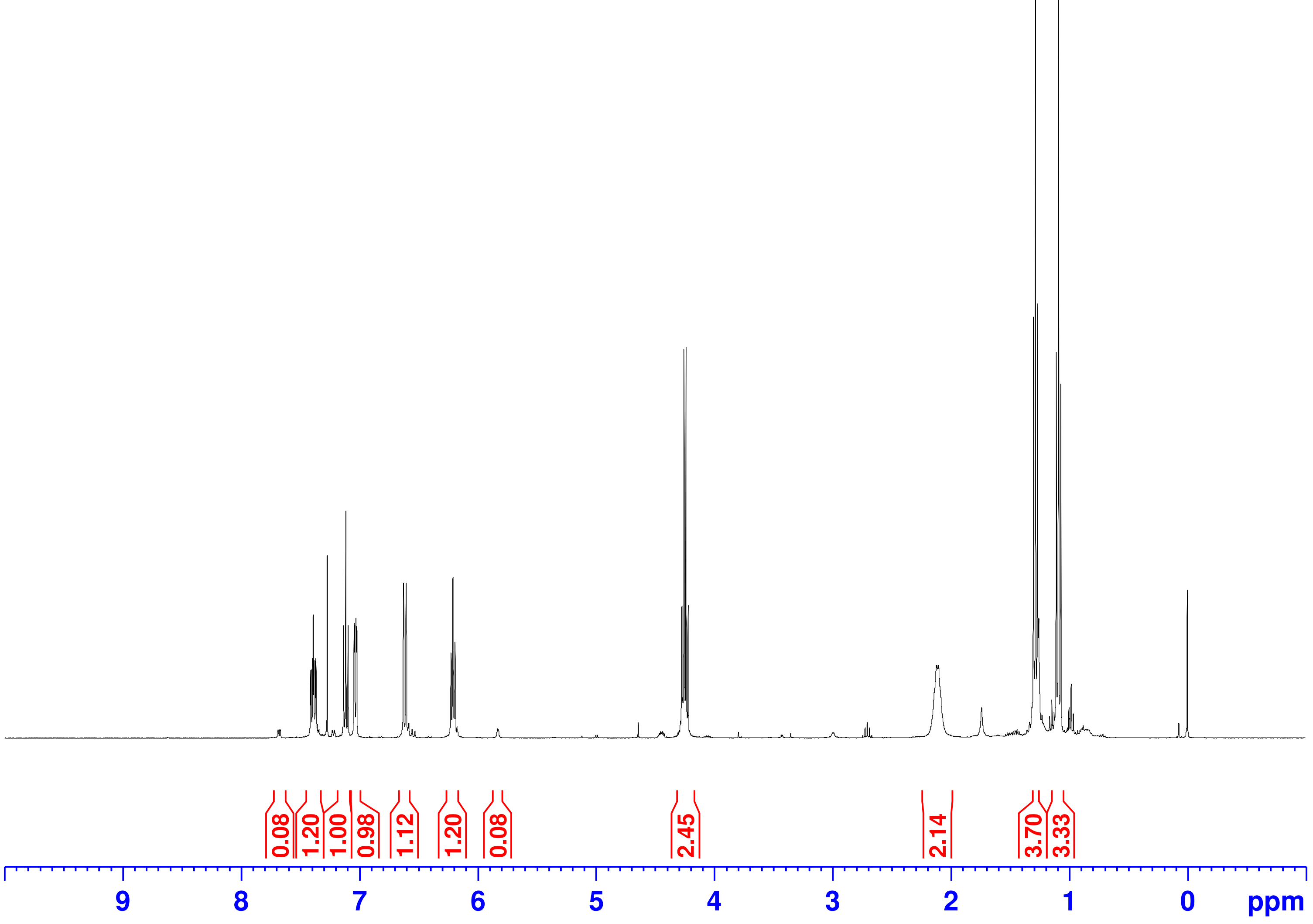

Current Data Parameters
NAME 1H gns-12-012 2
EXPNO 1
PROCNO 1

F2 - Acquisition Parameters
Date_ 20210220
Time 15.36
INSTRUM spect
PROBHD 5 mm Multinucl
PULPROG zg30
TD 32768
SOLVENT CDCl3
NS 16
DS 0
SWH 8012.820 Hz
FIDRES 0.244532 Hz
AQ 2.0447233 sec
RG 181
DW 62.400 usec
DE 6.50 usec
TE 295.2 K
D1 0.01000000 sec
TD0 1

======== CHANNEL f1 ========
NUC1 1H
P1 7.20 usec
PL1 -5.00 dB
SFO1 400.1332010 MHz

F2 - Processing parameters
SI 131072
SF 400.1300045 MHz
WDW EM
SSB 0
LB 0.25 Hz
GB 0
PC 0.20

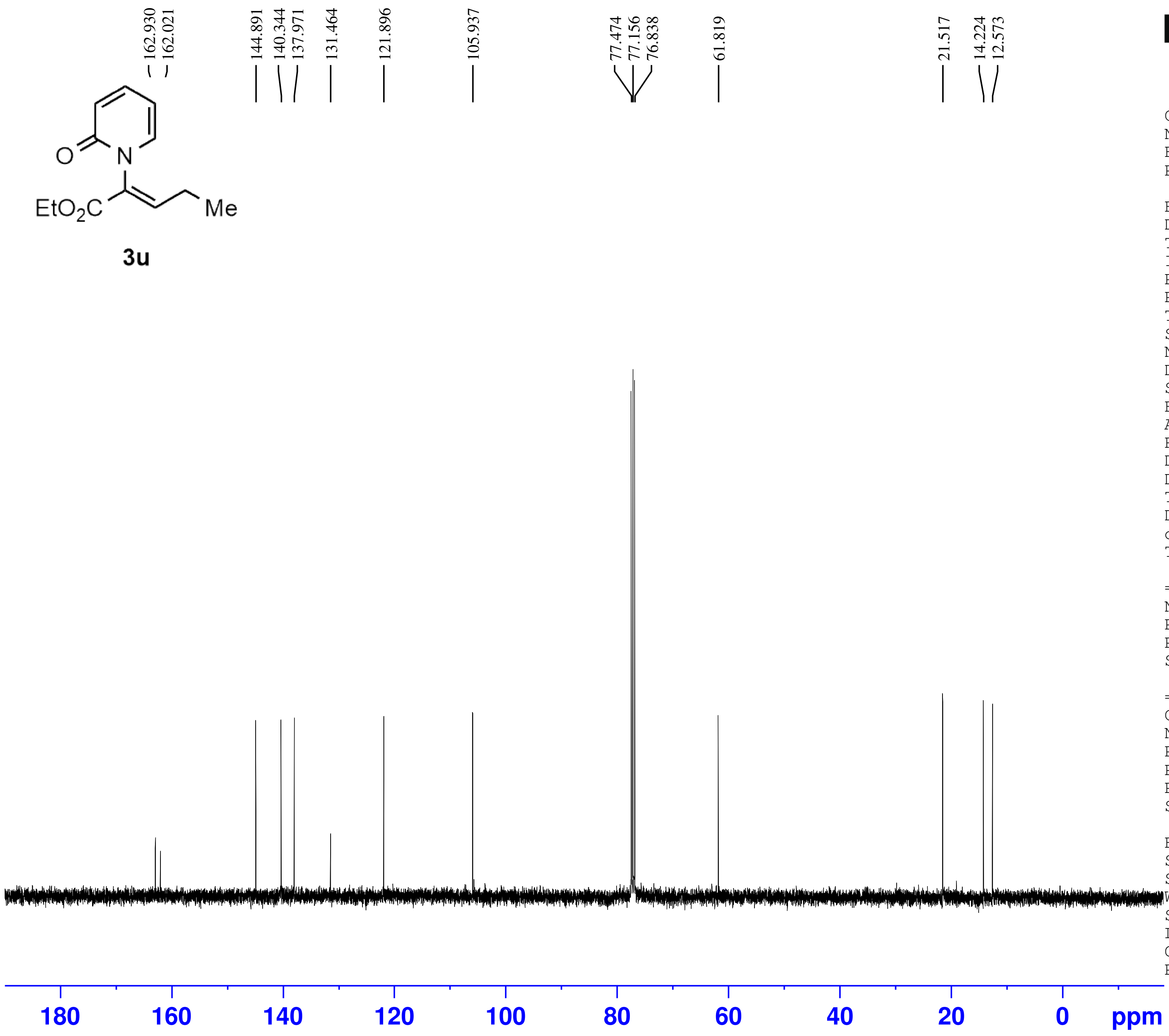


```
Current Data Parameters
NAME        13C gns-12-012 3
EXPNO                      1
PROCNO                     1

F2 - Acquisition Parameters
Date_              20210226
Time                  15.28
INSTRUM               spect
PROBHD     5 mm Multinucl
PULPROG             zgdc30
TD                   65536
SOLVENT              CDCl3
NS                     300
DS                       4
SWH              26246.719 Hz
FIDRES            0.400493 Hz
AQ               1.2484608 sec
RG                   812.7
DW                  19.050 usec
DE                    6.50 usec
TE                   295.2 K
D1              0.69999999 sec
d11             0.03000000 sec
TD0                      1

======== CHANNEL f1 ========
NUC1                   13C
P1                    8.07 usec
PL1                  -6.00 dB
SFO1           100.6196894 MHz

======== CHANNEL f2 ========
CPDPRG[2           waltz16
NUC2                    1H
PCPD2                80.00 usec
PL2         0 dB
PL12                 18.00 dB
SFO2           400.1318006 MHz

F2 - Processing parameters
SI                  131072
SF             100.6127580 MHz
WDW                     EM
SSB         0
LB                    0.80 Hz
GB          0
PC                    0.50
```

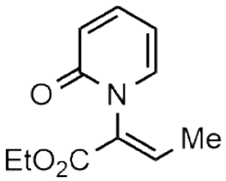

3v

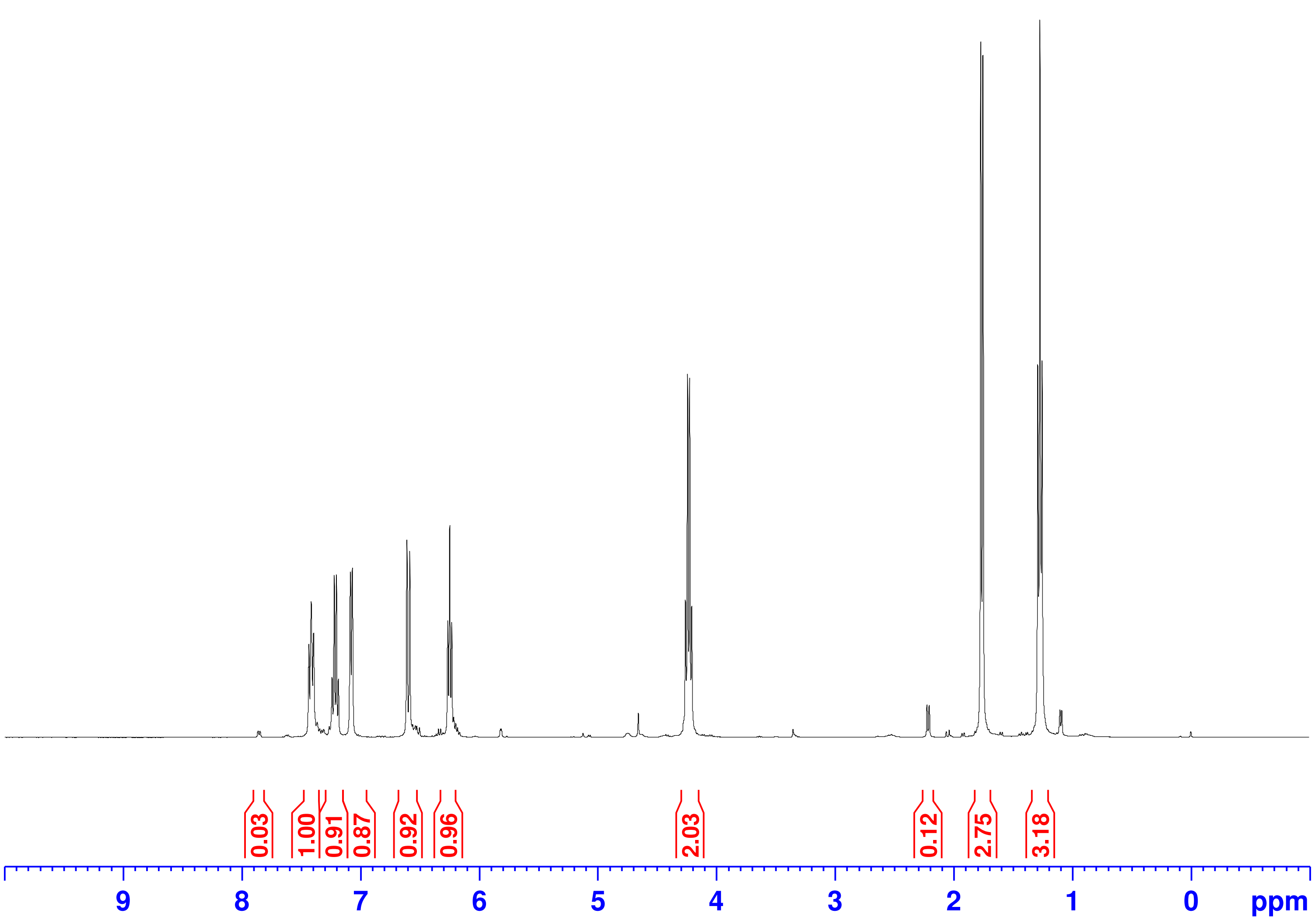


```
Current Data Parameters
NAME        1H gns-12-040
EXPNO                   1
PROCNO                  1

F2 - Acquisition Parameters
Date_            20210302
Time                17.03
INSTRUM             spect
PROBHD   5 mm Multinucl
PULPROG              zg30
TD                  32768
SOLVENT             CDCl3
NS                     16
DS                      0
SWH              8012.820 Hz
FIDRES           0.244532 Hz
AQ              2.0447233 sec
RG                   28.5
DW                 62.400 usec
DE                   6.50 usec
TE                  295.2 K
D1             0.01000000 sec
TD0                     1

======== CHANNEL f1 ========
NUC1                   1H
P1                   7.20 usec
PL1                 -5.00 dB
SFO1          400.1332010 MHz

F2 - Processing parameters
SI                 131072
SF            400.1299518 MHz
WDW                    EM
SSB       0
LB                   0.25 Hz
GB        0
PC                   0.20
```

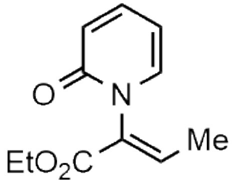

3v

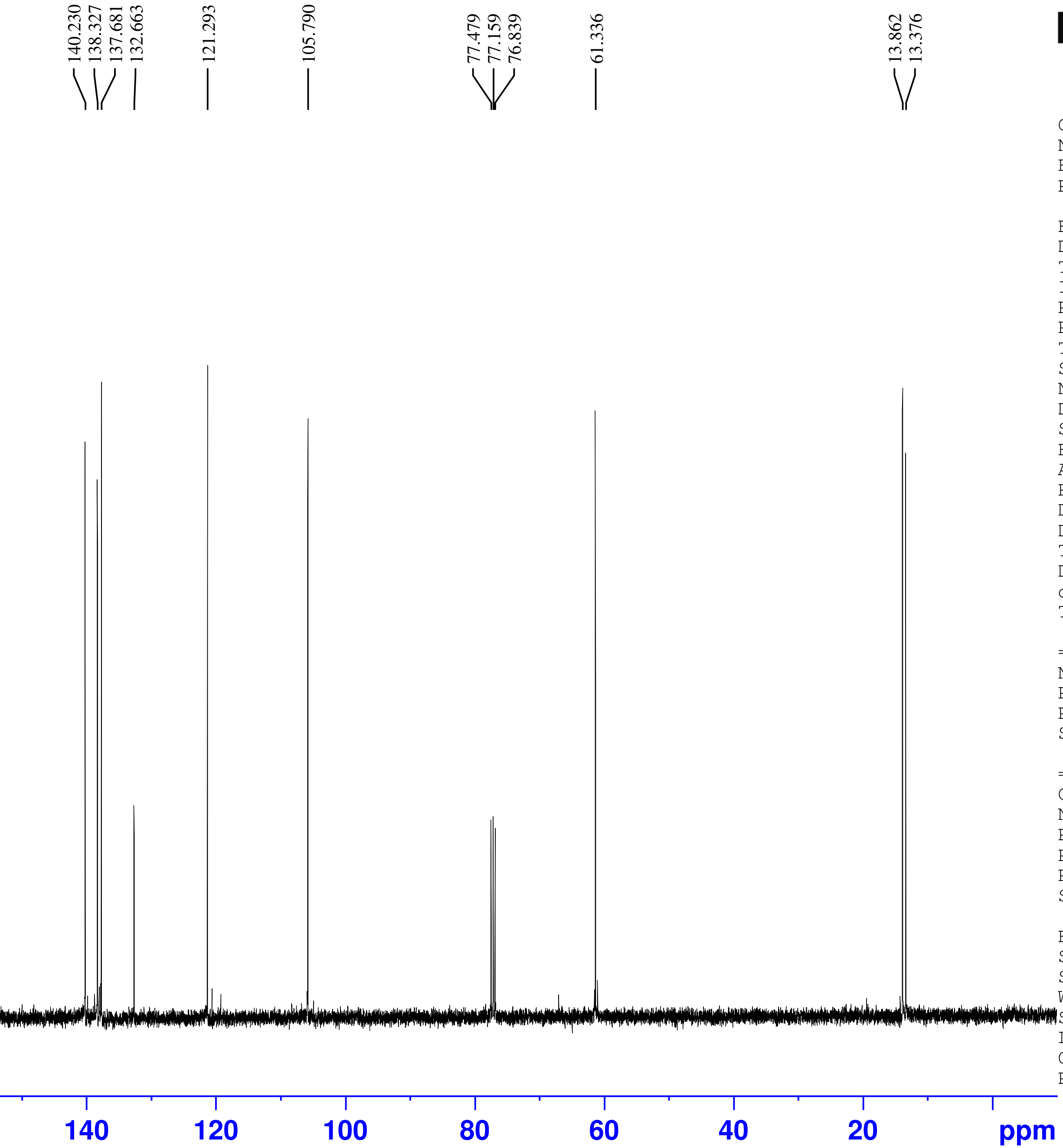


```
Current Data Parameters
NAME       13C gns-12-040
EXPNO                   1
PROCNO                  1

F2 - Acquisition Parameters
Date_            20210302
Time                17.06
INSTRUM             spect
PROBHD     5 mm Multinucl
PULPROG           zgdc30
TD                  65536
SOLVENT             CDCl3
NS                     47
DS                      4
SWH             26246.719 Hz
FIDRES           0.400493 Hz
AQ              1.2484608 sec
RG                  645.1
DW                 19.050 usec
DE                   6.50 usec
TE                  295.2 K
D1             0.69999999 sec
d11            0.03000000 sec
TD0                     1

======== CHANNEL f1 ========
NUC1                  13C
P1                   8.07 usec
PL1                 -6.00 dB
SFO1          100.6196894 MHz

======== CHANNEL f2 ========
CPDPRG[2          waltz16
NUC2                   1H
PCPD2               80.00 usec
PL2        0 dB
PL12                18.00 dB
SFO2          400.1318006 MHz

F2 - Processing parameters
SI                 131072
SF            100.6127844 MHz
WDW                    EM
SSB        0
LB                   0.80 Hz
GB         0
PC                   0.50
```

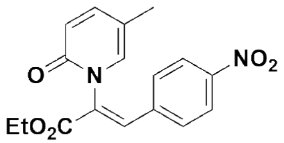

5a

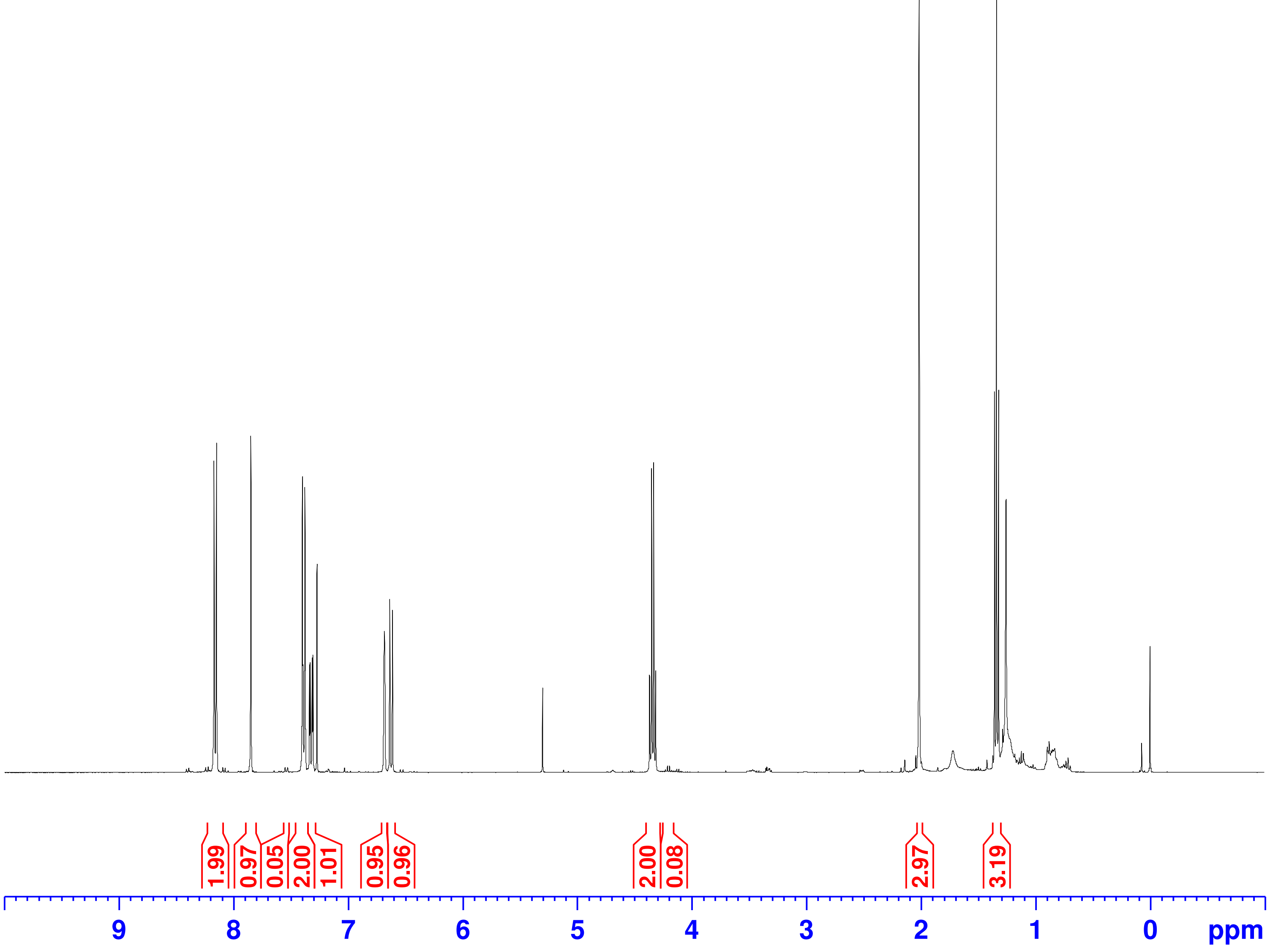

BRUKER

```
Current Data Parameters
NAME      1H gns-11-142 2
EXPNO                   1
PROCNO                  1

F2 - Acquisition Parameters
Date_            20210123
Time                18.43 h
INSTRUM             spect
PROBHD   Z104450_0192 (
PULPROG              zg30
TD                  65536
SOLVENT             CDCl3
NS                     16
DS                      2
SWH              8012.820 Hz
FIDRES           0.244532 Hz
AQ              4.0894465 sec
RG                    161
DW                 62.400 usec
DE                  16.92 usec
TE                  298.0 K
D1             1.00000000 sec
TD0                     1
SFO1          400.1324708 MHz
NUC1                   1H
P0                   5.00 usec
P1                  15.00 usec
PLW1           8.47000027 W

F2 - Processing parameters
SI                  65536
SF            400.1300049 MHz
WDW                    EM
SSB      0
LB                   0.30 Hz
GB       0
PC                   1.00
```

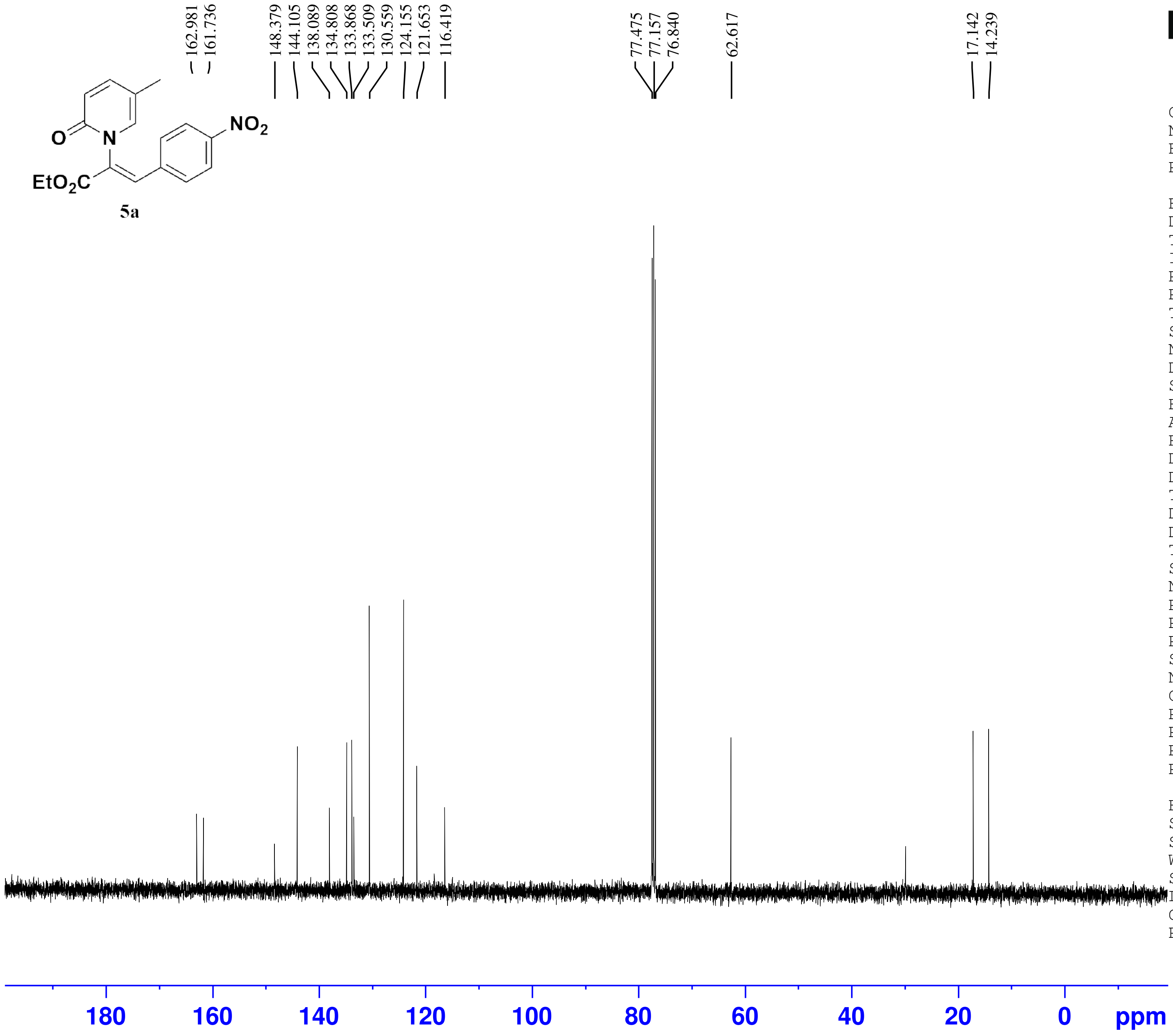

BRUKER

```
Current Data Parameters
NAME      13C gns-11-142 2
EXPNO                    1
PROCNO                   1

F2 - Acquisition Parameters
Date_             20210123
Time                 19.03 h
INSTRUM              spect
PROBHD   Z104450_0192 (
PULPROG            zgpg30
TD                  65536
SOLVENT             CDCl3
NS                    100
DS                      2
SWH             24038.461 Hz
FIDRES           0.733596 Hz
AQ              1.3631488 sec
RG                    181
DW                 20.800 usec
DE                   6.50 usec
TE                  298.2 K
D1             2.00000000 sec
D11            0.03000000 sec
TD0                     1
SFO1          100.6228298 MHz
NUC1                  13C
P0                   3.28 usec
P1                   9.85 usec
PLW1          28.63999939 W
SFO2          400.1316005 MHz
NUC2                   1H
CPDPRG[2          waltz65
PCPD2               90.00 usec
PLW2           8.47000027 W
PLW12          0.23528001 W
PLW13          0.11834000 W

F2 - Processing parameters
SI                  32768
SF            100.6127572 MHz
WDW                    EM
SSB       0
LB                   1.00 Hz
GB        0
PC                   1.40
```

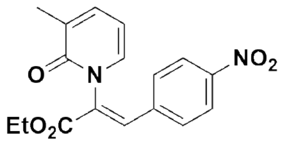

5b

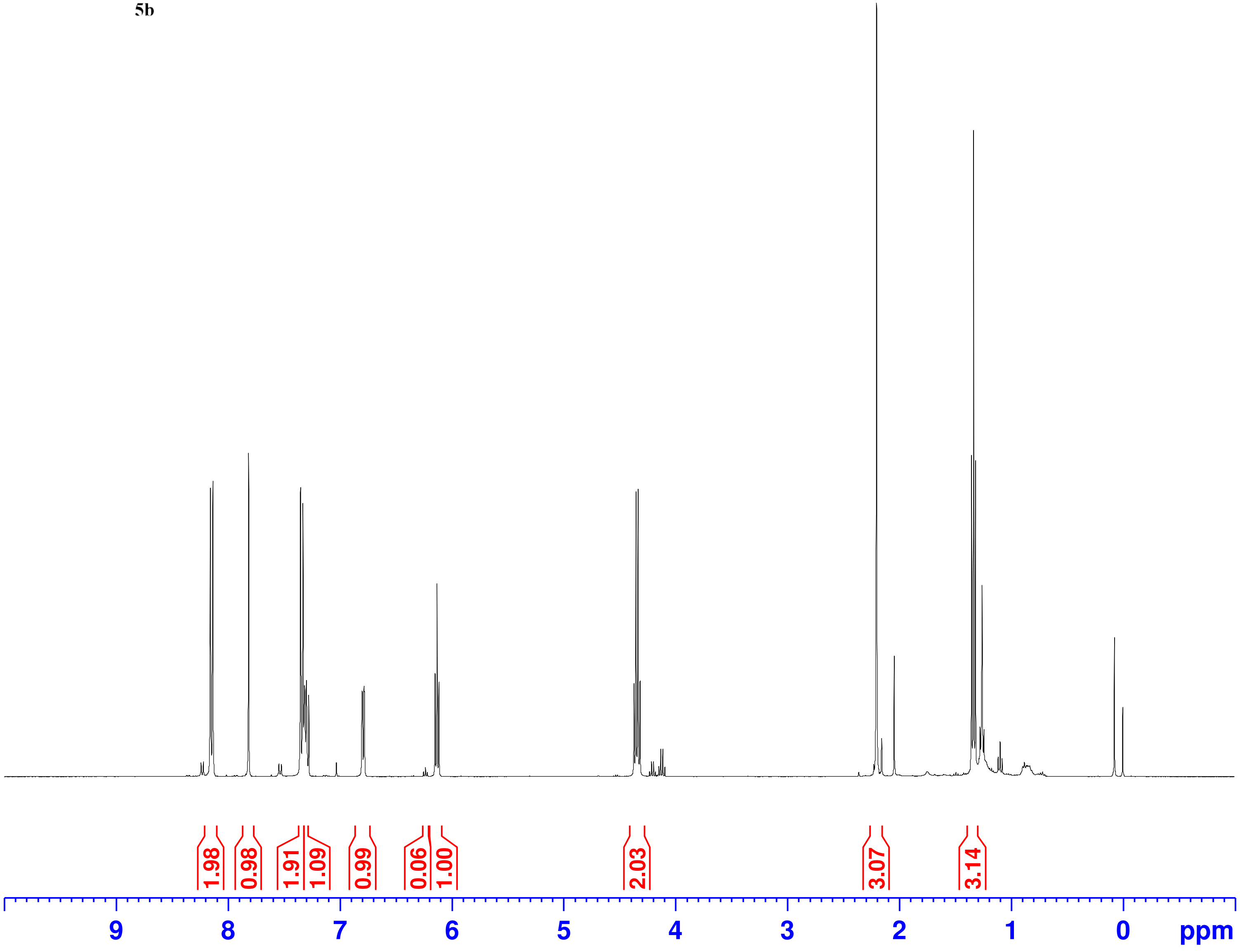

Current Data Parameters
NAME 1H gns-11-192 2
EXPNO 2
PROCNO 1

F2 - Acquisition Parameters
Date_ 20210218
Time 16.52 h
INSTRUM spect
PROBHD Z104450_0192 (
PULPROG zg30
TD 65536
SOLVENT CDCl3
NS 16
DS 2
SWH 8012.820 Hz
FIDRES 0.244532 Hz
AQ 4.0894465 sec
RG 71.8
DW 62.400 usec
DE 16.92 usec
TE 298.4 K
D1 1.00000000 sec
TD0 1
SFO1 400.1324708 MHz
NUC1 1H
P0 5.00 usec
P1 15.00 usec
PLW1 8.47000027 W

F2 - Processing parameters
SI 65536
SF 400.1300027 MHz
WDW EM
SSB 0
LB 0.30 Hz
GB 0
PC 1.00

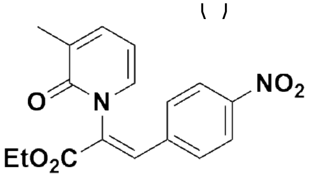

5b

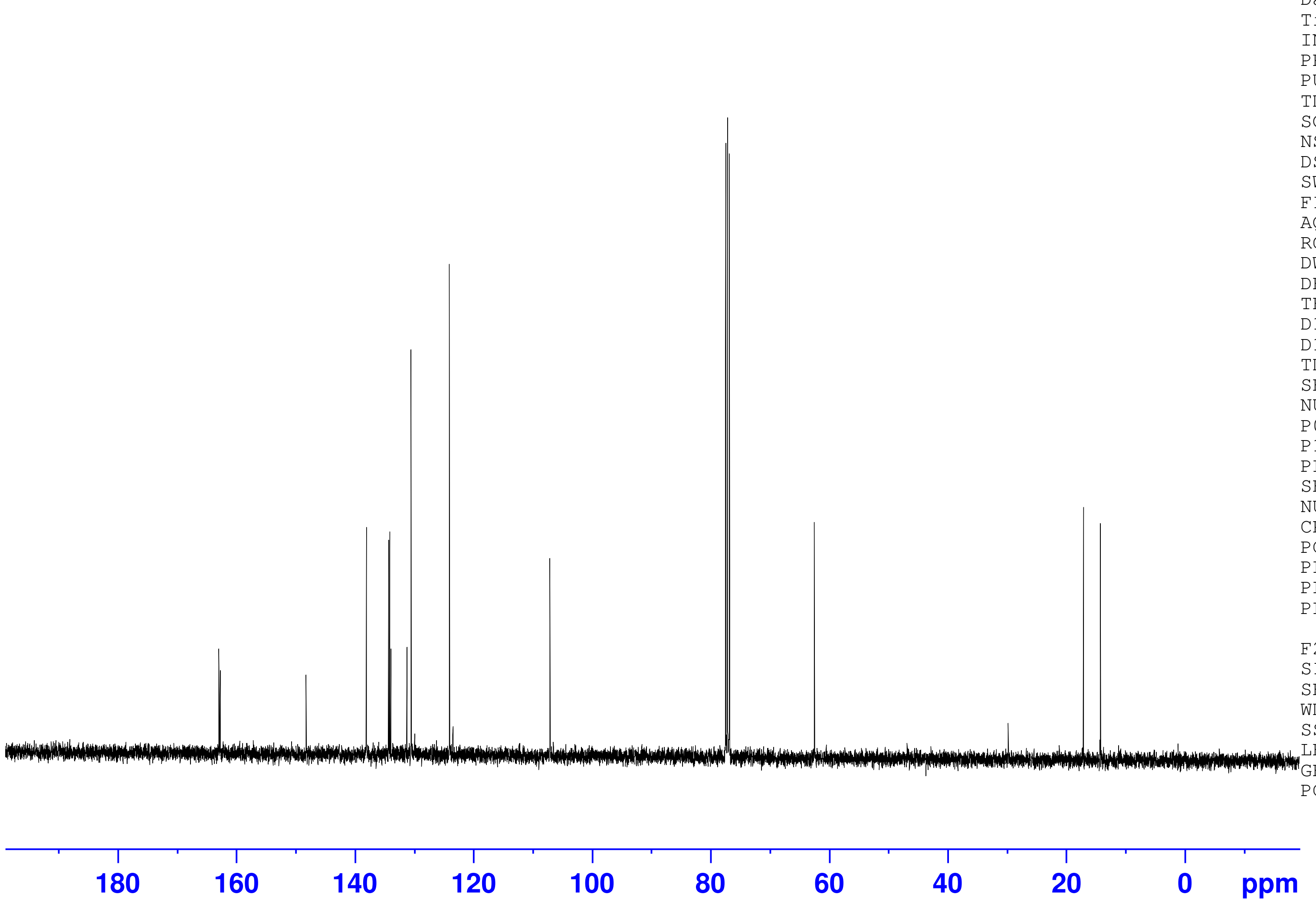

BRUKER

Current Data Parameters
NAME 13C gns-11-192 2
EXPNO 1
PROCNO 1

F2 - Acquisition Parameters
Date_ 20210218
Time 17.01 h
INSTRUM spect
PROBHD Z104450_0192 (
PULPROG zgpg30
TD 65536
SOLVENT CDCl3
NS 100
DS 2
SWH 24038.461 Hz
FIDRES 0.733596 Hz
AQ 1.3631488 sec
RG 203
DW 20.800 usec
DE 6.50 usec
TE 298.8 K
D1 2.00000000 sec
D11 0.03000000 sec
TD0 1
SFO1 100.6228298 MHz
NUC1 13C
P0 3.28 usec
P1 9.85 usec
PLW1 28.63999939 W
SFO2 400.1316005 MHz
NUC2 1H
CPDPRG[2 waltz65
PCPD2 90.00 usec
PLW2 8.47000027 W
PLW12 0.23528001 W
PLW13 0.11834000 W

F2 - Processing parameters
SI 32768
SF 100.6127586 MHz
WDW EM
SSB 0
LB 1.00 Hz
GB 0
PC 1.40

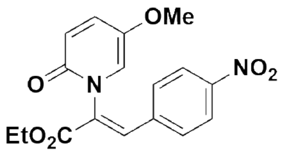

5c

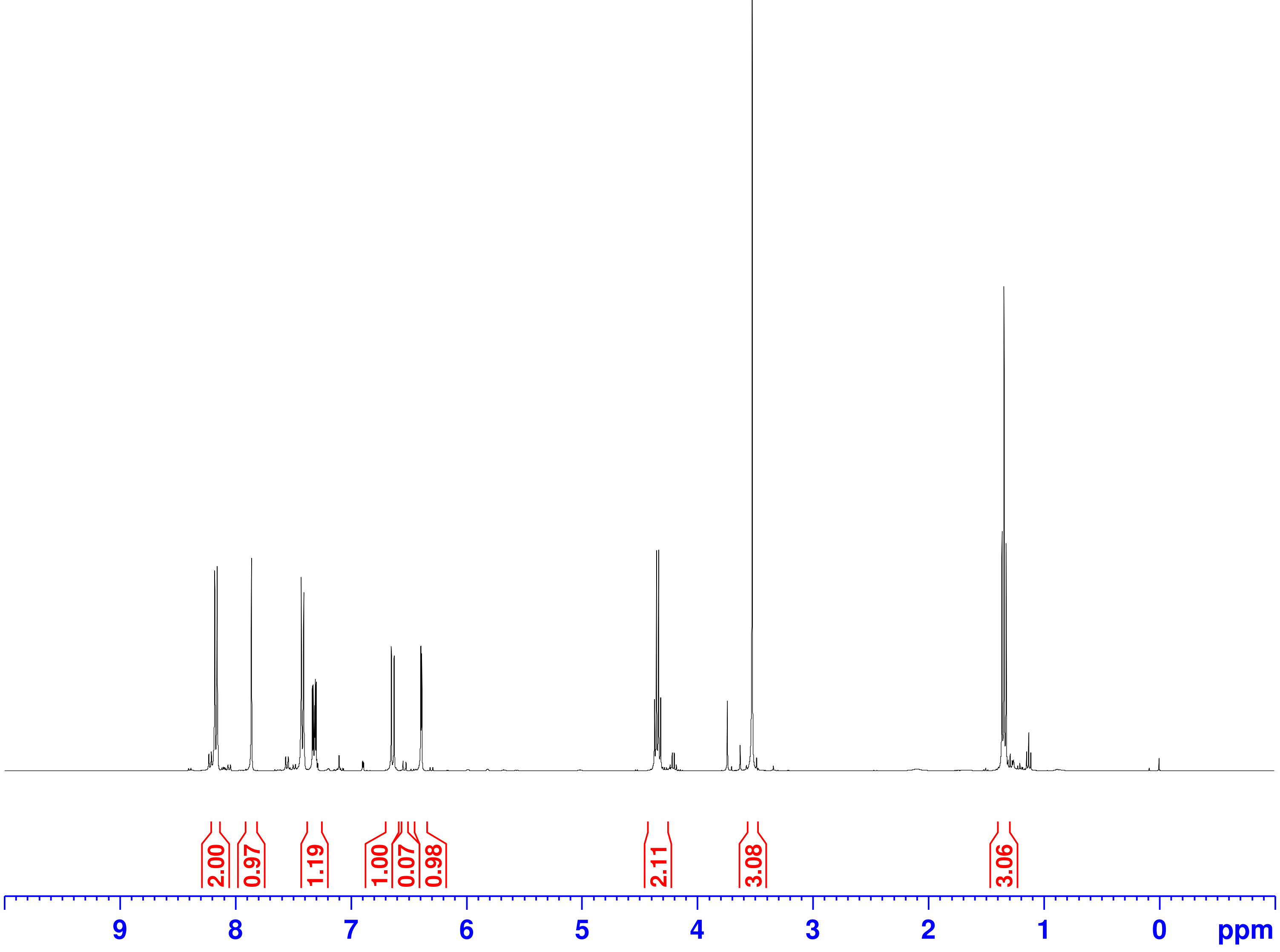


```
Current Data Parameters
NAME       1H gns-12-094 2
EXPNO                    1
PROCNO                   1

F2 - Acquisition Parameters
Date_             20210329
Time                 14.30
INSTRUM              spect
PROBHD   5 mm Multinucl
PULPROG               zg30
TD                   32768
SOLVENT              CDCl3
NS                      16
DS                       0
SWH              8012.820 Hz
FIDRES           0.244532 Hz
AQ              2.0447233 sec
RG                      57
DW                  62.400 usec
DE                    6.50 usec
TE                   295.2 K
D1             0.01000000 sec
TD0                      1

======== CHANNEL f1 ========
NUC1                    1H
P1                    7.20 usec
PL1                  -5.00 dB
SFO1           400.1332010 MHz

F2 - Processing parameters
SI                  131072
SF             400.1299879 MHz
WDW                     EM
SSB      0
LB                    0.25 Hz
GB       0
PC                    0.20
```

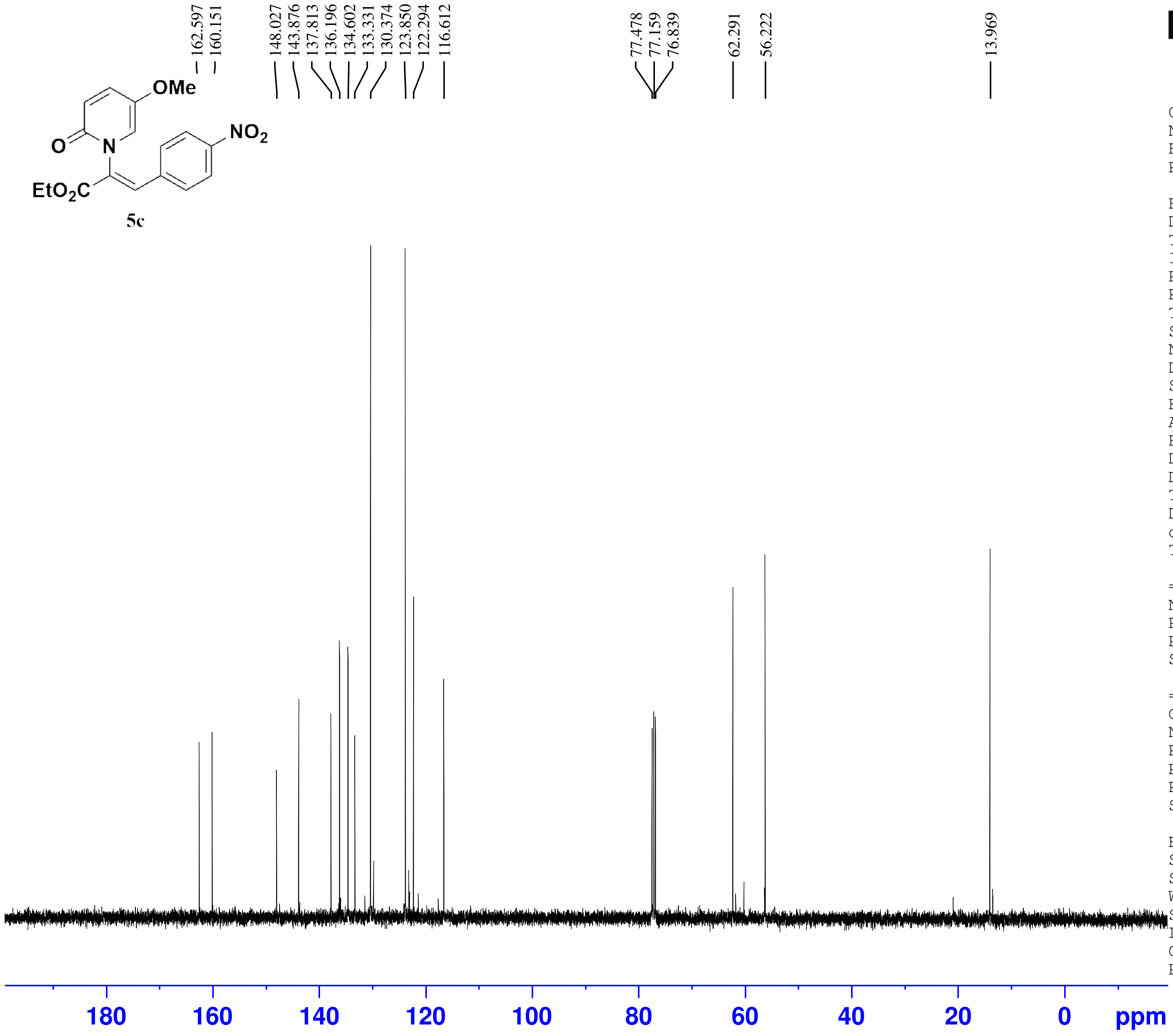

BRUKER

```
Current Data Parameters
NAME       13C gns-12-094
EXPNO                   1
PROCNO                  1

F2 - Acquisition Parameters
Date_            20210326
Time                14.50
INSTRUM             spect
PROBHD   5 mm Multinucl
PULPROG           zgdc30
TD                 65536
SOLVENT            CDCl3
NS                    51
DS                     4
SWH           26246.719 Hz
FIDRES         0.400493 Hz
AQ            1.2484608 sec
RG                1625.5
DW                19.050 usec
DE                  6.50 usec
TE                 295.2 K
D1            0.69999999 sec
d11           0.03000000 sec
TD0                    1

======== CHANNEL f1 ========
NUC1                 13C
P1                  8.07 usec
PL1                -6.00 dB
SFO1         100.6196894 MHz

======== CHANNEL f2 ========
CPDPRG[2         waltz16
NUC2                  1H
PCPD2              80.00 usec
PL2         0 dB
PL12               18.00 dB
SFO2         400.1318006 MHz

F2 - Processing parameters
SI                131072
SF           100.6127791 MHz
WDW                   EM
SSB         0
LB                  0.80 Hz
GB          0
PC                  0.50
```

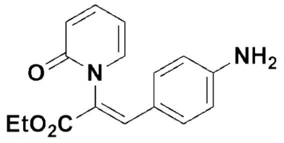

7

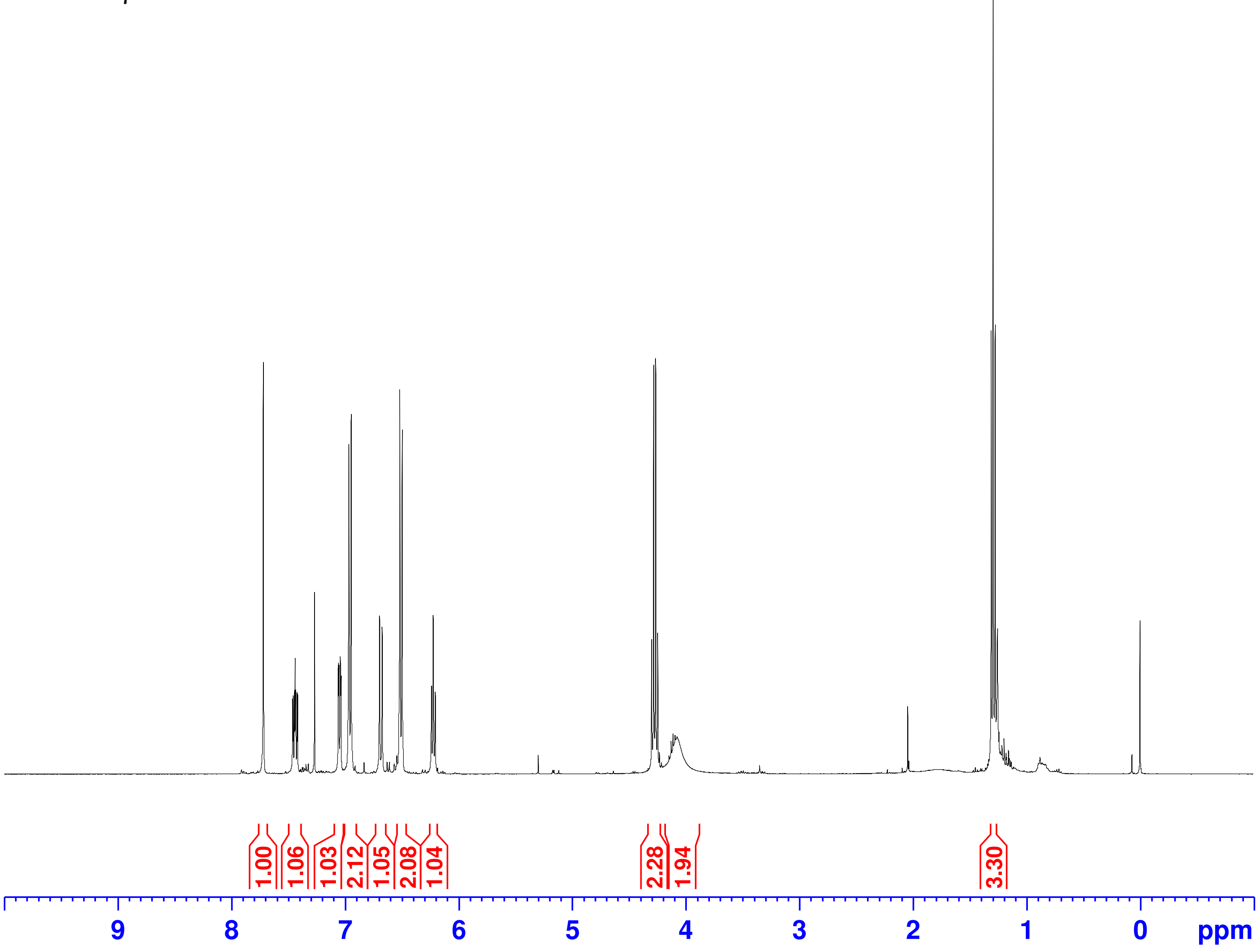


```
Current Data Parameters
NAME       1H gns-12-054
EXPNO                  1
PROCNO                 1

F2 - Acquisition Parameters
Date_           20210310
Time               14.38
INSTRUM            spect
PROBHD    5 mm Multinucl
PULPROG             zg30
TD                 32768
SOLVENT            CDCl3
NS                    16
DS                     0
SWH             8012.820 Hz
FIDRES          0.244532 Hz
AQ             2.0447233 sec
RG                 161.3
DW                62.400 usec
DE                  6.50 usec
TE                 295.2 K
D1            0.01000000 sec
TD0                    1

======== CHANNEL f1 ========
NUC1                  1H
P1                  7.20 usec
PL1                -5.00 dB
SFO1         400.1332010 MHz

F2 - Processing parameters
SI                131072
SF           400.1300058 MHz
WDW                   EM
SSB      0
LB                  0.25 Hz
GB       0
PC                  0.20
```

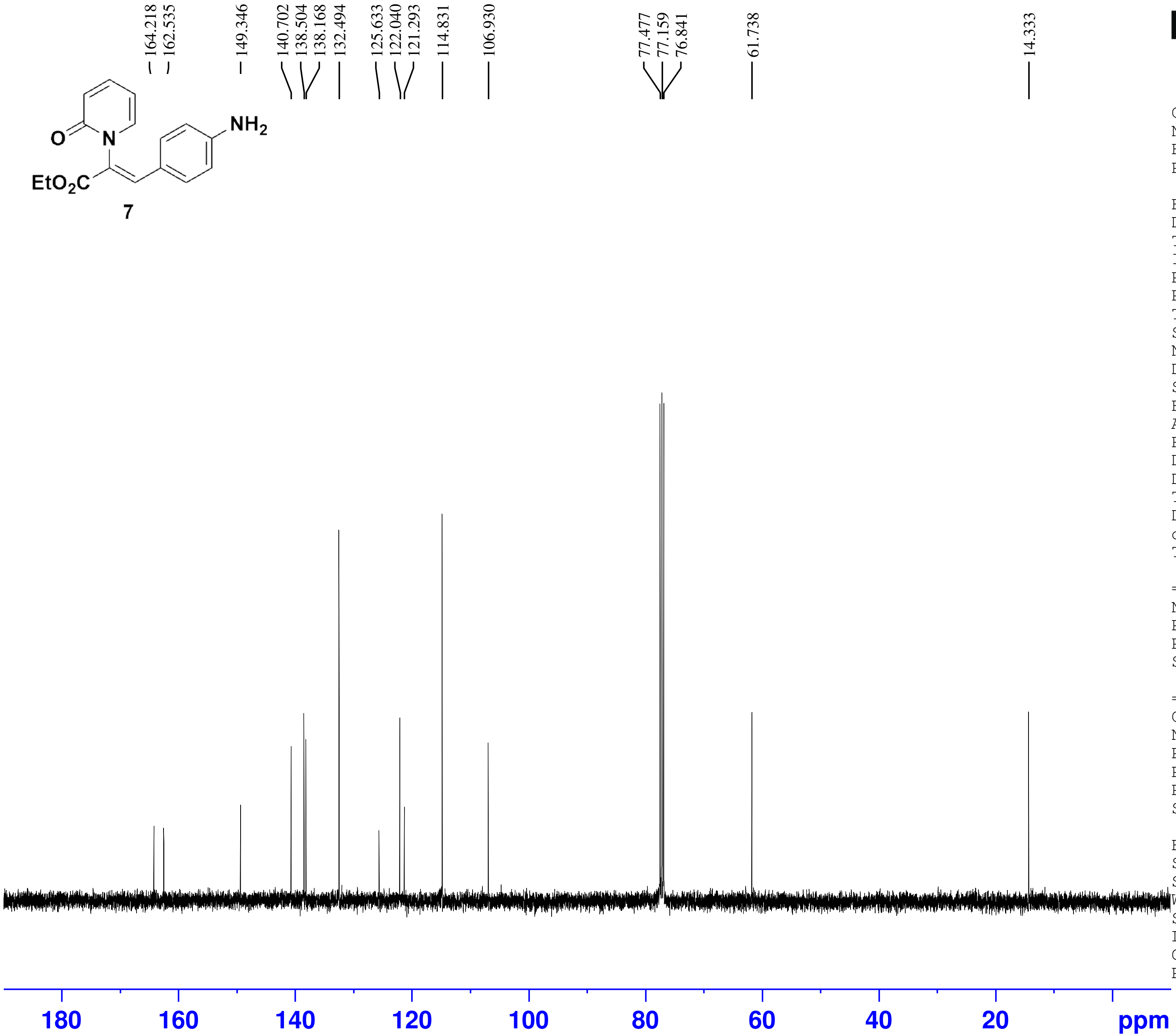


```
Current Data Parameters
NAME       13C gns-12-054
EXPNO                   1
PROCNO                  1

F2 - Acquisition Parameters
Date_            20210310
Time                14.50
INSTRUM             spect
PROBHD     5 mm Multinucl
PULPROG           zgdc30
TD                 65536
SOLVENT            CDCl3
NS                   300
DS                     4
SWH            26246.719 Hz
FIDRES          0.400493 Hz
AQ             1.2484608 sec
RG                 287.4
DW                19.050 usec
DE                  6.50 usec
TE                 295.2 K
D1            0.69999999 sec
d11           0.03000000 sec
TD0                    1

======== CHANNEL f1 ========
NUC1                 13C
P1                  8.07 usec
PL1                -6.00 dB
SFO1         100.6196894 MHz

======== CHANNEL f2 ========
CPDPRG[2         waltz16
NUC2                  1H
PCPD2              80.00 usec
PL2        0 dB
PL12               18.00 dB
SFO2         400.1318006 MHz

F2 - Processing parameters
SI                131072
SF           100.6127585 MHz
WDW                   EM
SSB        0
LB                  0.80 Hz
GB         0
PC                  0.50
```

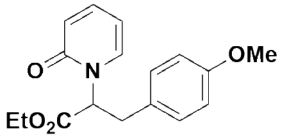

8

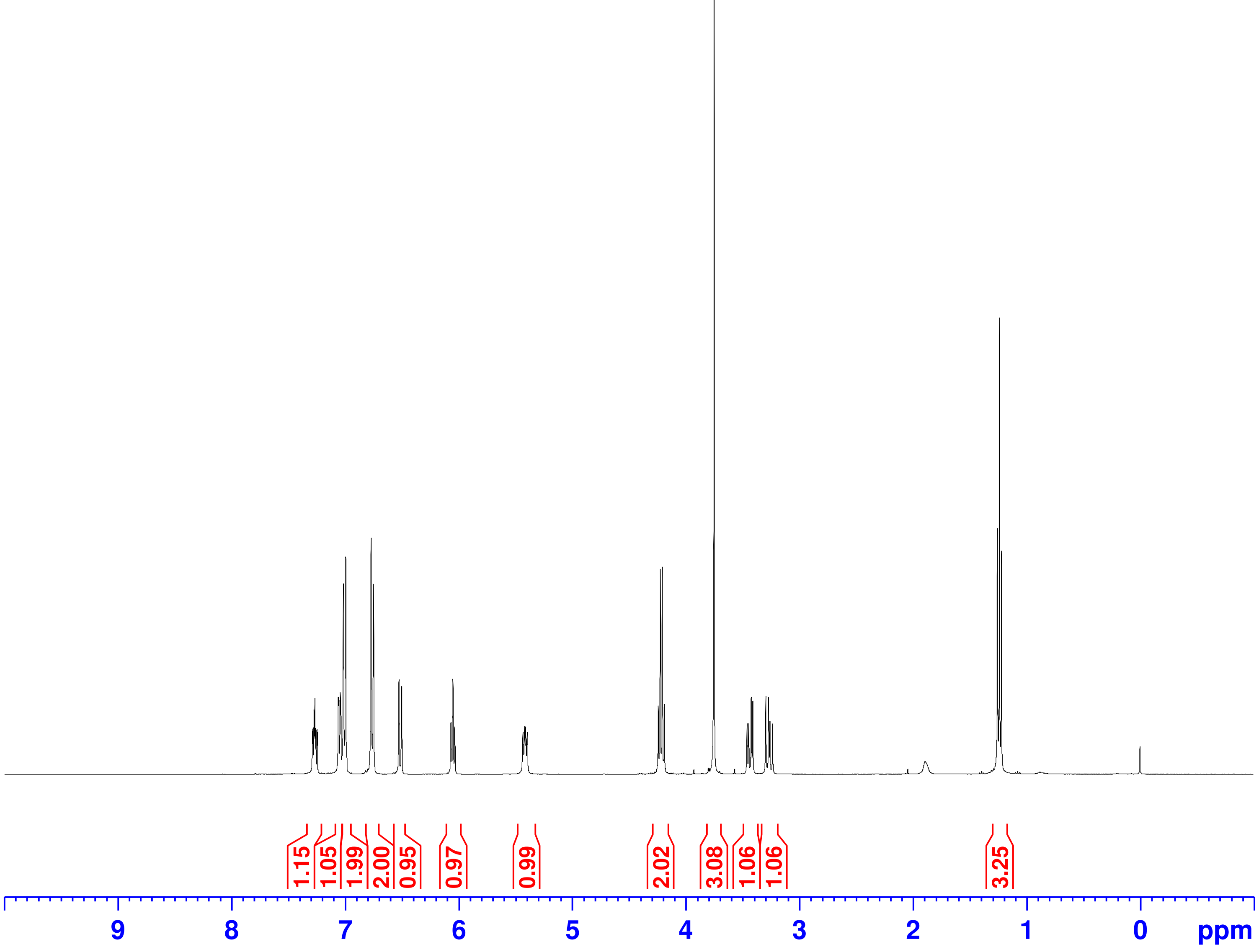


```
Current Data Parameters
NAME       1H gns-12-106 2
EXPNO                    1
PROCNO                   1

F2 - Acquisition Parameters
Date_             20210405
Time                 15.38
INSTRUM              spect
PROBHD    5 mm Multinucl
PULPROG               zg30
TD                   32768
SOLVENT              CDCl3
NS                      16
DS                       0
SWH               8012.820 Hz
FIDRES            0.244532 Hz
AQ               2.0447233 sec
RG                   101.6
DW                  62.400 usec
DE                    6.50 usec
TE                   295.2 K
D1              0.01000000 sec
TD0                      1

======== CHANNEL f1 ========
NUC1                    1H
P1                    7.20 usec
PL1                  -5.00 dB
SFO1           400.1332010 MHz

F2 - Processing parameters
SI                  131072
SF             400.1300034 MHz
WDW                     EM
SSB      0
LB                    0.25 Hz
GB       0
PC                    0.20
```

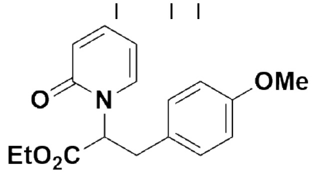

8

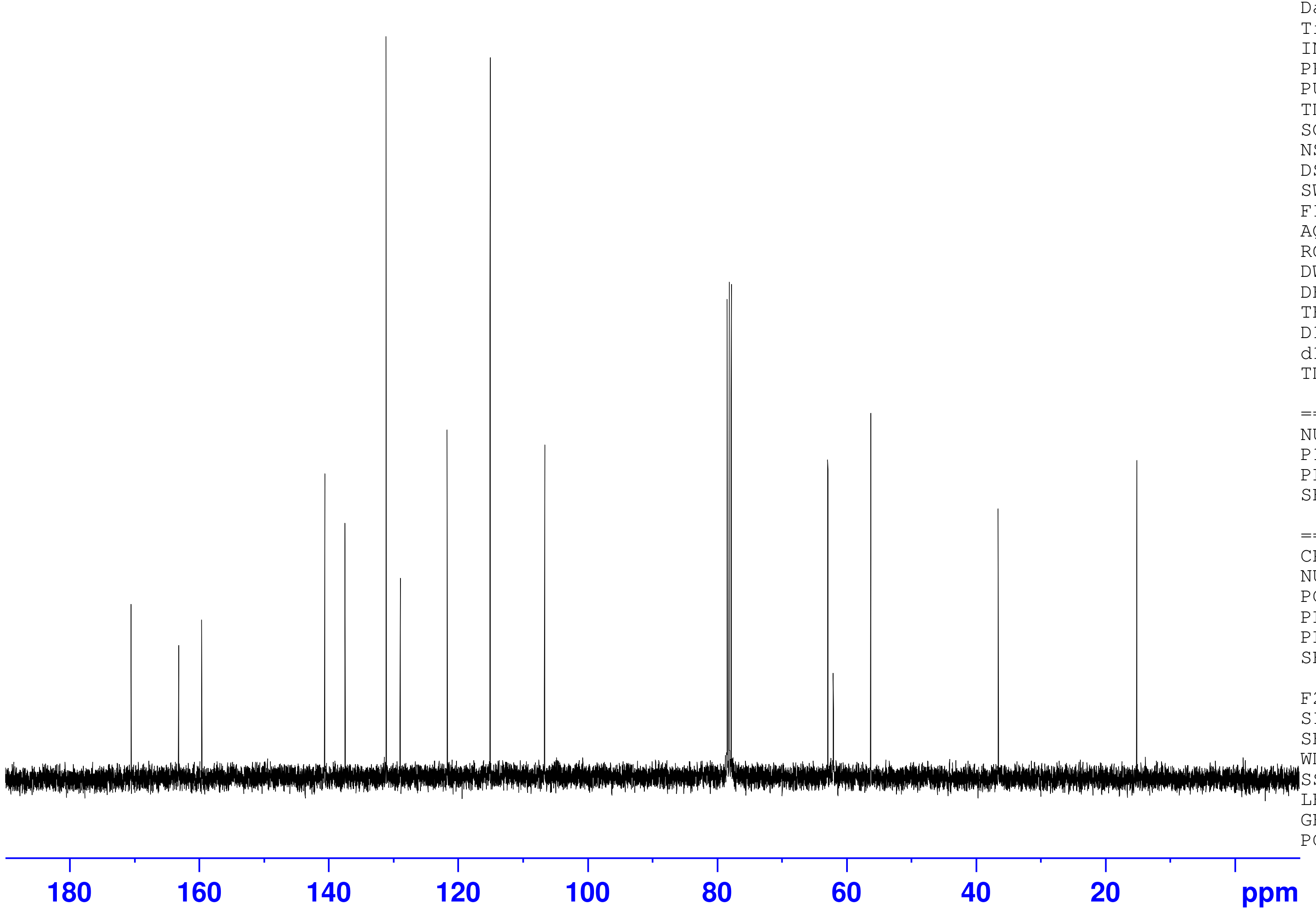


```
Current Data Parameters
NAME       13C gns-12-106 2
EXPNO                     1
PROCNO                    1

F2 - Acquisition Parameters
Date_              20210405
Time                  15.44
INSTRUM               spect
PROBHD    5 mm Multinucl
PULPROG             zgdc30
TD                    65536
SOLVENT               CDCl3
NS                      151
DS                        4
SWH               26246.719 Hz
FIDRES             0.400493 Hz
AQ                1.2484608 sec
RG                    645.1
DW                   19.050 usec
DE                     6.50 usec
TE                    295.2 K
D1               0.69999999 sec
d11              0.03000000 sec
TD0                       1

======== CHANNEL f1 ========
NUC1                    13C
P1                     8.07 usec
PL1                   -6.00 dB
SFO1            100.6196894 MHz

======== CHANNEL f2 ========
CPDPRG[2            waltz16
NUC2                     1H
PCPD2                 80.00 usec
PL2         0 dB
PL12                  18.00 dB
SFO2            400.1318006 MHz

F2 - Processing parameters
SI                   131072
SF              100.6126631 MHz
WDW                      EM
SSB         0
LB                     0.80 Hz
GB          0
PC                     0.50
```

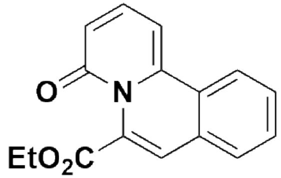

9

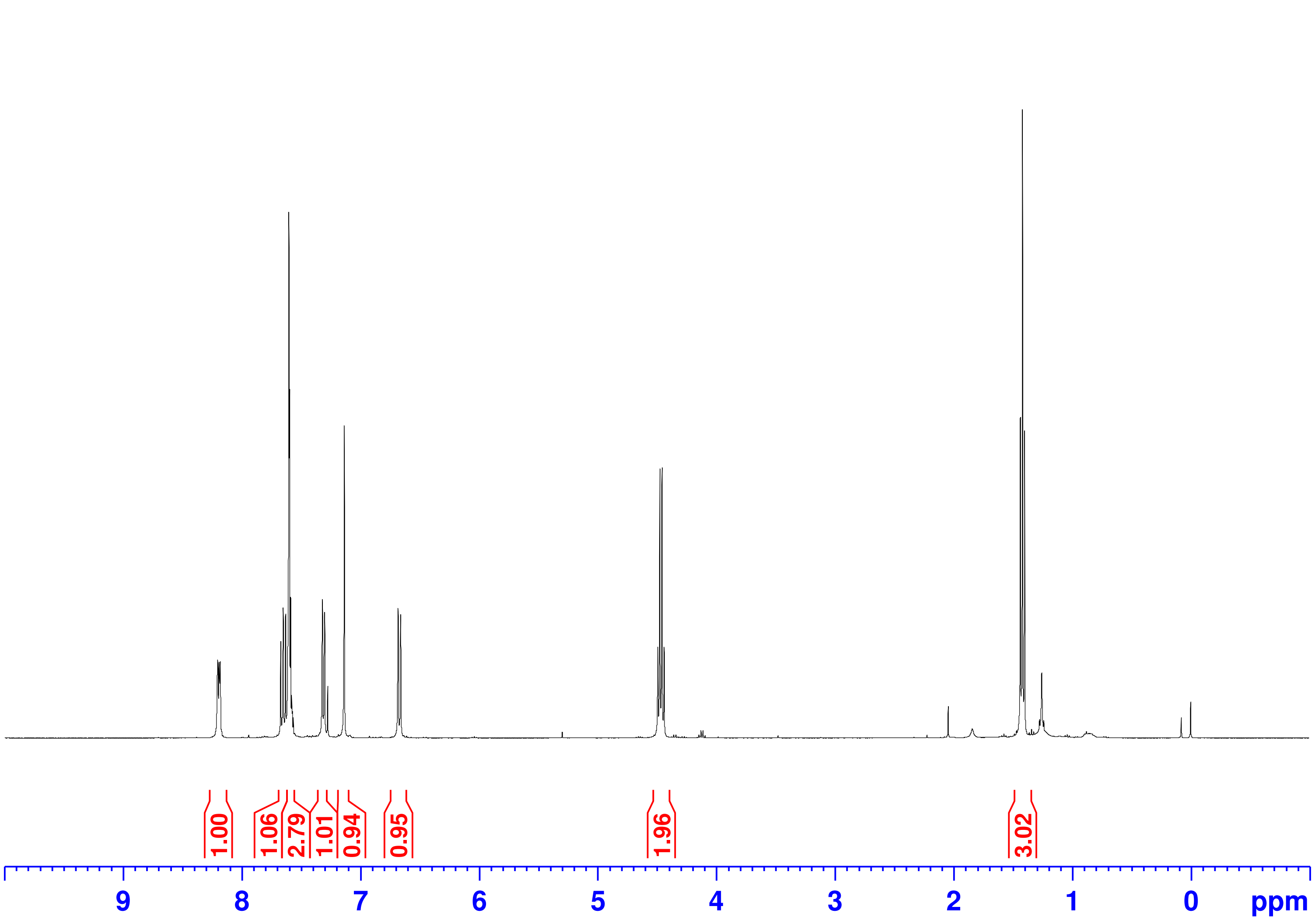


```
Current Data Parameters
NAME         1H gns-12-046
EXPNO                    1
PROCNO                   1

F2 - Acquisition Parameters
Date_             20210302
Time                 17.18
INSTRUM              spect
PROBHD    5 mm Multinucl
PULPROG               zg30
TD                   32768
SOLVENT              CDCl3
NS                      16
DS                       0
SWH               8012.820 Hz
FIDRES            0.244532 Hz
AQ               2.0447233 sec
RG                     128
DW                  62.400 usec
DE                    6.50 usec
TE                   295.2 K
D1              0.01000000 sec
TD0                      1

======== CHANNEL f1 ========
NUC1                    1H
P1                    7.20 usec
PL1                  -5.00 dB
SFO1           400.1332010 MHz

F2 - Processing parameters
SI                  131072
SF             400.1300031 MHz
WDW                     EM
SSB       0
LB                    0.25 Hz
GB        0
PC                    0.20
```

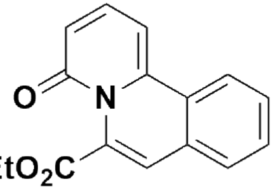

9

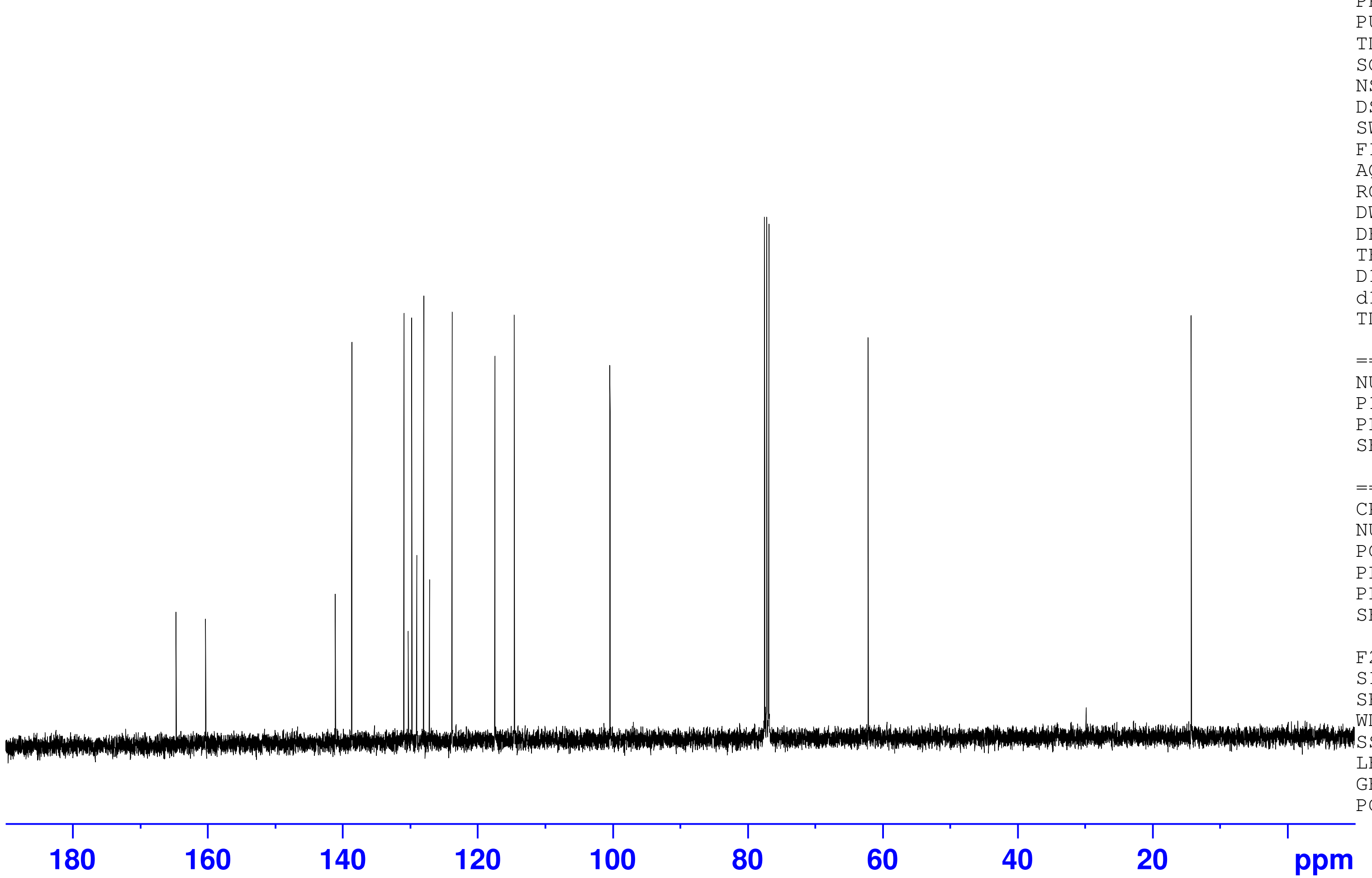


```
Current Data Parameters
NAME       13C gns-12-046
EXPNO                   1
PROCNO                  1

F2 - Acquisition Parameters
Date_            20210302
Time                17.29
INSTRUM             spect
PROBHD   5 mm Multinucl
PULPROG           zgdc30
TD                  65536
SOLVENT             CDCl3
NS                    300
DS                      4
SWH             26246.719 Hz
FIDRES           0.400493 Hz
AQ              1.2484608 sec
RG                 1625.5
DW                 19.050 usec
DE                   6.50 usec
TE                  295.2 K
D1             0.69999999 sec
d11            0.03000000 sec
TD0                     1

======== CHANNEL f1 ========
NUC1                  13C
P1                   8.07 usec
PL1                 -6.00 dB
SFO1          100.6196894 MHz

======== CHANNEL f2 ========
CPDPRG[2          waltz16
NUC2                   1H
PCPD2               80.00 usec
PL2         0 dB
PL12                18.00 dB
SFO2          400.1318006 MHz

F2 - Processing parameters
SI                 131072
SF            100.6127606 MHz
WDW                    EM
SSB         0
LB                   0.80 Hz
GB          0
PC                   0.50
```
